# Hydrothermal regimes regulate microbiome patterns across vertical profile and their responses to alpine permafrost degradation

**DOI:** 10.1038/s41467-026-77448-6

**Published:** 2026-09-09

**Authors:** Shengyun Chen, Ali Bahadur, Jiahui Zhu, Enyan Liu, Yuzheng Gu, Hewei Liang, Wenbo Zhang, Shicai Li, Aowei Li, Peijie Wei, Qianyu Liu, Tonghua Wu, Peizhi Yang, Yuanqiang Zou, Mo Han, Lucie A. Malard

**Affiliations:** 1 State Key Laboratory of Cryospheric Science and Frozen Soil Engineering/Qilian Mountains Glacier, Frozen Soil and Ecohydrology Research Station, Northwest Institute of Eco-Environment and Resources, Chinese Academy of Sciences, Lanzhou, 730000 China; 2 College of Ecology, Lanzhou University, Lanzhou, 730000 China; 3 University of Chinese Academy of Sciences, Beijing, 100049 China; 4 BGI Research, Sanya, 572025 China; 5 State Key Laboratory of Genome and Multi-omics Technologies, BGI Research, Shenzhen, 518083 China; 6 College of Grassland Agriculture, Northwest A&F University, Yangling, 712100 China; 7 Cryosphere Research Station on the Qinghai–Tibet Plateau, State Key Laboratory of Cryospheric Science and Frozen Soil Engineering, Northwest Institute of Eco-Environment and Resources, Chinese Academy of Sciences, Lanzhou, 730000 China; 8 Shenzhen Key Laboratory of Environmental Microbial Genomics and Application, BGI Research, Shenzhen, 518083 China; 9 Department F.-A. Forel for environmental and aquatic sciences, University of Geneva, Geneva, 1211 Switzerland

**Keywords:** Cryospheric science, Microbial ecology

## Abstract

Deciphering microbiomes across vertical profiles is critical for understanding biogeochemical cycling and potential biosafety hazards associated with permafrost degradation under climate warming. However, knowledge about microbiome patterns over key profile layers in response to degradation remains limited in the Qinghai–Tibet Plateau. Using metagenomic data obtained from 150 samples of six 15 m-depth alpine permafrost cores along a degradation gradient, we analyzed microbial community structure and functional potential across different main-layers, including the active, frozen fringe, and frozen layers. We found the recovered microbial and functional diversity decreased with profile depth, and declined only in the active layer as permafrost degraded. Interestingly, *Pithoviridae, Caulimoviridae*, and virulence factors related to adhesion, biofilm formation, and immune regulation were enriched in the frozen fringe layer, along with increasing relative abundance of *Lavidaviridae* under the degradation. Along the degradation gradient, carbohydrate-active enzymes diversity decreased in the active layer, while the ratio of nitrite reductase genes to nitrous oxide reductase genes increased in the active and the frozen fringe layers. Hydrothermal regimes emerged as the primary controls shaping microbiome distributions across the vertical profile and along the degradation gradient. Notably, hydrothermal and microbiome attributes jointly regulated carbon/nitrogen loss during the degradation. Taken together, these findings offer crucial insights into microbiome patterns, carbon/nitrogen loss and biosecurity concerning permafrost degradation under global warming.

## Abstract (web summary)

Here, the authors surveyed the vertical microbiome in the Qinghai-Tibet Plateau and assessed the degradation patterns, biogeochemical cycling, and nutrient loss across these stratified microbiomes.

## Introduction

Permafrost, an essential component of the Earth’s terrestrial cryosphere, refers to subsurface material (soil or rock) that remains at or below 0 °C for at least two consecutive years^1^. Based on the division of permafrost stratigraphic profile, it mainly comprises the active layer, the frozen layer^1,2^, and the frozen fringe or transitional layer situated at the boundary between these two layers^3,4^. Permafrost covers about one-fifth of the land surface in the Northern Hemisphere^5^, predominantly in high-latitude and high-altitude regions^6,7^. The Qinghai–Tibet Plateau (QTP), often known as the Earth’s “Third Pole”, is the world’s highest altitude and largest areas of continuous alpine permafrost in the mid-low latitudes^8,9^. Containing ~14 Pg (1 Pg = 10^15 ^g) of carbon and 1.8 Pg of nitrogen within a depth of 3 meters^10,11^, the QTP permafrost region represents a substantial reservoir, thus playing major roles in carbon and nitrogen sequestration. Numerous observations have shown extensive degradation of permafrost driven by global warming, marked by increasing active layer thickness (ALT) and rising ground temperature^6,12^. These variations are especially evident in the QTP and are anticipated to become more pronounced in the future^6,13^. Such degradation increasingly threatens the structure and function of alpine ecosystems that rely on permafrost stability^14,15^. In particular, previously sequestered organic matter decomposed by microorganisms is released as greenhouse gases, thereby intensifying the potential positive feedback to climate warming^16–18^. Meanwhile, degradation may also revive viruses preserved in permafrost, exacerbating potential biosafety risks^19,20^. Given these adverse effects, an in-depth understanding of the mechanisms regulating carbon/nitrogen loss within the different profile layers in response to permafrost degradation, coupled with the assessment of biosecurity, is critical to maintain alpine ecosystem functions and human health.

As key drivers of biogeochemical processes, microorganisms play an indispensable role in soil element cycling^21,22^. Despite the harsh environments such as subzero temperatures and oligotrophic conditions, permafrost harbors a diverse array of microorganisms, which perform essential ecological functions^21,23^. Recent advances in biotechnology have made metagenomic sequencing a prevailing approach to permafrost microbiome studies, enabling genome-resolved analyses of uncultivated taxa and revealing the extensive functional potential of microbial communities^24,25^. However, previous research has focused predominantly on high-latitude permafrost regions^26,27^, with little attention to the alpine permafrost regions of the QTP. A few studies have reported pronounced variations with depth in permafrost microbiomes, including differences in microbial and functional diversity, taxonomic composition, and genetic potential related to carbon (e.g., pectin, hemicellulose, and cellulose degradation) and nitrogen (e.g., nitrate reduction and denitrification) cycling^28–30^. Nevertheless, the empirical evidence in the deeper layers, especially the frozen fringe and frozen layers, is sparse. This scarcity can be primarily attributed to the difficulties in delineating the main-layers of vertical profile without the monitoring of permafrost temperature, then obtaining samples and extracting DNA from the deeper low-biomass frozen layer^31^. As expected, there are marked differences in hydrothermal regimes and nutrient status from the active through the frozen fringe to the frozen layers, with temperature, water content, and nutrients exhibiting a progressive reduction^3,32^. Consequently, these distinct habitats across the main profile layers should result in distributional disparities in microbial community structure and metabolic function, but our understanding of their distribution and the controlling factors remains very limited.

Microorganisms have gained extensive attention due to their crucial regulatory roles in carbon and nitrogen cycling and the potential increase in pathogenic hazards as permafrost degrades^21,33,34^. Evidence indicates that degradation reduces microbial diversity and stability in the surface-active layer^14,35^, while community composition shifts toward more metabolically active communities^33,36^. Concurrently, the functional genes involved in decomposition of complex organic carbon and nitrogen metabolic pathways increase in abundance amid degradation^33,37^, which are associated with carbon and nitrogen losses^14,38,39^. These variation patterns likely reflect microbiome responses to degradation-driven fluctuations in hydrothermal regimes, redox conditions, and nutrients^40^. Despite these advances in understanding the microbial community structure and functional attributes of the active layer in response to permafrost degradation, their responses in the frozen fringe and frozen layers are still unexplored to date. It is also worth emphasizing that viral communities are highly diverse in permafrost^41^, and some viruses harbor genes encoding virulence factors (VFs) and antibiotic resistance genes (ARGs)^42,43^, which can profoundly affect host community dynamics^44^. Permafrost degradation may release ancient viruses into the environment, as evidenced by experimental revival of 30,000-year-old *Pithovirus sibericum* and *Mollivirus sibericum* from Siberia^20,45^. However, little is known about the diversity of permafrost viromes and their response to degradation across different layers in the QTP. In view of the aforementioned issues, there is an urgent need to comprehensively understand whether microbiomes at different main-layers exhibit differential responses to degradation, and to assess their relationship with carbon/nitrogen storage and the potential consequences for ecosystem functions.

**Fig. 1:**
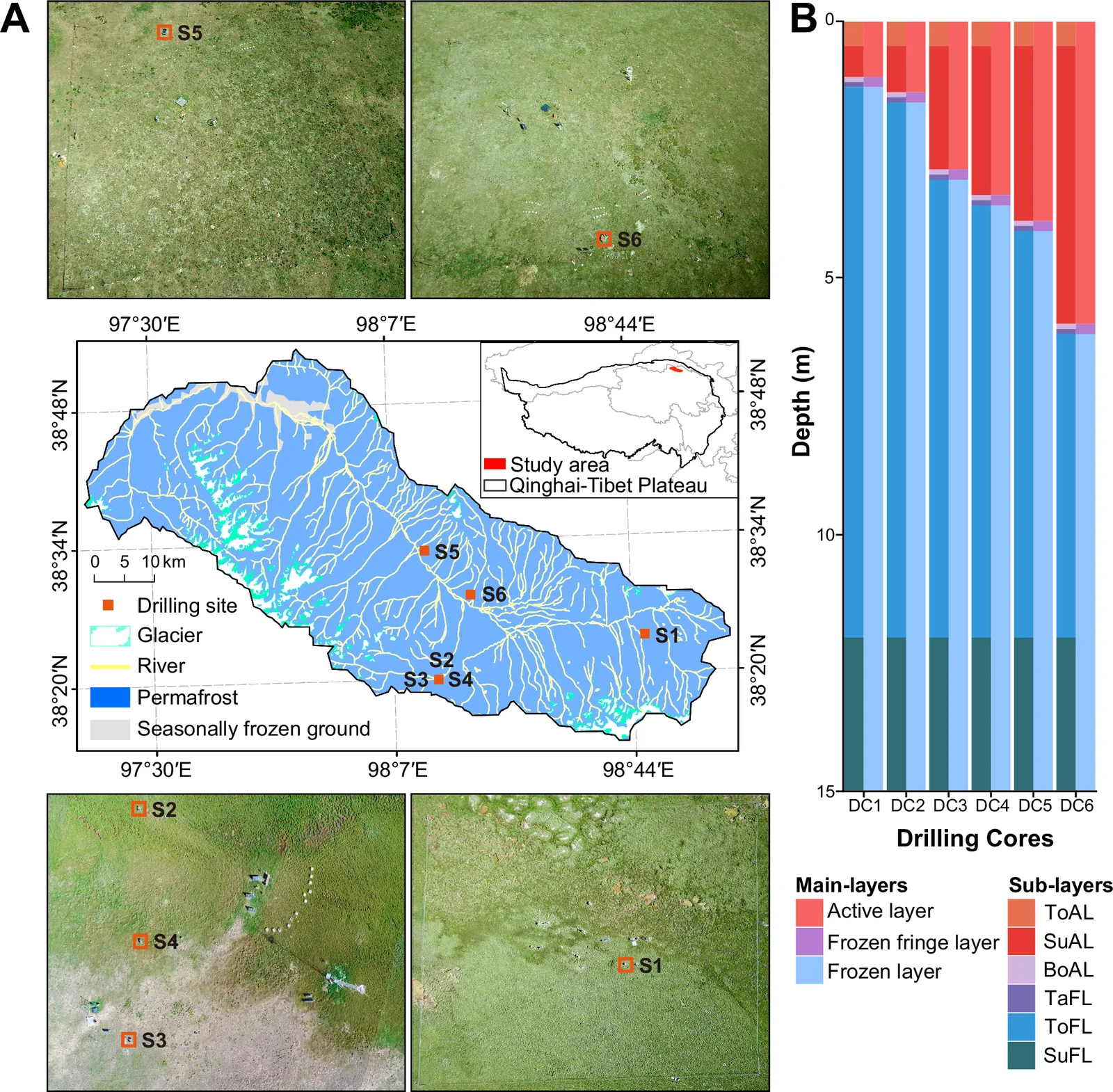
Geographic distribution of drilling sites. **A** Map of the Shule River Headwaters in the Qinghai–Tibet Plateau showing the locations of six drilling sites (S1–S6). The basemap comprises the boundary and the geographic location of the study area obtained from Qin et al.^121^, the boundary of the Qinghai–Tibet Plateau derived from Liu et al.^122^, and the distributions of permafrost and seasonally frozen ground obtained from Zou et al.^123^. No pre-existing or third-party basemap was used. Glacier data used in the production of this figure were obtained from the second glacial catalog data set of China (v1.0) (https://cstr.cn/CSTR:11738.11.ncdc.Westdc.2020.656). This dataset is licensed under the Creative Commons Attribution 4.0 International (CC BY 4.0) license (https://creativecommons.org/licenses/by/4.0/), and no changes were made to the original data. River data were extracted from digital elevation model (DEM) data acquired by the National Aeronautics and Space Administration (NASA) Shuttle Radar Topography Mission (SRTM) (https://www.earthdata.nasa.gov/data/instruments/srtm) using the “Hydrology” in the Spatial Analyst Tools of ArcGIS 10.8. NASA is acknowledged as the source of the SRTM data used in the production of this figure. In addition, four aerial images were reproduced from Chen et al.^46^ under the CC BY 4.0 license and were originally taken by the authors using DJI Mavic Pro unmanned aerial vehicle during the field survey. **B** Schematic representation of sampling depth distribution across the main-layers, sub-layers, and degradation gradient. The left column shows six sub-layers for each drilling core, and the right column represents three main-layers. ToAL top-active layer, SuAL sub-active layer, BoAL bottom-active layer, TaFL table-frozen layer, ToFL top-frozen layer, SuFL sub-frozen layer.

To address these critical knowledge gaps, we collected 150 samples from six 15-m drilling cores (DC1–DC6) in the Shule River headwaters on the northeastern margin of the QTP (Fig. 1). Specifically, the samples were collected over key profile layers, including the active (top- and sub-active layers), frozen fringe (bottom-active and table-frozen layers), and frozen layers (top- and sub- frozen layers). To characterize the extent of degradation, we obtained the permafrost degradation index (PDI) for each drilling site based on a principal component analysis (PCA) combining ALT and mean annual ground temperature (MAGT) at 15 m depth^46^, and ranked this index in ascending order to represent a degradation sequence from drilling sites 1 to 6 (Supplementary Fig. 1). We investigated microbial community composition, diversity, and functional attributes using metagenomics. Additionally, we measured environmental variables, including hydrothermal and chemical properties, to explore their influences on microbiomes. Our study aims to address the following scientific questions: (1) How do microbiomes vary across vertical profile? (2) Does the degradation significantly alter permafrost microbiomes within different layers? (3) Which environmental variables influence microbiome patterns, and especially how do these variables and microbiomes jointly regulate carbon/nitrogen loss under the degradation? Resolving these issues will lay the groundwork for future large-scale investigations into biotic and abiotic regulation of carbon/nitrogen loss within key layers as permafrost degrades.

## Results

### Microbial community composition and diversity across permafrost vertical profile

**Fig. 2:**
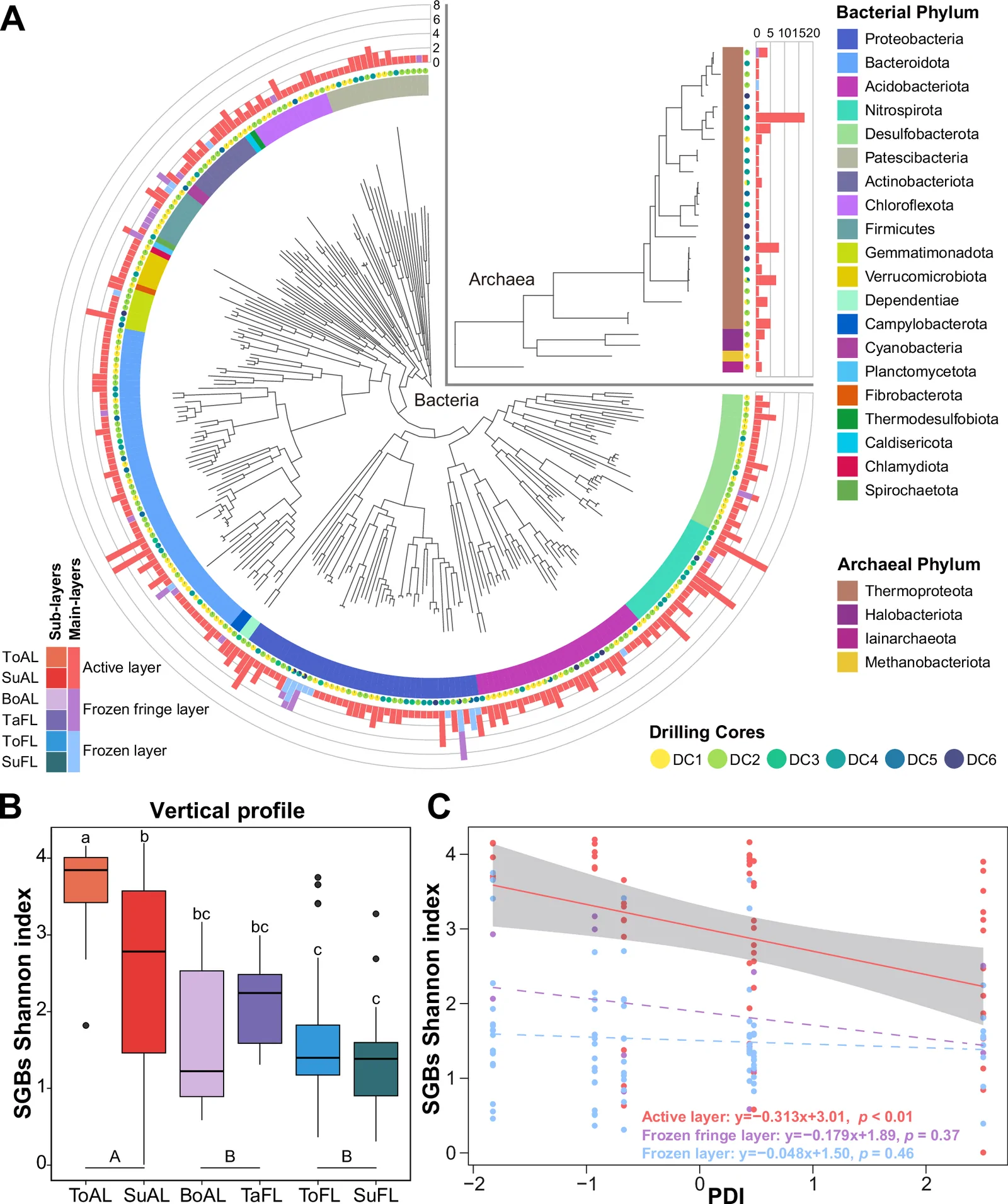
Permafrost prokaryotic community composition and diversity. **A** Phylogenetic trees were built from representative metagenome-assembled genomes (MAGs) of 282 species-level genome bins (SGBs) based on the Genome Taxonomy Database Toolkit (GTDB-Tk) with bacterial/archaeal default markers. The colored ring indicates the phylum level taxonomic classification of each SGB. The adjacent pie charts show the relative proportions of MAGs from different drilling cores (DC1–DC6) within each SGB. The outer bar graphs summarize the total number of MAGs associated with each main-layer. **B** Distribution of SGBs Shannon index across the main- and sub-layers (Active layer, *n* = 55, including ToAL, *n* = 18, and SuAL, *n* = 37; Frozen fringe layer, *n* = 12, including BoAL, *n* = 6, and TaFL, *n* = 6; Frozen layer, *n* = 83, including ToFL, *n* = 59, and SuFL, *n* = 24). Statistical significance is based on two-sided Kruskal–Wallis tests; lowercase letters indicate the significance of differences among the sub-layers (*p* < 0.05), while uppercase letters denote the significance of differences across the main-layers (*p* < 0.05). In the box plots, the central line indicates the median. The bounds of the box correspond to the 25th and 75th percentiles (interquartile range, IQR). The lower and upper whiskers extend to the minima and maxima within 1.5 × IQR, respectively. Points beyond the whiskers represent outliers. **C** Linear relationships between PDI and SGBs Shannon index across the main-layers. Solid lines indicate significant fitted ordinary least-squares regression models, with gray shaded areas denoting the 95% confidence intervals. Dashed lines indicate non-significant fitted ordinary least-squares regression models. PDI permafrost degradation index, ToAL top-active layer, SuAL sub-active layer, BoAL bottom-active layer, TaFL table-frozen layer, ToFL top-frozen layer, SuFL sub-frozen layer. Source data are provided as a Source Data file.

Shotgun metagenomic sequencing of 150 samples yielded 6.4 Tbp of sequencing data, enabling the assembly of 51.36 Gbp of contigs (Supplementary Data 1) and the recovery of 501 high- or medium-quality metagenome-assembled genomes (MAGs) on the basis of the criteria of Minimum Information about a Metagenome-Assembled Genome (MIMAG)^47^ (Supplementary Data 2). The assembly results showed substantial variation in the number and total length of contigs across samples, with a trend of fewer contigs recovered from deeper layers (frozen fringe and frozen layers). Most of these MAGs (441 of 501) were obtained from the active layer (Fig. 2A). The recovered MAGs were further clustered into 282 species-level genome bins (SGBs) based on a 95% average nucleotide identity (ANI) threshold, of which only 17 could be taxonomically annotated at species level referring to the Genome Taxonomy Database (GTDB; v2.3.2) (Fig. 2A and Supplementary Data 3), underscoring the vast number of unexplored microbial species in alpine permafrost of the QTP. Taxonomic annotation also revealed that Bacteroidota, Proteobacteria, Thermoproteota, Acidobacteriota, Desulfobacterota, Nitrospirota, and Patescibacteria were the dominant phyla (Supplementary Fig. 2 and Supplementary Data 4, and Supplementary Data 5). The bacterial phylum Bacteroidota and the archaeal phylum Thermoproteota dominated the prokaryotic community in the active layer, whereas Proteobacteria displayed higher relative abundance in the deeper layers (Supplementary Data 5). PCA and permutational multivariate analysis of variance (PERMANOVA) revealed that prokaryotic composition differed significantly across main- and sub-layers (Supplementary Fig. 3A). Among all examined environmental variables, pH and permafrost temperature (Tp) showed the highest correlations with community composition (Supplementary Fig. 3A). The Shannon index of the recovered SGBs was markedly higher in the active layer, with a notable decrease observed from the top-active layer to the sub-active layer (Fig. 2B). Notably, the hydrothermal regime stood out as the predominant environmental variable modulating the SGBs Shannon index across the vertical profile, with permafrost water content (PWC) and Tp explaining 34.1% and 30.6% of the variance, respectively (Supplementary Fig. 4A).

**Fig. 3:**
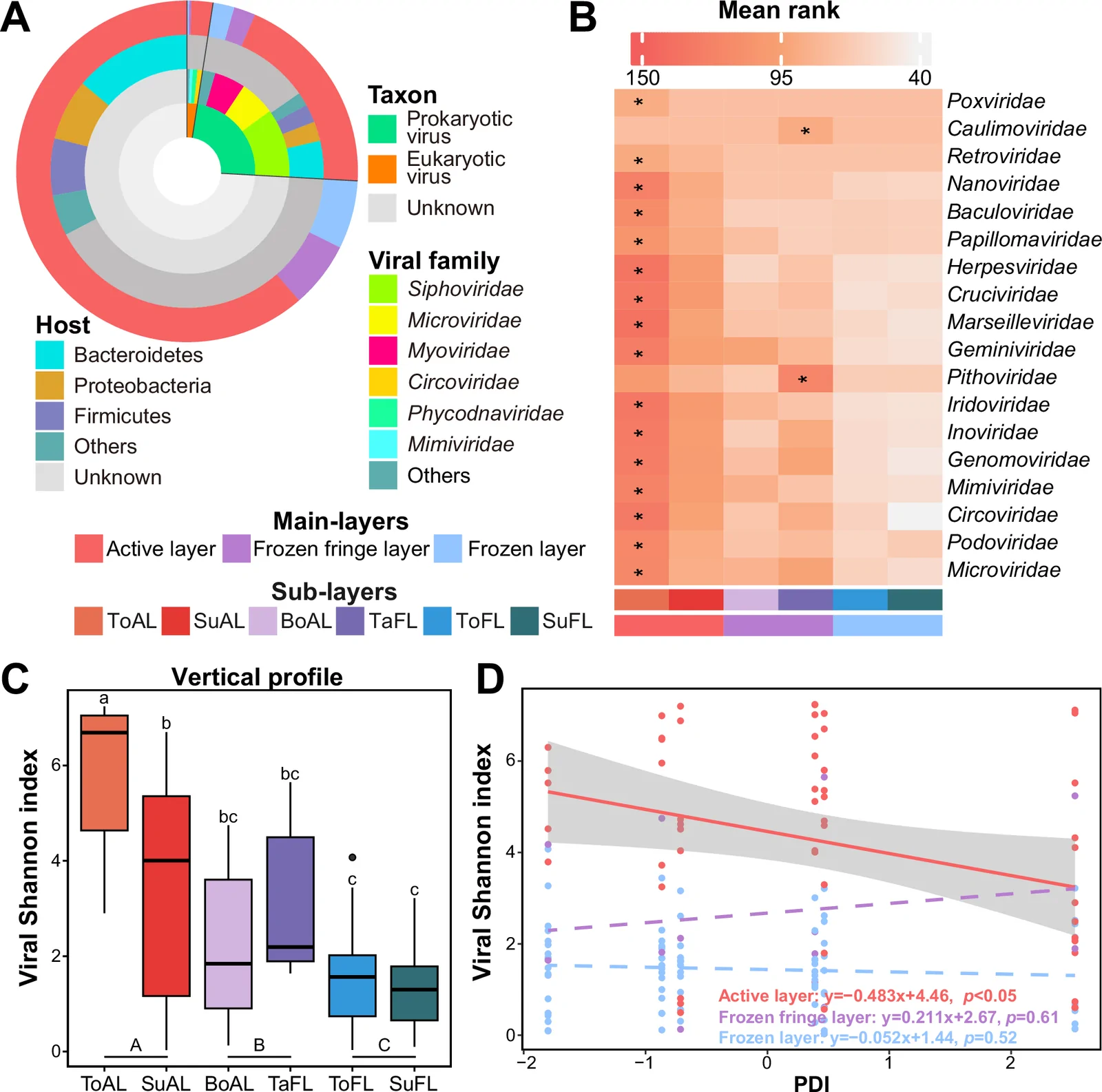
Permafrost viral community composition and diversity. **A** Proportions of viral families and their prokaryotic host identified across the main-layers. 1st annulus, proportions of viral taxa; 2nd annulus, proportion of viral families; 3rd annulus, hosts of prokaryotic viruses; 4th annulus, distribution of viral family across the main-layers. **B** Enrichment of viral families across the main- and sub-layers. Asterisks indicate statistical significance (determined by two-sided Kruskal–Wallis test, *p < 0.05). **C** Distribution of viral Shannon index across the main- and sub-layers (Active layer, *n* = 55, including ToAL, *n* = 18, and SuAL, *n* = 37; Frozen fringe layer, *n* = 12, including BoAL, *n* = 6, and TaFL, *n* = 6; Froze*n* layer, *n* = 83, including ToFL, *n* = 59, and SuFL, *n* = 24). Statistical significance is based on two-sided Kruskal–Wallis tests; lowercase letters indicate the significance of differences among the sub-layers (*p* < 0.05), while uppercase letters denote the significance of differences across the main-layers (*p* < 0.05). In the box plots, the central line indicates the median. The bounds of the box correspond to the 25th and 75th percentiles (interquartile range, IQR). The lower and upper whiskers extend to the minima and maxima within 1.5 × IQR, respectively. Points beyond the whiskers represent outliers. **D** Linear relationships between PDI and viral Shannon index across the main-layers. Solid lines indicate significant fitted ordinary least-squares regression models, with gray shaded areas denoting the 95% confidence intervals. Dashed lines indicate non-significant fitted ordinary least-squares regression models. PDI permafrost degradation index, ToAL top-active layer, SuAL sub-active layer, BoAL bottom-active layer, TaFL table-frozen layer, ToFL top-frozen layer, SuFL sub-frozen layer. Source data are provided as a Source Data file.

Our analysis enabled the recovery of 28,008 viral genomes and revealed distinct vertical distribution patterns of viral communities. A total of 82.82% of the viral genomes were derived from the active layer, whereas 8.66% and 8.52% of the total were recovered from the frozen fringe layer and the frozen layer, respectively (Fig. 3A). Taxonomic classification revealed that 23.45% of the viruses were prokaryotic viruses, primarily belonging to *Siphoviridae*, *Microviridae*, and *Myoviridae*. Hosts of these viruses mainly belonged to Bacteroidota, Proteobacteria, and Firmicutes (Fig. 3A). By comparison, only 2.44% of the total were classified as eukaryotic viruses, predominantly affiliated with *Circoviridae*, *Phycodnaviridae*, and *Mimiviridae* (Fig. 3A and Supplementary Data 5). However, 74.11% of the viral genomes could not be annotated at the family taxonomic level, indicating a vast number of undiscovered and uncharacterized viruses in alpine permafrost habitats. Viral community composition also exhibited pronounced variations across the vertical profile, with pH identified as the strongest determinant (Supplementary Fig. 3B). In particular, *Pithoviridae* and *Caulimoviridae* were enriched in the frozen fringe layer (table-frozen layer) (Fig. 3B, Supplementary Data 5, and Supplementary Data 6). The Shannon index of the recovered viral genomes significantly decreased across the main- and sub-layers, with the highest value occurring in the active layer, followed by the frozen fringe and frozen layers (Fig. 3C). Importantly, hydrothermal regime explained the largest proportion of variation in viral Shannon index (46.1%) throughout the vertical profile, followed by pH (45.8% of the variance explained) (Supplementary Fig. 4B).

### Functional attributes of permafrost microbiomes over key profile layers

**Fig. 4:**
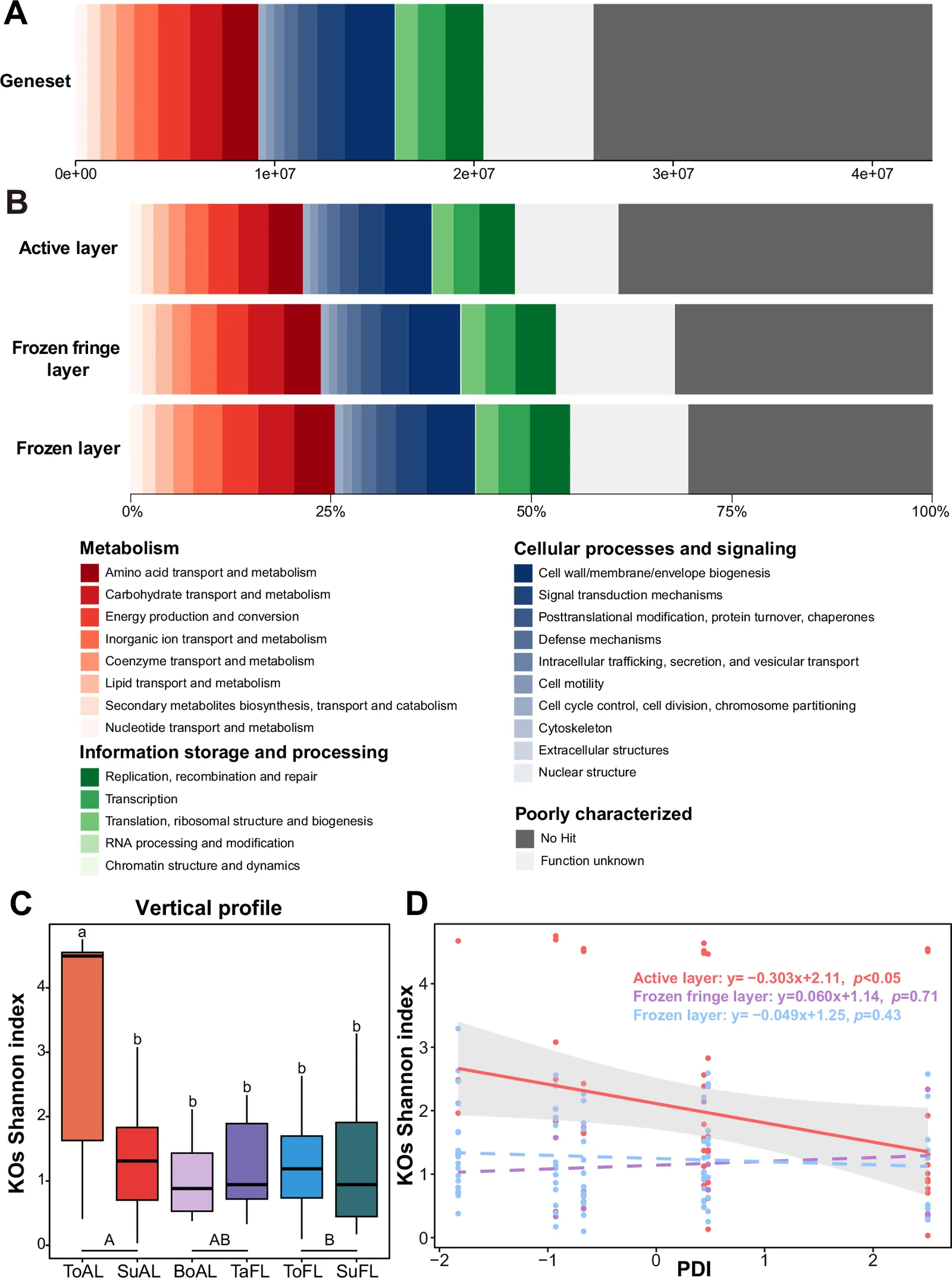
Permafrost microbiome functional potential. **A** Distribution of Clusters of Orthologous Groups (COGs) functional annotations. **B** Distribution of COGs in the active, frozen fringe, and frozen layers, respectively. **C** Distribution of Shannon index of Kyoto Encyclopedia of Genes and Genomes (KEGG) Orthologs (KOs) across the main- and sub-layers (Active layer, *n* = 55, including ToAL, *n* = 18, and SuAL, *n* = 37; Frozen fringe layer, *n* = 12, including BoAL, *n* = 6, and TaFL, *n* = 6; Frozen layer, *n* = 83, including ToFL, *n* = 59, and SuFL, *n* = 24). Statistical significance is based on two-sided Kruskal–Wallis tests; lowercase letters indicate the significance of differences among the sub-layers (*p* < 0.05), while uppercase letters denote the significance of differences across the main-layers (*p* < 0.05). In the box plots, the central line indicates the median. The bounds of the box correspond to the 25th and 75th percentiles (interquartile range, IQR). The lower and upper whiskers extend to the minima and maxima within 1.5 × IQR, respectively. **D** Linear relationships between PDI and Shannon index of KOs across the main-layers. Solid lines indicate significant fitted ordinary least-squares regression models, with gray shaded areas denoting the 95% confidence intervals. Dashed lines indicate non-significant fitted ordinary least-squares regression models. PDI permafrost degradation index, ToAL top-active layer, SuAL sub-active layer, BoAL bottom-active layer, TaFL table-frozen layer, ToFL top-frozen layer, SuFL sub-frozen layer. Source data are provided as a Source Data file.

**Fig. 5:**
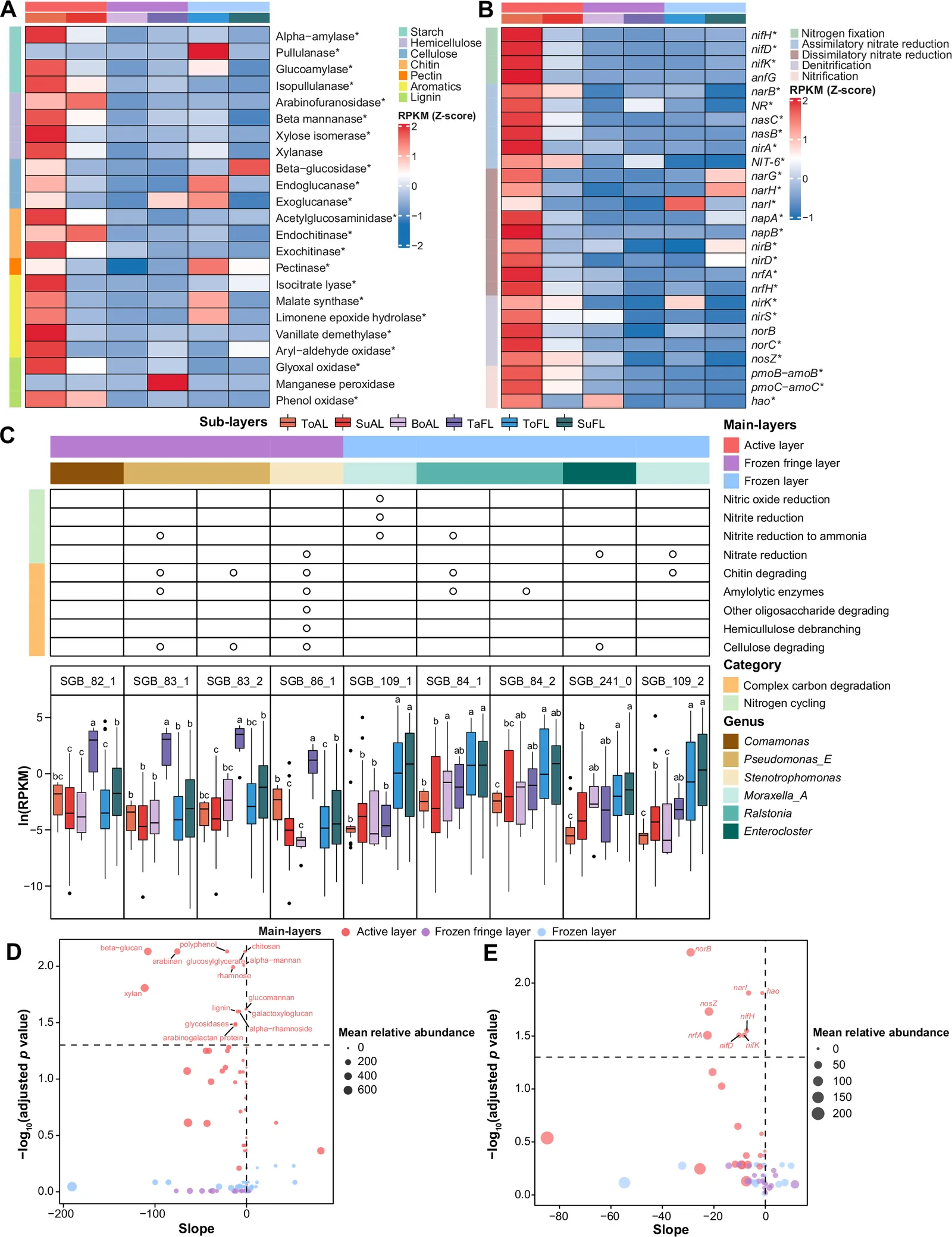
Variation of carbon/nitrogen cycling genes across the vertical profile and along the degradation gradient. The heatmap shows the enrichment of functional genes involved in carbon degradation (**A**) and nitrogen cycling (**B**) across the vertical profile. **C** Differential enrichment of species-level genome bins (SGBs) and their potential roles in carbohydrate and nitrogen metabolism in the frozen fringe and frozen layers and sub-layers (Active layer, *n* = 55, including ToAL, *n* = 18, and SuAL, *n* = 37; Frozen fringe layer, *n* = 12, including BoAL, *n* = 6, and TaFL, *n* = 6; Frozen layer, *n* = 83, including ToFL, *n* = 59, and SuFL, *n* = 24). In the box plots, the central line indicates the median. The bounds of the box correspond to the 25th and 75th percentiles (interquartile range, IQR). The lower and upper whiskers extend to the minima and maxima within 1.5 × IQR, respectively. Points beyond the whiskers represent outliers. **D**, **E** depict the correlations between the relative abundance of functional genes involved in carbon degradation, nitrogen cycling, and PDI in different main-layers, respectively. Statistical significance is based on two-sided Kruskal–Wallis tests; Lowercase letters represent the significance of differences across sub-layers. *P* values for the relationships between the relative abundance of each functional gene and PDI were obtained from linear models and adjusted using the Benjamini–Hochberg method. Slopes are the coefficients of the linear model. PDI permafrost degradation index, ToAL top-active layer, SuAL sub-active layer, BoAL bottom-active layer, TaFL table-frozen layer, ToFL top-frozen layer, SuFL sub-frozen layer, RPKM reads per kilobase per million mapped reads. Source data are provided as a Source Data file.

To elucidate the functional potential of permafrost microbiomes, we generated a catalog comprising 43 million non-redundant protein-encoding genes (90% identity threshold) and subsequently identified the genes by referring to the evolutionary genealogy of genes: Non-supervised Orthologous Groups (eggNOG; v5)^48^, Clusters of Orthologous Groups of proteins (COGs)^49^, and Kyoto Encyclopedia of Genes and Genomes (KEGG)^50^ databases (Fig. 4A). 39.53% of the encoded proteins showed no matches to existing databases, with an additional 12.84% representing uncharacterized COGs. This highlights the extensive and unexplored microbial functional potential in alpine permafrost. Among the annotated genes, those associated with amino acid transport and metabolism, cell wall/membrane/envelope biogenesis, replication, recombination, and repair processes were particularly abundant (Fig. 4A). A higher proportion of recovered genes from the deeper layers were associated with primary and secondary metabolism, while the percentage of unannotated and unclassified genes was greater in the active layer (Fig. 4B). Functional diversity, represented by the Shannon index of KEGG Orthologs (KOs), was substantially greater in the active layer than in the frozen fringe and frozen layers (Fig. 4C). Tp had the largest effect on KOs Shannon index across the vertical profile, accounting for 63% of the explained variation, followed by PWC (Supplementary Fig. 5A). Most genes related to carbon/nitrogen cycling were enriched in the active layer and markedly reduced along the vertical profile (Fig. 5A, B). Carbohydrate-active enzymes (CAZymes) demonstrated the highest abundance in the active layer (top-active layer) and declined down the profile (Supplementary Fig. 6 and Supplementary Data 7). In addition, all identified ARGs were enriched exclusively in the top-active layer (Supplementary Fig. 7A and Supplementary Data 8). We also found that VFs associated with various categories (e.g., stress survival, adherence, and exotoxins) were predominantly enriched in the top-active layer, while those related to adhesion, biofilm formation, and immune regulation were enriched in the table-frozen layer (Supplementary Fig. 7B and Supplementary Data 9).

Analysis of SGBs revealed distinct functional adaptations across the vertical profile. While most SGBs were enriched in the active layer (Fig. 2A), nine SGBs presented specific associations within sub-layers of the frozen fringe and frozen layers, eight of which were affiliated with the phylum of Proteobacteria (Supplementary Data 3). In particular, SGB_82_1 (*Comamonas* sp.), SGB_83_1 (*Pseudomonas_E* sp.), SGB_83_2 (*Pseudomonas_E* sp.), and SGB_86_1 (*Stenotrophomonas* sp.) were enriched in the table-frozen layer (Fig. 5C). These SGBs harbored genes involved in complex carbon degradation and nitrogen cycling. Specifically, SGB_86_1 presented the most comprehensive metabolic potential for the degradation of carbohydrates (e.g., cellulose and hemicellulose), whereas SGB_109_1 (*Moraxella_A* sp.) harbored genes involved in the nitrogen cycle (e.g., nitrate reduction, nitric oxide reduction, and nitrite reduction to ammonia) (Fig. 5C).

### Response of microbial community structure to permafrost degradation

Most MAGs recovered from the deeper layers (16 out of 23) clustered into the SGBs assigned to Proteobacteria, and the proportion of these MAGs displayed a decreasing trend along the degradation gradient from DC1 to DC6 (Fig. 2A and Supplementary Data 10). Across the entire vertical profile, only the prokaryotic community composition exhibited pronounced variations along the degradation, with pH and PWC showing the higher correlations. In addition, the composition of prokaryotic and viral communities specifically in the active layer varied markedly as permafrost degraded, with redox potential (Eh) emerging as the dominant influencing factor (Supplementary Figs. 8 and 9). The Shannon index of SGBs was negatively correlated with PDI only in the active layer (Fig. 2C). PWC positively influenced the Shannon index of the SGBs in the active layer, whereas Tp exhibited negative effect in response to the degradation (Supplementary Fig. 10A). Similarly, viral Shannon index in the active layer significantly declined with increasing PDI (Fig. 3D). In contrast to the suppressive effects of pH and Tp, Eh and PWC supported higher viral Shannon index in the active layer (Supplementary Fig. 10B). Most notably, we found that the relative abundance of *Lavidaviridae* (virophages) significantly increased with PDI in the frozen fringe and frozen layers (Supplementary Fig. 11). It suggests that virophages proliferate in response to changes in their giant virus hosts with the degradation, potentially influencing the diversity of nucleocytoplasmic large DNA viruses^51^.

### Variations in functional potential of permafrost microbiomes with the degradation

The Shannon index of KOs in the active layer was negatively correlated with PDI, with Tp exerting the strongest negative influence but PWC showing the greatest positive effect (Fig. 4D and Supplementary Fig. 5B). Similarly, relative abundances of those genes related to carbon/nitrogen cycling in the active layer also markedly decreased along the degradation gradient (Fig. 5D, E). The Shannon index of CAZymes in the active layer was negatively correlated with PDI (Supplementary Fig. 12), primarily driven by genes linked to the degradation of beta-glucan, xylan, and arabinan (Fig. 5B). Furthermore, the Shannon index of ARGs, rather than that of VFs, markedly decreased in the active layer with the degradation, whereas both ARGs and VFs exhibited a pronounced reduction in richness (Supplementary Fig. 13A–D). However, none of these gene categories showed a significant correlation with PDI across the vertical profile (Supplementary Fig. 13E and F).

**Fig. 6:**
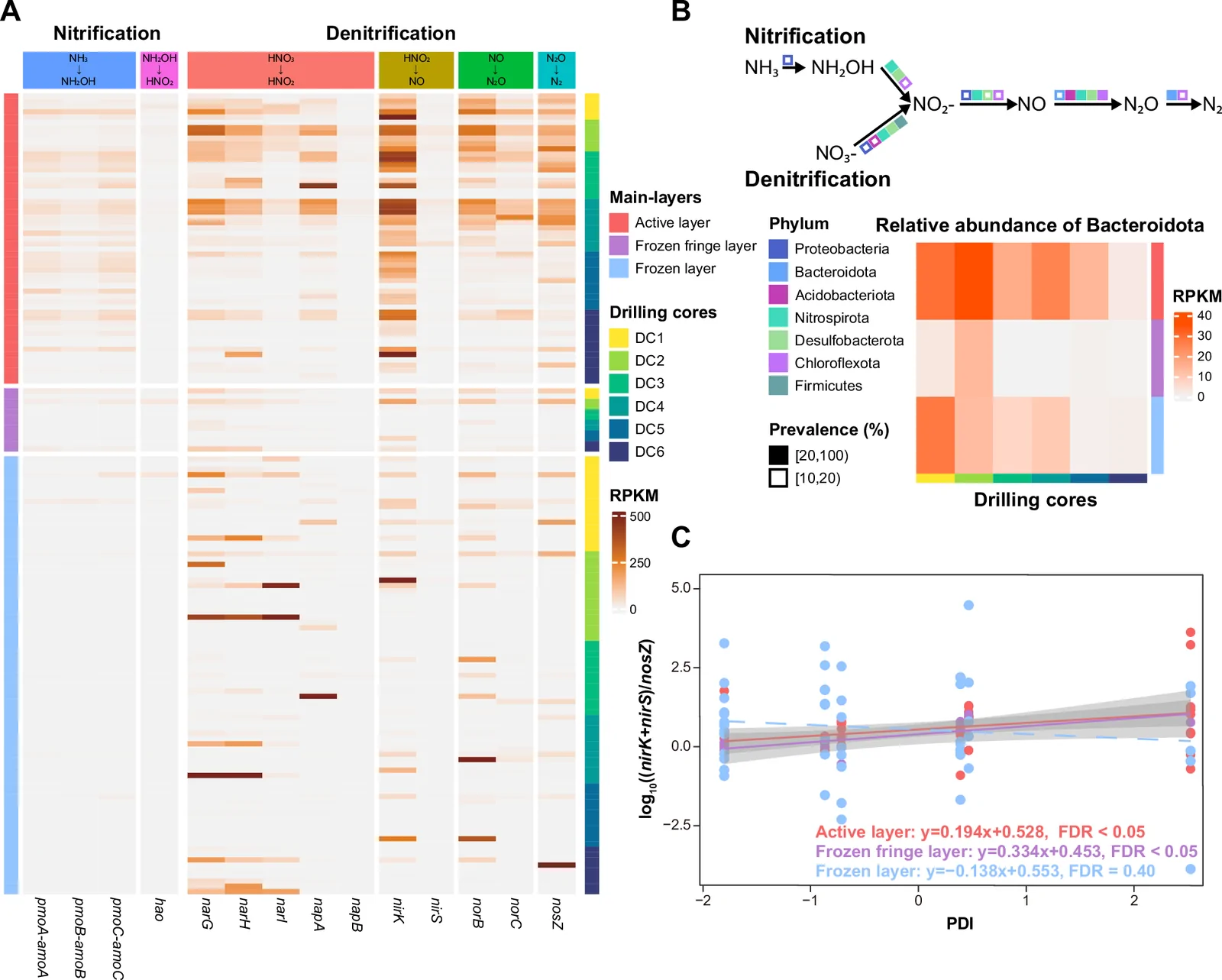
Association between the microbial metabolic potential related to nitrous oxide (N_2_O) emission across the vertical profile and along the degradation gradient. **A** Relative abundance of nitrification- and denitrification-associated functional genes across the main-layers and drilling cores. **B** Schematic diagram shows phyla related to N_2_O production and prevalence of the relevant protein-encoding genes in each phylum, as well as the relative abundance of Bacteroidota across the main-layers and drilling cores. **C** Association between the nitrite reductase/nitrous oxide reductase ratio (*nirK* + *nirS*)/*nosZ* and PDI across the main-layers. *P* values were adjusted using the Benjamini–Hochberg method to control the false discovery rate (FDR). Solid lines indicate significant fitted ordinary least-squares regression models, with gray shaded areas denoting the 95% confidence intervals. Dashed lines indicate non-significant fitted ordinary least-squares regression models. PDI permafrost degradation index, RPKM reads per kilobase per million mapped reads. Source data are provided as a Source Data file.

To further assess the ecological importance of permafrost microbial metabolic pathways, we analyzed genes involved in nitrification and denitrification, which are directly linked to nitrous oxide (N_2_O) production^52^. Numerous genes participating in these pathways presented relatively high incidences and relative abundances mainly in the active layer (Fig. 6A). Genes responsible for the conversion of nitrate to nitrite (NO_3_^-^ to NO_2_^-^), nitrite to nitric oxide (NO_2_^-^ to NO), and nitric oxide to N_2_O (NO to N_2_O) were also present in the frozen layer and along the degradation gradient (Fig. 6A). Meanwhile, we found that genes associated with N_2_O biosynthesis occurred across diverse prokaryotic taxa, whereas those linked to N_2_O reduction were presented at significant levels only in MAGs attributed to Bacteroidota and Chloroflexota (Fig. 6B). In particular, the relative abundance of Nitrospirota, which exhibited a high prevalence of genes related to N_2_O biosynthesis, did not significantly decrease with increasing PDI. In contrast, Bacteroidota experienced a drastic decrease in relative abundance, especially in the active and frozen layers (Fig. 6B and Supplementary Fig. 14). The ammonia-oxidizing archaeon *Nitrososphaera* sp. (SGB_227_2) in the active layer showed a positive association with PDI (Supplementary Fig. 15). To assess the genetic potential related to N_2_O emission, we investigated the ratio of nitrite reductase (*nirK* + *nirS*) to N_2_O reductase (*nosZ*), a proxy widely used to indicate potential N_2_O production^52,53^. The positive correlations were observed between PDI and (*nirK* + *nirS*)/*nosZ* in the active and frozen fringe layers, indicating that permafrost degradation would likely result in an increase in N_2_O emissions from these layers (Fig. 6C).

### Biotic and abiotic regulation of carbon/nitrogen loss under permafrost degradation

We quantified permafrost carbon and nitrogen stocks as indicated by permafrost organic carbon density (POCD) and permafrost total nitrogen density (PTND), respectively. Along the degradation gradient, POCD markedly declined throughout the entire vertical profile, while significant reductions in both POCD and PTND were confined to the active layer (Supplementary Fig. 16 and Supplementary Data 11). The random forest model explained 70% of the variance in POCD and highlighted hydrothermal regime and the Shannon indices of microbiomes as the primary controls on POCD variation across the vertical profile (Supplementary Fig. 17A). For the active layer, the models for POCD and PTND explained 62% and 53% of the variance, identifying PWC and KOs Shannon index as their primary determinants, respectively (Supplementary Figs. 18A and 19A). To evaluate the effects of hydrothermal regimes, chemical properties and microbiomes on POCD and PTND during permafrost degradation, we constructed structural equation models (SEMs; all *P* > 0.05, with a root mean square error of approximation [RMSEA] of 0.00 and a comparative fit index [CFI] of 1.00 for all models) (Supplementary Figs. 17B, 18B, 19B, and Supplementary Data 12), uncovering clear pathways of biotic and abiotic factors regulating carbon/nitrogen stock. For the entire vertical profile, the model accounted for 62% of the variance in POCD. Specifically, increasing PDI significantly elevated Tp but reduced PWC, which subsequently altered the Shannon index and composition of permafrost microbiomes (Supplementary Fig. 17B). Moreover, PDI indirectly influenced KOs Shannon index through its opposite effects on Tp and PWC. Interestingly, the degradation-affected microbiomes showed the strongest associations with POCD, emphasizing their important role in regulating carbon stock with the degradation (Supplementary Fig. 17C). In the active layer, the SEMs interpreted 77% of the variance in POCD and 69% in PTND (Supplementary Fig. 18B, C). Likewise, PDI exerted a positive correlation with Tp but a negative correlation with PWC, thereby influencing microbial community structure and KOs Shannon index. Distinct drivers were observed, with POCD mainly constrained by hydrothermal regime and PTND primarily mediated by microbiomes. Collectively, permafrost degradation altered hydrothermal regimes and microbiomes, ultimately leading to carbon/nitrogen loss (Supplementary Figs. 17C, 18C, and 19C).

## Discussion

This study establishes a comprehensive dataset of microbiomes across the vertical profile in the QTP. We obtained six 15 m-depth permafrost cores, which yielded 150 metagenomes, 501 MAGs, 43 million non-redundant genes, and 28,008 viral genomes, thereby establishing a foundational genomic resource for alpine permafrost. Notably, we found that 265 SGBs were previously undescribed species among the identified 282 SGBs, highlighting the remarkable uniqueness and richness, as well as the important potential functions of microorganisms in QTP permafrost.

Our results indicated that the active layer of alpine permafrost was dominated by Bacteroidota and Thermoproteota. Proteobacteria occurred across all layers and increased in relative abundance with profile depth. These taxonomic variation patterns differ from those reported in previous studies. For example, Actinobacteria dominated the active layer in Arctic permafrost regions, whereas Proteobacteria primarily occurred in the surface-frozen layer of alpine permafrost in the QTP^29,37^. In addition, Patescibacteria and Nitrospirota were detected in our study, which had not been previously observed in other permafrost regions like the Arctic and QTP^27,54^. Such differences indicate that high-altitude permafrost hosts distinct microorganisms, highlighting potential regional heterogeneity. A pronounced decrease in the recovered microbial and functional diversities was observed from the active layer through the frozen fringe layer to the frozen layer, consistent with variations mentioned in other permafrost regions^29,55^. Increasing profile depth adds multiple stresses to microbial communities including reduced resource availability, lowered temperature, and limited diffusion^21,56^. Specifically, low temperatures reduce membrane fluidity and restrict the diffusion of metabolic substrates, thereby suppressing cell division and enzyme activity^57^. Meanwhile, the higher pH values typically found in deeper layers (frozen fringe and frozen layers) may alter the adsorption-desorption balance of key elements, aggravate nutrient limitation^56,58^ and ultimately contribute to the lower diversity observed in the deeper layers^29,59^. Microorganisms in this extreme environment encode genes for complex carbon degradation^30,60^, indicating a shift towards utilization of less energetically favorable substrates due to limited access to labile carbon^29,56^. For example, the enrichment of complex carbon degradation genes in specific taxa within deeper layers (e.g., SGB_86_1, *Stenotrophomonas* sp.) suggests that these taxa may act as keystone species, breaking down polymers into smaller compounds that other microorganisms can utilize^61^. Concurrently, nitrite reduction genes were also enriched in the frozen layer (e.g., SGB_109_1, *Moraxella_A* sp.), suggesting a heightened demand for nitrogen, potentially due to increased nitrogen limitation^62^ and intensified microbial competition for nitrogen resources^63^. Multiple evidence of our results revealed that hydrothermal regimes had the strongest effect on microbial community structure and functional potential across the permafrost profile, reflecting the central role of temperature and water content in controlling metabolic rates, substrate diffusion, and microbial connectivity^64,65^.

Permafrost degradation drove shifts in microbial community composition but did not follow a single, simple directional pattern. A more consistent pattern was the pronounced losses of microbial and functional diversity in the active layer. We found hydrothermal regimes to be the most important factors underlying these variation patterns. Previous studies have shown rapid shifts in community composition with fast-growing taxa becoming more prevalent as permafrost transitions from the frozen to thawed states^33,66^. In contrast, the rising temperature generally constrains the growth and metabolic ability of cold-adapted microorganisms by altering their physiological activity and impairing cellular functions^14,40^, helping to explain decrease in much of microbial diversity observed in the active layer under the degradation. Interestingly, a significant decrease in CAZyme diversity in the active layer was observed with the degradation. This result was opposite to some previous findings^30,67^. It may be because degradation-induced shifts in hydrothermal regimes not only release more readily available substrates but also impose stronger environmental filtering on microorganisms with genomic potential for complex carbon degradation^68^. These changes favor taxa that rely more on labile carbon and reduce the dominance of taxa carrying more diverse CAZyme repertoires^69^. Together, these mechanisms may contribute to the observed decline in CAZyme diversity but warrant further validation through direct substrate characterization. Meanwhile, significant declines were observed in POCD and PTND in the active layer with the degradation, in accordance with other findings^14,70^. Based on linear regression models, each 10% increase in PDI relative to its observed range was associated with estimated declines of 5.5% in POCD and 5.9% in PTND. This may be attributed to the fact that microbial respiration responds more sensitively to temperature than growth, and that permafrost warming potentially shifts carbon use efficiency and accelerates through heterotrophic respiration^71^. Simultaneously, shifts in community composition break the balance between N_2_O production and reduction. The abundance of Bacteroidota decreased as permafrost degraded, which was coupled with stable abundance of N_2_O-producing Nitrospirota and significantly increased abundance of *Nitrososphaera* sp. (SGB_227_2), suggesting a potential shift toward greater N_2_O production potential. Besides, as a key genomic proxy of N_2_O production potential^52,53^, (*nirK* + *nirS*)/*nosZ* in the active and frozen fringe layers increased distinctly with the degradation. These findings indicated a heightened genetic capacity for nitrogen turnover, aligning with previous studies that associate thawing permafrost with increased N_2_O emission potential^39,52,56^. Overall, our results suggest that permafrost degradation profoundly influences both abiotic and biotic factors and is likely associated with declining carbon and nitrogen stocks. However, while the increased (*nirK* + *nirS*)/*nosZ* ratio indicates an enhanced metabolic potential for N_2_O emission, actual metabolic rate and in situ emission remain unconfirmed. Further studies should incorporate multiple methods, including metatranscriptomic sequencing and flux measurement, to further validate this pattern.

Notably, about 74% of the recovered viral genomes remained unclassified at the family level, indicating that a high proportion of viral lineages in alpine permafrost remains unresolved under current reference frameworks. This pattern likely reflects both the true novelty of viral lineages in alpine permafrost and the limitations arising from the incompleteness of current reference databases. Viruses from permafrost remain underexplored, and currently available databases and classification tools often have limited ability to assign highly divergent viral sequences^72^. Consistent with this interpretation, previous studies of Arctic permafrost also reported similarly high proportions of unclassified viral genomes (83% and 85%), indicating that a large unknown fraction of viruses is common in permafrost^43,44^. The enrichment of *Pithoviridae* alongside *Lavidaviridae* (virophages) in the frozen fringe layer could have several important implications. For instance, virophages are known to infect giant viruses and suppress their replication within eukaryotic hosts, thereby leading to host population turnover and altering community composition^73,74^. However, it is worth emphasizing that our metagenomic data support only the detection of DNA viruses. In addition, interactions among virophages, giant viruses, and their hosts can mediate horizontal gene transfer and modulate host metabolism^75^, with potential consequences for carbon/nitrogen cycling in degraded permafrost^43,44^. Likewise, VFs related to mobile genetic elements were highly enriched in the frozen fringe layer, suggesting a latent reservoir of available pathogenic factors that could potentially be mobilized during permafrost degradation^76,77^. Mobile elements may facilitate horizontal gene transfer among microorganisms, enabling the rapid dissemination of virulence traits^78^. To be more specific, when the frozen fringe layer transforms into the active layer, enhanced microbial activity and physical transport processes could increase the rates of conjugation, transduction and transformation, further accelerating the spread of VFs^79,80^. Moreover, the detected ARGs in permafrost also raised concerns that degradation-induced release of ARGs might facilitate co-selection of resistance and virulence traits in recipient communities^76,81,82^. While our dataset characterizes the genomic reservoir of viruses, VFs, and ARGs, it does not provide direct evidence of mobility, viability, or activity, nor does it establish direct links to known pathogens or resolve their genomic context. In the future, it is essential to combine with RNA viruses due to higher mutation rates and greater potential risks for comprehensive assessments of potential biosafety risks in permafrost. Incorporating metatranscriptomics and long-read metagenomic sequencing in future studies will also be important. Future investigations covering the entire QTP, together with comparisons with contemporary environmental communities, are pivotal not only for advancing our understanding of alpine permafrost viromes but also for comprehensively assessing potential biosecurity concerns under permafrost thawing.

It is also important to acknowledge a limitation inherent in our genome-resolved analyses. The majority of the recovered MAGs and viral genomes originated from the active layer, with substantially fewer genomes reconstructed from the deeper layers. In addition, despite the large amount of raw data generated for each sample, only a relatively small fraction of reads was incorporated into assembled contigs, and assembly output was quite different among samples. We believe these disparities are primarily driven by intrinsic biological constraints of some samples, especially low-biomass samples from the deeper layers. In these samples, low recoverable DNA and limited non-redundant sequences may reduce retention of clean reads and assembly efficiency, thereby resulting in fewer or shorter contigs^21,83^. In addition, the potential DNA damage and fragmentation may have also constrained assembly continuity^84^. Consequently, both our SGB catalog and viral genome catalog, the reference databases used for diversity comparisons, are inherently biased toward the active layer. Prokaryotic taxa and viral lineages uniquely adapted to the deeper layers, particularly those possessing unusual genomic features that resist assembly and present at extremely low abundances, may be partially missing from our catalogs or not represented because the recovered genomes fail to meet our quality thresholds. Nonetheless, our abundance profiling provides a more quantitative perspective for the recovered prokaryotes and viruses, based on read recruitment to the dereplicated SGBs and viral operational taxonomic units (vOTUs). Furthermore, the consistent depth-related declines in both SGBs, viral and functional diversity (Figs. 2B, 3C, and 4C), along with their significant correlations with measured environmental variables (e.g., hydrothermal regimes), lend strong support to the biological authenticity of these vertical stratification patterns.

In summary, our study systematically elucidated the vertical stratification patterns of microbiomes and their response to permafrost degradation in the QTP, establishing a comprehensive genomic dataset encompassing MAGs, viral genomes, and non-redundant protein encoding gene catalog from 15 m-depth alpine permafrost. The findings demonstrated that microbiome diversity consistently declined across key profile layers, and microbiomes in the active layer were considerably more sensitive to permafrost degradation, showing pronounced losses in microbial and functional diversity. Hydrothermal regimes primarily regulated these variation patterns, leading to carbon/nitrogen loss during the degradation. In contrast, deeper frozen layers maintain relatively stable microbial communities but harbor extensive genetic reservoirs including ARGs, VFs, and diverse viruses that could pose latent biosecurity risks after degradation. In the future, it is imperative to establish a long-term and large-scale integrated monitoring network encompassing both high-altitude permafrost on the QTP and high-latitude permafrost in the Arctic to comprehensively understand the spatiotemporal patterns of microbial community structure and functional potential in permafrost regions, as well as the ecological consequences and potential biohazards arising from progressive degradation of permafrost under global warming.

## Methods

### Study area

The study area is located in the Shule River Headwaters (38.0 to 39.0° N, 97.3 to 99.0° E, and 3402 to 5,814 m above sea level) on the western part of the Qilian Mountains, northeast margin of the QTP, China. This area belongs to the continental arid desert climate, with mean annual air temperature of ‒0.96 to ‒5.62 °C and precipitation of 201 to 532 mm based on the monitoring meteorological data. The dominant vegetation type is alpine grassland including alpine marsh meadow, alpine meadow, and alpine steppe^85^. Alpine permafrost is distributed extensively and occupies about 97.99% of the area^86^, with the ALT ranging from 1.2 to 6.0 m and ground temperature at a depth of 15 m ranging from ‒0.05 to ‒0.75 °C on the basis of the monitoring data.

### Collection of field samples and analysis of environmental variables

We collected permafrost samples of 15 m-depth drilling cores (DC1–DC6) from six different sites (S1–S6) when the permafrost reached the annual maximum thawing depth in October 2020, corresponding to the ALT (Fig. 1). A vibratory coring machine with stainless-steel tubes was used to obtain the permafrost samples. To minimize contamination, no water or drilling fluids were injected during the coring, and the outer 1 cm of each core was aseptically removed using sterilized scalpels. During field sampling, samples from each drilling core were collected every 10 cm, yielding 150 samples per core and 900 samples in total. The samples were divided into the following three parts: 1) samples designated for metagenomic sequencing were immediately transferred to cryotubes, preserved in liquid nitrogen during transport, and stored at ‒80 °C in the laboratory; 2) samples for measurements of PWC and soil bulk density (BD) were obtained by using a cutting ring with a standard volume of 100 cm³; 3) samples for analyses of Eh and pH were collected and maintained at 4 °C, whereas those for determination of permafrost organic carbon (POC) and total nitrogen (TN) were air-dried naturally.

The Hydra Probe II soil sensors (Stevens) and the temperature chains equipped with thermistors developed by the State Key Laboratory of Cryospheric Science and Frozen Soil Engineering were connected to the CR1000X datalogger (Campbell Scientific), automatically monitoring Tp at the corresponding drilling site from 0.1 to 15 m depths in each borehole and recording data at 10 min intervals. To address missing or erroneous data of Tp, we implemented machine learning algorithms using the scikit-learn library in Python (v3.12). First, a Savitzky–Golay filter (with a window length of 5 days) was used to remove erroneous data caused by instrument noise and sensor errors. Second, we employed Random Forest and Bayesian ridge regression models to interpolate missing data based on the complete data from adjacent depths or observational sites, and selected the optimal model based on its performance^87^. Mean *R*² and root mean square error of the models used for final data reconstruction were 0.97 and 0.15, respectively. The ALT of each drilling site was confirmed on the basis of Tp data^88^. Subsequently, we observed ALT and MAGT at a depth of 15 m among six drilling sites, respectively. These two key parameters reflecting permafrost stability were analyzed using PCA, yielding the first principal component represented as the PDI^46^ (Supplementary Fig. 1). Finally, permafrost degradation indices for six drilling sites were ranked in ascending order to characterize a degradation sequence of permafrost from S1 to S6.

Based on the ALT of each permafrost drilling site, the corresponding drilling core was systematically divided into six sub-layers including top-active layer (ToAL; 0~0.5 m depths); sub-active layer (SuAL; 0.5 m depth ~ above the bottom-active layer), bottom-active layer (BoAL; 10 cm depth above the junction of the active and frozen layers), table-frozen layer (TaFL; 10 cm depth below the junction of the active and frozen layers), top-frozen layer (ToFL; below the table-frozen layer ~ 12 m depth), and sub-frozen layer (SuFL; 12~15 m depths). Moreover, these sub-layers were categorized into three main-layers of permafrost stratigraphic structure containing the active layer from top-active layer to sub-active layer, the frozen fringe layer from bottom-active layer to table-frozen layer, and the frozen layer from top-frozen layer to sub-frozen layer. We then selected 25 representative samples from the aforementioned layers of each drilling core, resulting in 150 samples which were used for metagenomic sequencing and physicochemical analyses (Supplementary Data 13). POC and TN were quantified using the Walkley–Black dichromate oxidation method and the micro-Kjeldahl procedure, respectively. The calculations of POCD and PTND were performed using the following equations: ${{POCD}=\sum }_{i=1}^{n}{h}_{i}{{BD}}_{i}{{POC}}_{i}(1-{C}_{i})/100$ and ${{PTND}=\sum }_{i=1}^{n}{h}_{i}{{BD}}_{i}{{TN}}_{i}(1-{C}_{i})/100$. In these equations, hᵢ denotes the thickness of soil layer i (cm), BDᵢ denotes bulk density (g · cm^−3^), POCᵢ and TNᵢ denote the permafrost organic carbon and total nitrogen contents (g · kg^−1^), respectively, and Cᵢ denotes the volume percentage of coarse fragments >2 mm in layer i^14^. pH and Eh were measured using a PHBJ-260 pH meter (INESA) with a 1:5 soil-to-water ratio. The measurement of the PWC was based on the mass variations of fresh samples after drying at 105 °C for 24 hours.

### DNA extraction, library preparation, and sequencing

Approximately 3 g of each permafrost sample was used for DNA extraction. Samples were rinsed with phosphate-buffered saline, and the gravel was removed before extraction. DNA was extracted using a modified cetyltrimethylammonium bromide (CTAB) protocol for low-biomass permafrost samples (Chinese Patent No. ZL202210792689.0; Japanese Patent No. 7530612). Relative to a conventional CTAB procedure, the main modifications were the use of sodium dodecyl sulfate (SDS) plus proteinase K instead of radioimmunoprecipitation assay-type lysis and a longer incubation with preheated CTAB/NaCl. Specifically, sample suspension after homogenization using a tissue homogenizer was centrifuged at 13,000 × g for 5 minutes, followed by discarding the supernatant. The remaining substance was resuspended in Tris-EDTA (TE) buffer. For cell lysis, 0.5 mL of 10% SDS and 50 μL of proteinase K (20 mg/mL) were added, and the mixture was incubated at 37 °C for 1 h. Subsequently, 1.5 mL of NaCl (5 mol/L) was added and mixed, followed by 1.5 mL of CTAB/NaCl solution preheated to 65 °C and incubation at 65 °C for 30 min. The lysate was extracted with phenol:chloroform:isopentanol (25:24:1) mixture, and the aqueous phase was used for DNA precipitation with isopropanol. The resulting DNA was purified using 1× AMPure XP beads, followed by whole-genome amplification (WGA) utilizing N6 primers (Beijing Liuhe BGI Co., Ltd.) and Phi29 DNA polymerase.

To monitor potential contamination during DNA extraction and WGA, negative controls were included and processed in parallel with the real samples. Following the amplification, all negative controls were tested by 16S rRNA gene PCR. All of them tested negative, indicating no detectable prokaryotic DNA contamination. Therefore, these negative controls were not subjected to metagenomic library preparation and sequencing.

Finally, shotgun metagenomic sequencing libraries were constructed using a previously established method, and sequencing was performed using the DNBSEQ-T10 (MGI, China) platform with a paired-end 100 bp (PE100) strategy; at least 50 GB of sequencing data was obtained from each sample.

### Metagenomic assembly and binning

Low-quality reads were filtered using fastp (v0.23.2)^89,90^. Reads that passed the quality control assessment were assembled using a single-sample assembly strategy with MEGAHIT (v1.2.9)^91^, retaining contigs exceeding 500 bp in size for subsequent analysis. Metagenomic binning was performed independently for each sample using MetaWRAP (v1.3)^92^. The contamination and completeness levels of the MAGs were evaluated using CheckM (v1.2.2)^93^ in lineage_wf mode, and only those MAGs meeting the criteria for medium quality as defined by MIMAG^47^ were retained for further investigation. To enable genome-resolved analyses at the SGB level, MAGs were clustered into SGBs using dRep (v3.4.2, parameters: -sa 0.95 -nc 0.30)^94^ at a 95% ANI threshold^95^. The representative genome selected from each cluster was defined as an SGB. The SGBs were taxonomically classified using Genome Taxonomy Database Toolkit (GTDB-Tk; v2.3.2, GTDB release 207)^96^. Phylogenetic trees of the SGBs were generated through multiple sequence alignment utilizing the GTDB-Tk infer and root module^96^. Visualization of the phylogenetic trees was accomplished using the R packages “ggtree”^97^ and “ggtreeExtra”^98^. To gain insights into the metabolic potential of the permafrost microorganisms, the MAGs were annotated for metabolic functions using METABOLIC (v4.0, default options)^99^.

### Construction and characterization of the gene catalog

The contigs obtained from the metagenomic assembly were utilized as input for gene prediction using Prodigal (v2.6.3)^100^. The predicted genes were clustered using the MMseqs2 (v14.7e284) easy-cluster pipeline, employing a threshold of 80% coverage and 90% identity. To functionally annotate the non-redundant genes, eggNOG-mapper (v2.1.11)^101^ was employed, utilizing the eggNOG database version 5.0.2^48^. The functional annotations derived from eggNOG-mapper included COG^49^, KEGG, and KO annotations^50^. Furthermore, the genes were searched against the Virulence Factor Database SetB^102^ database using DIAMOND (v2.1.8.162, in ‘blastp’ mode)^103,104^ with the parameters ‘-id 90 --query-cover 80 --max-target-seqs 1 --evalue 1e-6’. Additionally, the genes were queried against the Comprehensive Antibiotic Resistance Database (CARD)^105^ using Resistance Gene Identifier (https://github.com/arpcard/rgi, v6.0.2), and the CAZyme annotations were obtained using dbCAN3^106^.

### Generation and annotation of permafrost viral genomes

To conduct de novo virus prediction from the permafrost metagenome, all assembled contigs were processed using VirSorter2 (v2.2.3)^107^, and contigs achieving a score greater than 0.7 were retained for further analysis. The potential viral contigs were evaluated subsequently using CheckV (v1.0.1)^108^. We retained contigs encoding at least one virus-specific gene and longer than 2 kbp. These contigs were identified as viral genomes. The use of this length threshold can reduce noise from very short and highly fragmented contigs while still capturing a broad spectrum of viral sequences for dereplication and annotation. To facilitate genome dereplication, the viral genomes were clustered into vOTUs using CD-HIT (v4.8.1) with an identity threshold set at >95%. The identification of prokaryotic hosts for the viral genomes was accomplished through VirHostMatcher-Net, which employs default parameters, and results with prediction scores exceeding 0.8 were considered reliable. Taxonomic assignment of the viral genomes at the family level was conducted using Demovir (https://github.com/feargalr/Demovir).

### Metagenomic read mapping and abundance profiling

The clean metagenomic reads were aligned to the SGBs and vOTUs using bwa-mem (v0.7.17)^109,110^ with default parameters. Reads that exhibited an alignment identity greater than 95% and an alignment length exceeding 45 nt were retained for subsequent abundance estimation. The relative abundance of the SGBs and vOTUs was calculated on the basis of the alignments obtained using the reads per kilobase per million mapped reads (RPKM) mode of CoverM (https://github.com/wwood/CoverM, v0.6.1, options: -m rpkm --min-read-aligned-length 45 --min-read-percent-identity 95 in genome mode). Furthermore, translation mapping to the gene catalog was performed using DIAMOND (v2.1.8.162)^103,104^ in “blastx” mode with the options ‘-id 90 --eValue 1e-6 -k 1 --max-hsps 1’.

### Statistical analysis

All the statistical analyses were performed in R (v4.4.1). The environmental variables considered in this study include hydrothermal regimes (Tp and PWC) and chemical properties (pH and Eh). We performed the Kruskal–Wallis test to evaluate differences in permafrost environmental variables and microbiomes. The prokaryotic and viral community compositions were represented by the first axis of the PCA, and envfit analysis in the “vegan” package was used to analyze the relationship between microbial community composition and environmental variables^111^. The “rdacca.hp”^112^ and “glmm.hp”^113^ packages were used to construct linear mixed-effects models to quantify the impact of environmental variables on SGBs and viral Shannon index. Generalized linear models were used to assess the relative influence of environmental variables and PDI on diversity with the “h2o” package^114^. The relative importance of PDI, environmental variables, and microbiomes in explaining carbon/nitrogen stocks was assessed by random forest models using “rfPermute” and “randomForest” packages^115,116^. Considering that samples originated from multiple profile layers within cores from six drilling sites, a potential nesting effect was present in the data used for SEM construction. To evaluate the nesting effect, we first calculated intraclass correlation coefficients (ICC) using SPSS 27.0. The nesting effect (also known as the design effect, Deff) was calculated using the formula [Deff = 1 + (m − 1) × ICC], where m represents the average cluster size. In calculating Deff, negative ICC estimates were set to zero. For the sample data used in SEM1 construction (Supplementary Fig. 17B), m was 25, ICC was 0.005, and the resulting Deff was 1.120. For SEM2 (Supplementary Fig. 18B) and SEM3 (Supplementary Fig. 19B), *m* was 9.167, and ICC was negative (−0.113), and the corresponding Deff was 1.000. Obviously, all Deff values < 2 that indicate the ignorable nesting effect^117^. Each SEM was constructed by AMOS 24.0 after a priori hypothesis regarding the relationships among permafrost degradation parameter, environmental variables, microbiomes, and carbon/nitrogen stock. Finally, the model fit was evaluated using multiple indices, including the ratio of chi-square to degrees of freedom (*χ*²/df), the RMSEA, the CFI, and the *p*-value of the Bollen–Stine bootstrap test. The model was considered to be acceptable with 0 ≤ *χ²*/df ≤ 2, RMSEA < 0.05, CFI > 0.9, and *p* > 0.1. The resulting SEM diagrams were manually redrawn in Adobe Illustrator (version 29.5.1). Statistical data visualizations, including stacked bar charts, boxplots, scatterplots, donut plots, heatmaps, line plots, and bar plots, were generated in R (v4.4.1) using the “ggplot2” package^118^.

### Reporting summary

Further information on research design is available in the Nature Portfolio Reporting Summary linked to this article.

## Supplementary information

Supplementary Information

Description of Additional Supplementary Files

Supplementary Data 1-13

Reporting Summary

Transparent Peer Review file

## Source data

Source Data

## Data Availability

The sequencing data generated in this study have been deposited in the Genome Sequence Archive^119^ in National Genomics Data Center^120^, China National Center for Bioinformation/Beijing Institute of Genomics, Chinese Academy of Sciences with accession number CRA045482. The genome sequences for the SGBs used in the analyses presented in this study are publicly available in Figshare at (https://doi.org/10.6084/m9.figshare.32769480). The repository also contains an expanded non-redundant SGB collection generated using combined single-sample and co-assembly strategies. Source data are provided with this paper.
