# Supplementary material for: Hydrothermal regimes regulate microbiome patterns across vertical profile and their responses to alpine permafrost degradation
Source: Nat Commun. 2026 Sep 9;17:10636. doi: 10.1038/s41467-026-77448-6 (PMC13650142; doi:10.1038/s41467-026-77448-6)
Supplement: Supplementary file 1 [file 41467_2026_77448_MOESM1_ESM.pdf]

# Supplementary Information

## Supplementary figures

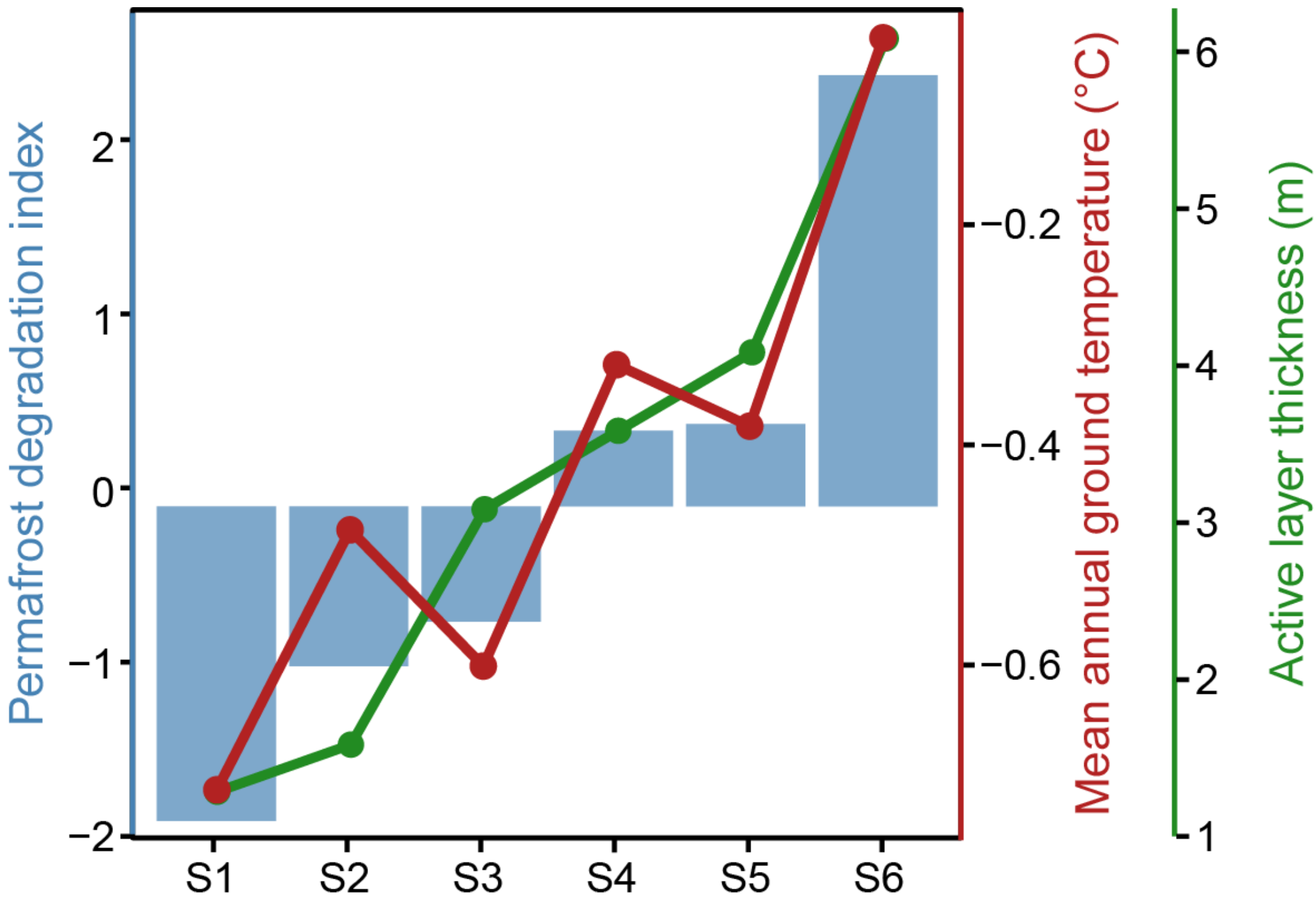


**Supplementary Fig. 1 | Permafrost degradation index, ground temperatures, and active layer thickness of six drilling sites.** The permafrost degradation index is the first principal component obtained from the active layer thickness and mean annual ground temperature at 15 m depth using principal component analysis and is used to rank the degrees of permafrost degradation of the six drilling sites, in ascending order from S1 to S6 to represent a degraded sequence.

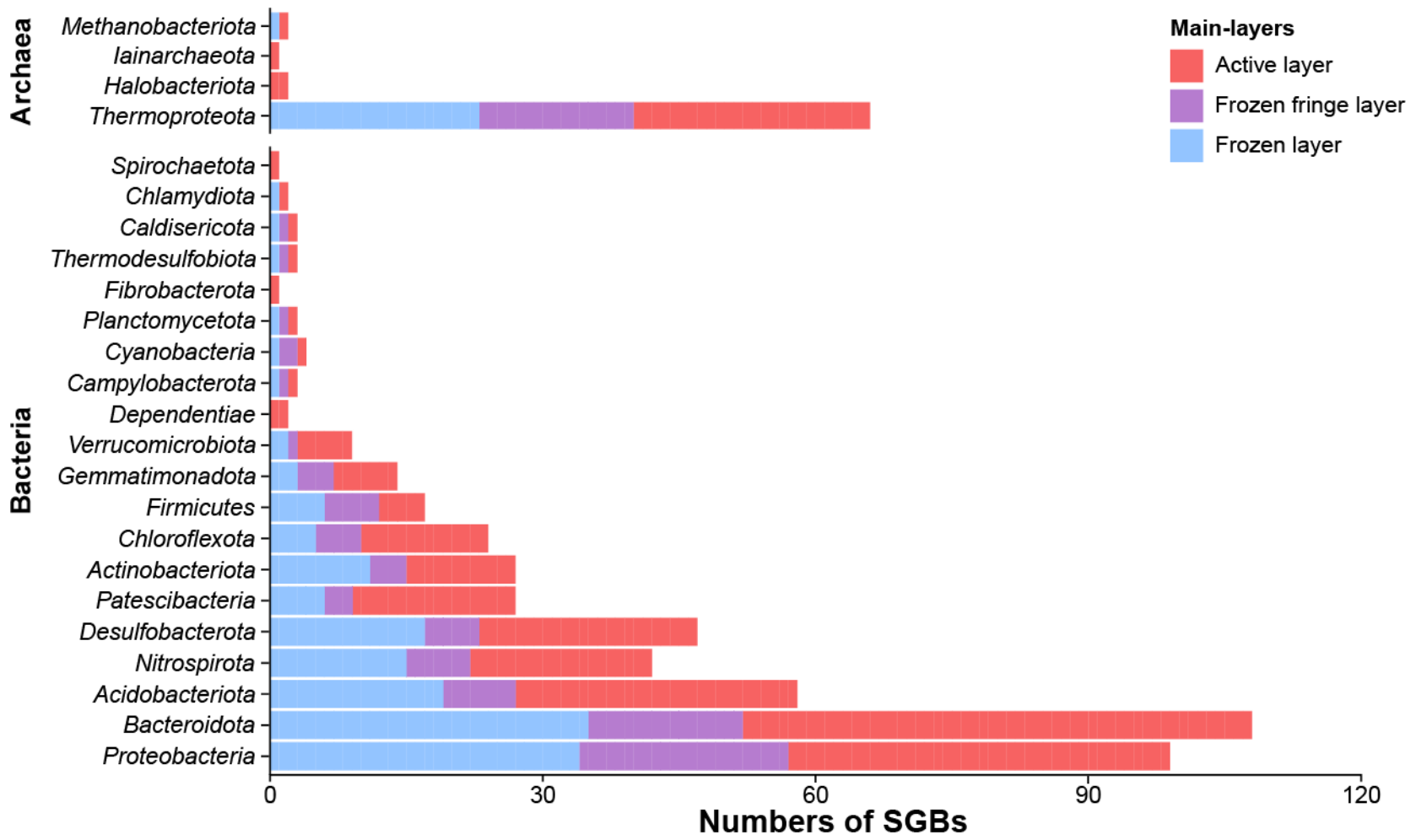


**Supplementary Fig. 2 | Distribution of species-level genome bins (SGBs) across the main-layers at the phylum level.** Horizontal stacked bars show the numbers of SGBs assigned to each archaeal or bacterial phylum in the active, frozen fringe and frozen layers, which are indicated in red, purple and blue, respectively. The x-axis represents the number of SGBs detected in each layer. An SGB occurring in more than one layer was counted separately in each corresponding layer.

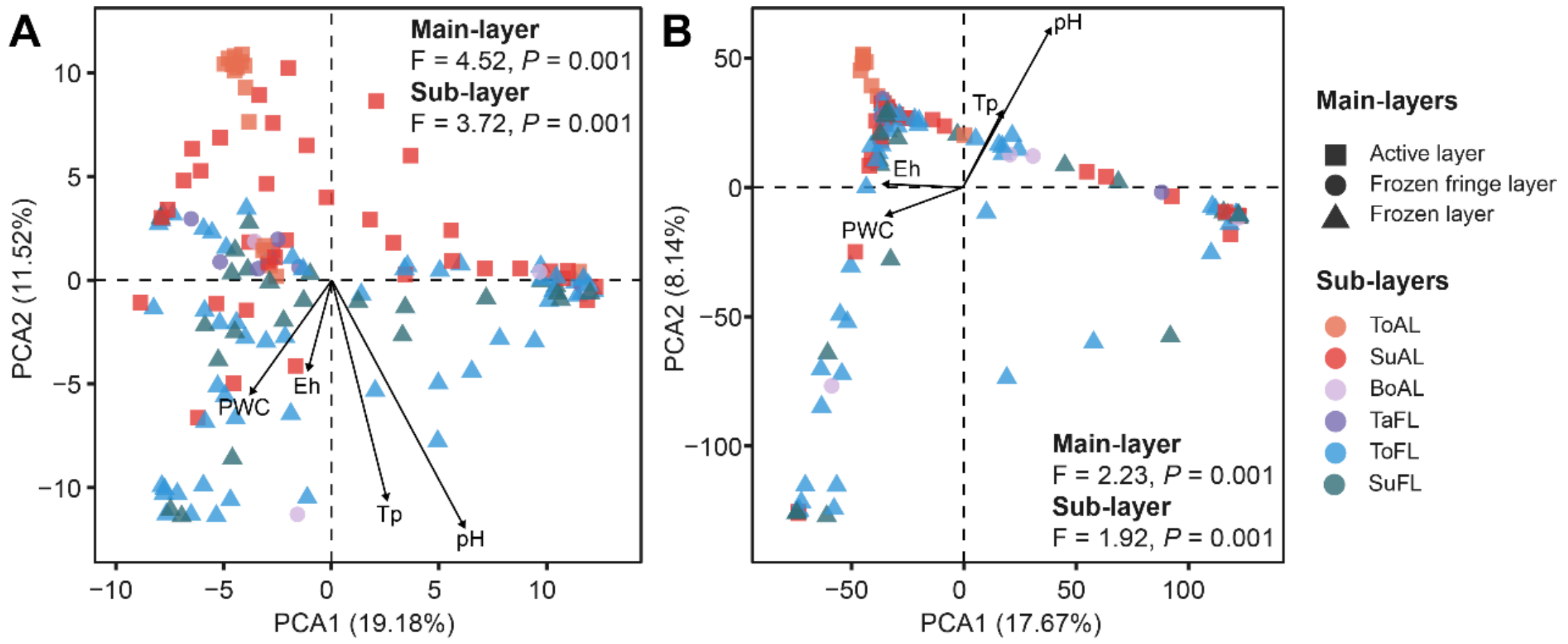


**Supplementary Fig. 3 | Variations and influencing factors of microbial community composition across vertical profile.** Principal component analysis (PCA) combined with environmental fitting (envfit) shows the associations between prokaryotic (**A**) and viral (**B**) community compositions and environmental variables (arrowed lines) across vertical profile. Sample sizes ($n$) are provided for each main-layer and their respective sub-layers: active layer ($n$ = 55; including ToAL, $n$ = 18; SuAL, $n$ = 37), frozen fringe layer ($n$ = 12; including BoAL, $n$ = 6; TaFL, $n$ = 6), and frozen layer ($n$ = 83; including ToFL, $n$ = 59; SuFL, $n$ = 24). Tp, permafrost temperature; PWC, permafrost water content; Eh, redox potential. ToAL, top-active layer; SuAL, sub-active layer; BoAL, bottom-active layer; TaFL, table-frozen layer; ToFL, top-frozen layer; SuFL, sub-frozen layer.

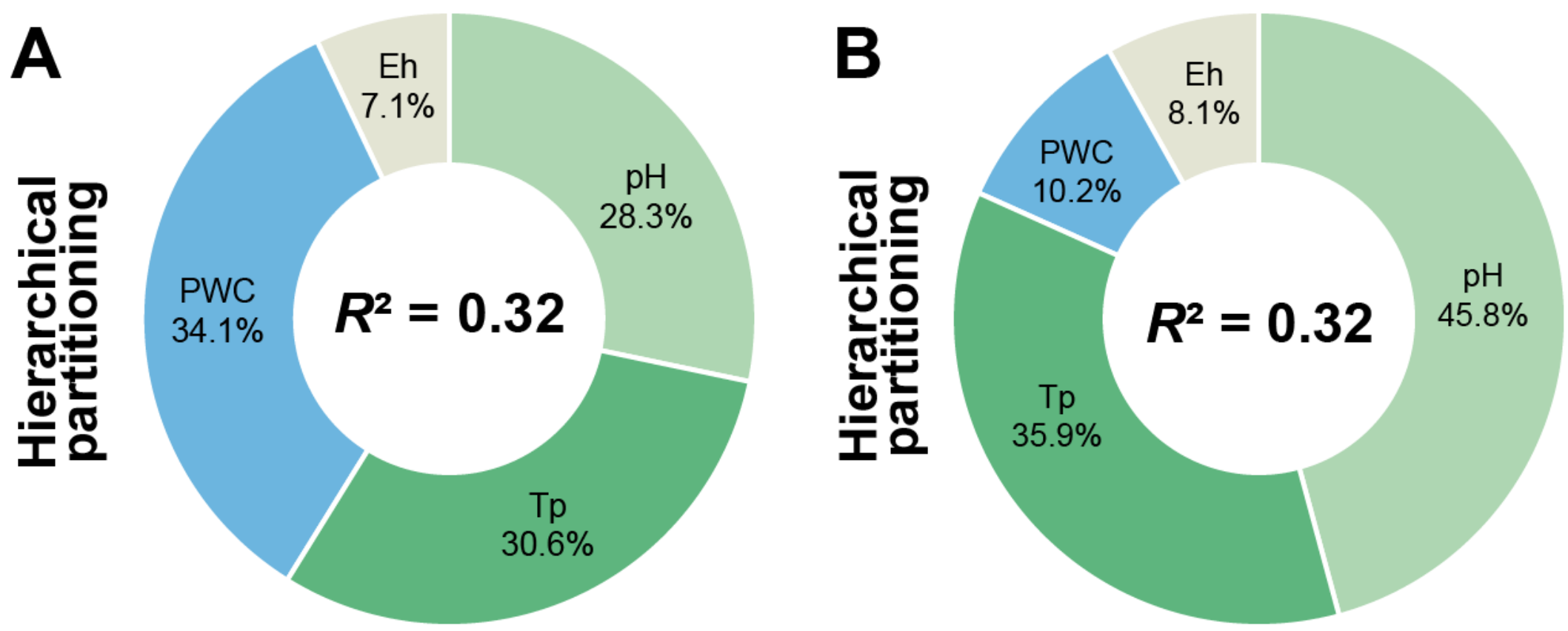


**Supplementary Fig. 4 | Different environmental variables affecting prokaryotic and viral community diversity across vertical profile.** Variance decomposition of environmental variables explaining species-level genome bins (SGBs) Shannon index (**A**) and viral Shannon index (**B**) across vertical profile. Tp, permafrost temperature; PWC, permafrost water content; Eh, redox potential.

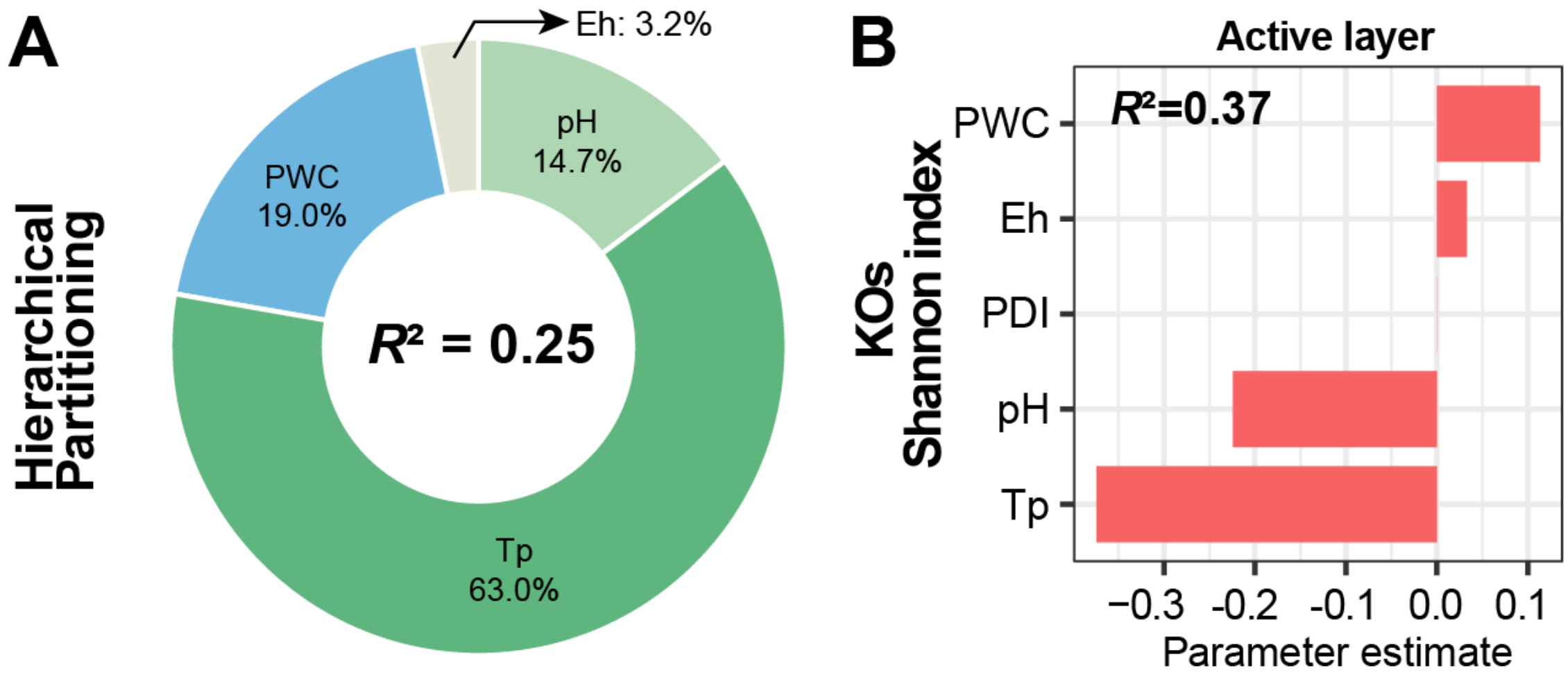


**Supplementary Fig. 5 | Different environmental variables affecting functional gene diversity.** (**A**) Variance decomposition of environmental variables explaining KEGG Orthologs (KOs) Shannon index across vertical profile. (**B**) Relative importance of environmental variables and PDI on KOs Shannon index in the active layer during permafrost degradation. The estimated relative importance value (in %) is standardized regression coefficients in generalized linear model. PDI, permafrost degradation index; Tp, permafrost temperature; PWC, permafrost water content; Eh, redox potential.

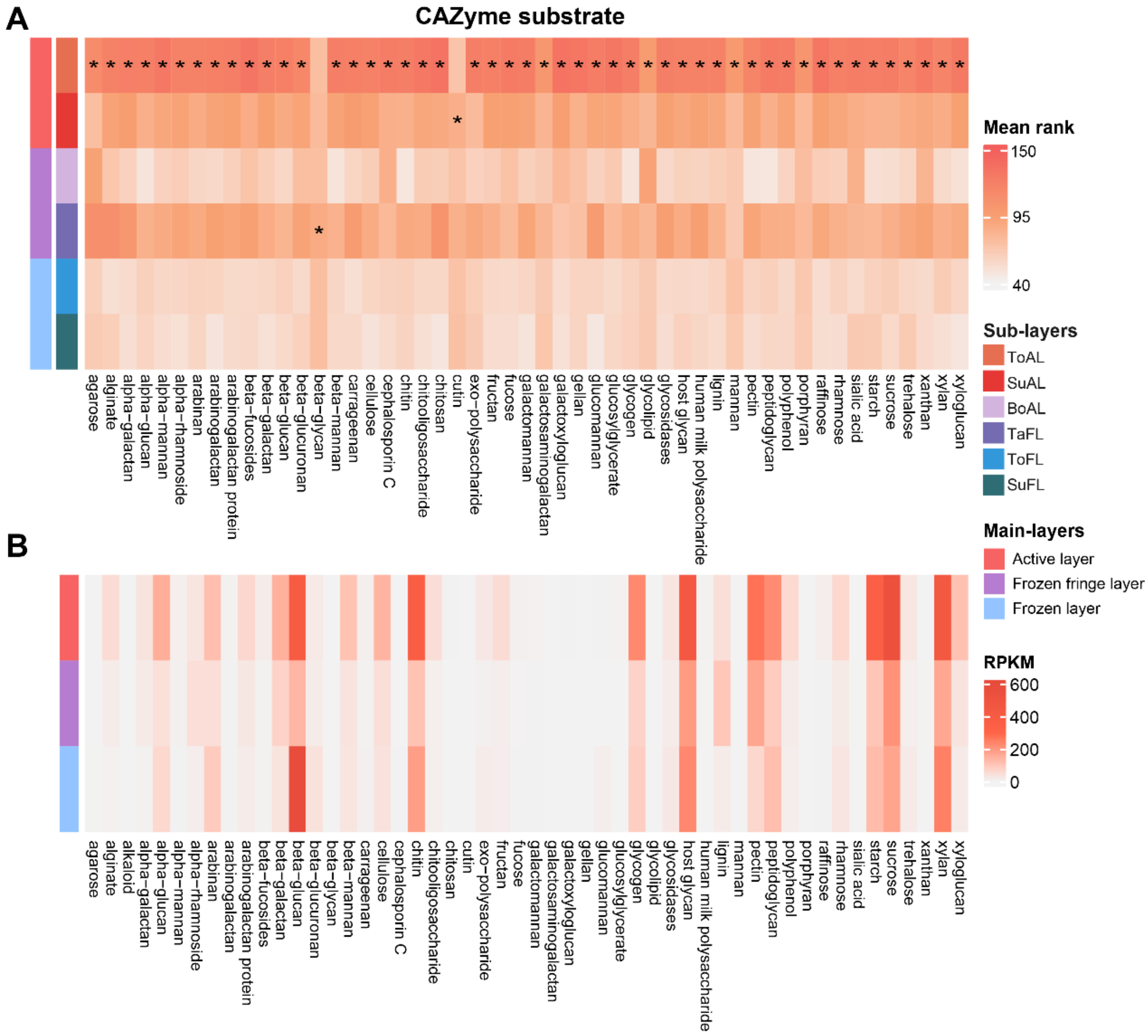


**Supplementary Fig. 6 | Enrichment of carbohydrate-active enzymes (CAZymes) across vertical profile.** (**A**) Substrate-specific enrichment patterns of CAZymes across the main- and sub-layers (Active layer, $n$ = 55, including ToAL, $n$ = 18, and SuAL, $n$ = 37; Frozen fringe layer, $n$ = 12, including BoAL, $n$ = 6, and TaFL, $n$ = 6; Frozen layer, $n$ = 83, including ToFL, $n$ = 59, and SuFL, $n$ = 24). (**B**) RPKM differences in relative abundance of genes capable of metabolizing complex carbohydrate substrates across the main-layers. Statistical significance is based on two-sided Kruskal-Wallis tests. Asterisks indicate statistical significance (*$p < 0.05$). ToAL, top-active layer; SuAL, sub-active layer; BoAL, bottom-active layer; TaFL, table-frozen layer; ToFL, top-frozen layer; SuFL, sub-frozen layer. RPKM, reads per kilobase per million mapped reads.

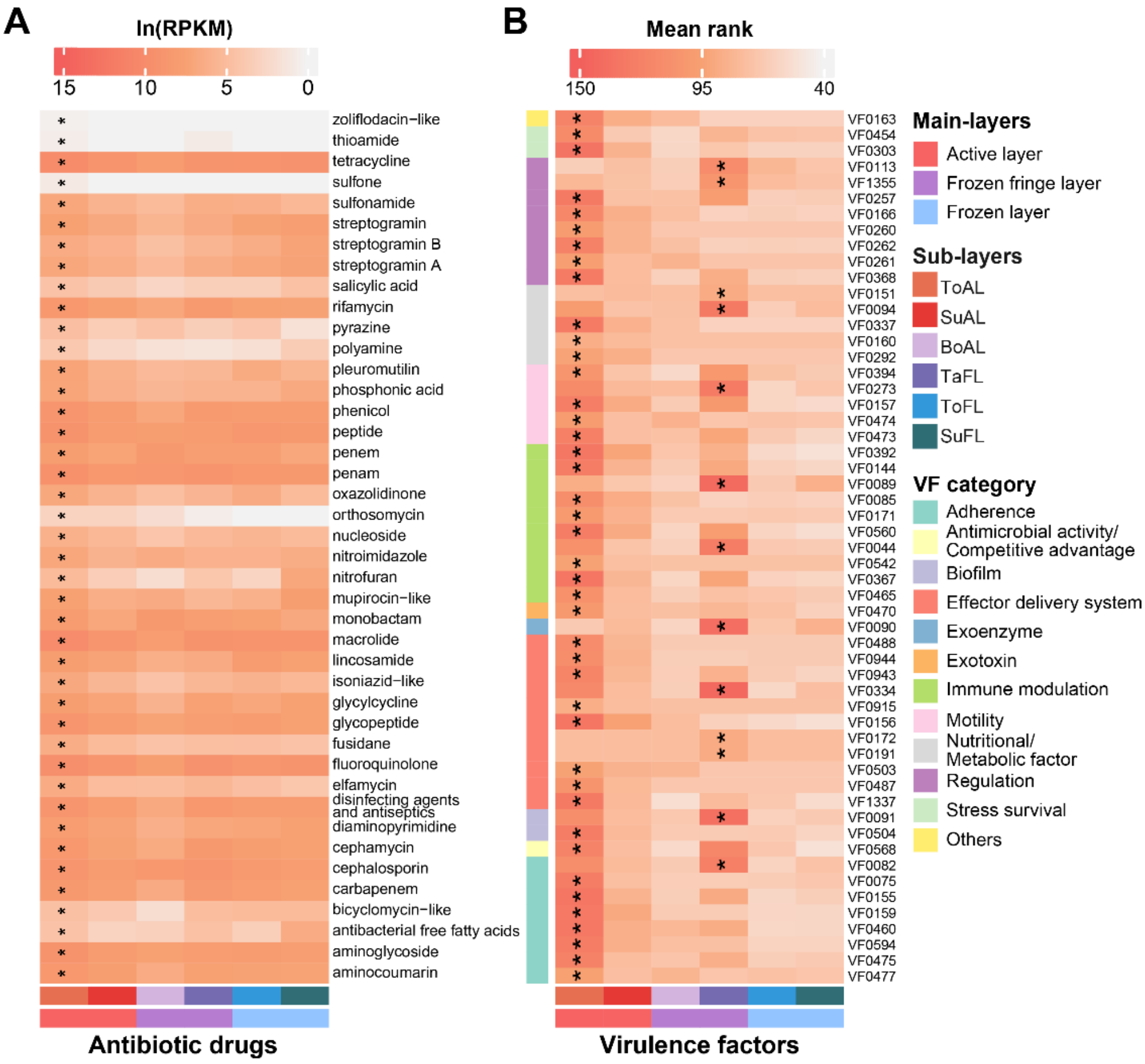


**Supplementary Fig. 7 | Enrichment of antibiotic resistance genes (ARGs) and virulence factors (VFs) across vertical profile.** (**A**) Enrichment of ARGs related to antibiotic drugs across the main- and sub-layers (Active layer, *n* = 55, including ToAL, *n* = 18, and SuAL, *n* = 37; Frozen fringe layer, *n* = 12, including BoAL, *n* = 6, and TaFL, *n* = 6; Frozen layer, *n* = 83, including ToFL, *n* = 59, and SuFL, *n* = 24). (**B**) Enrichment of VFs families across the main- and sub-layers (Active layer, *n* = 55, including ToAL, *n* = 18, and SuAL, *n* = 37; Frozen fringe layer, *n* = 12, including BoAL, *n* = 6, and TaFL, *n* = 6; Frozen layer, *n* = 83, including ToFL, *n* = 59, and SuFL, *n* = 24). Asterisks indicate statistical significance (determined by two-sided Kruskal-Wallis test, *$p < 0.05$). ToAL, top-active layer; SuAL, sub-active layer; BoAL, bottom-active layer; TaFL, table-frozen layer; ToFL, top-frozen layer; SuFL, sub-frozen layer. RPKM, reads per kilobase per million mapped reads.

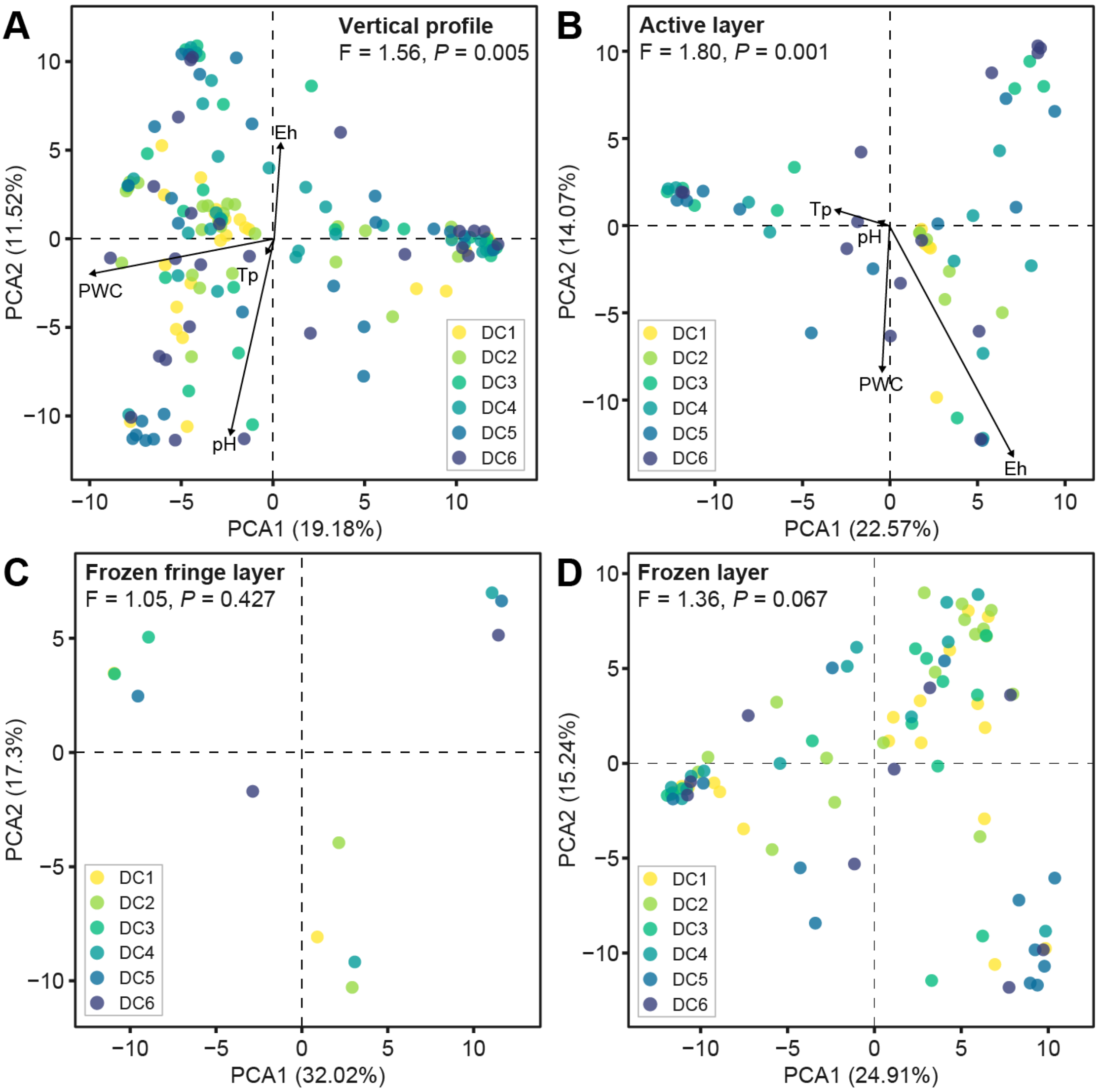


**Supplementary Fig. 8 | Variations and influencing factors of prokaryotic community composition during permafrost degradation.** Principal component analysis (PCA) combined with environmental fitting (envfit) shows the associations of prokaryotic community composition with environmental variables (arrowed lines) across vertical profile (n=150) (**A**) and in the active (DC1: *n* = 5; DC2: *n* = 6; DC3: *n* = 9; DC4: *n* = 10; DC5: *n* = 11; DC6: *n* = 14) (**B**), frozen fringe (*n* = 2) (**C**), and frozen (DC1: *n* = 18; DC2: *n* = 17; DC3: *n* = 14; DC4: *n* = 13; DC5: *n* = 12; DC6: *n* = 9) (**D**) layers during the degradation. Tp, permafrost temperature; PWC, permafrost water content; Eh, redox potential.

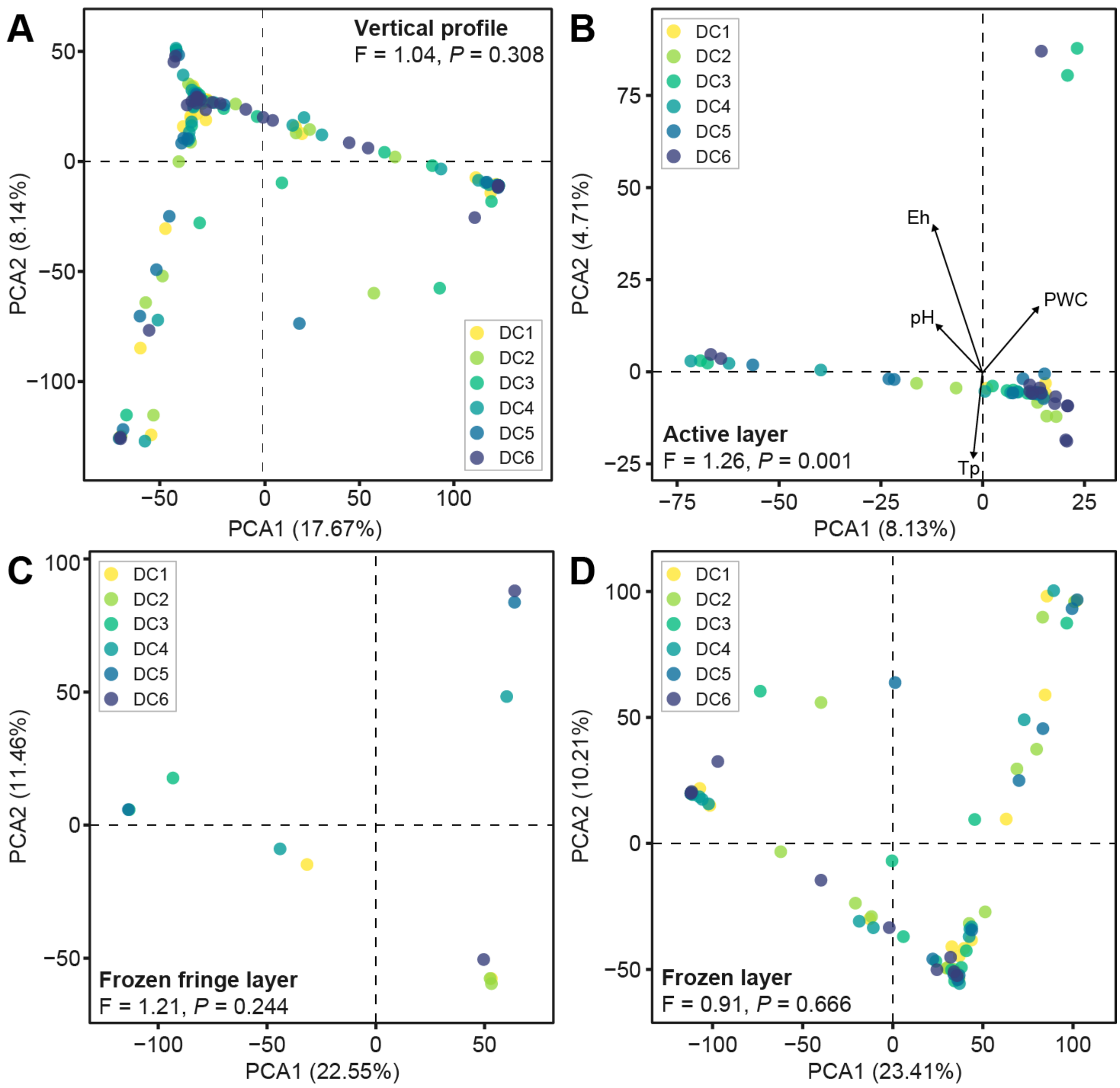


**Supplementary Fig. 9 | Variations and influencing factors of viral community composition during permafrost degradation.** Principal component analysis (PCA) combined with environmental fitting (envfit) shows the associations of viral community composition with environmental variables (arrowed lines) across vertical profile (n=150) (**A**) and in the active (DC1: *n* = 5; DC2: *n* = 6; DC3: *n* = 9; DC4: *n* = 10; DC5: *n* = 11; DC6: *n* = 14) (**B**), frozen fringe (*n* = 2) (**C**), and frozen (DC1: *n* = 18; DC2: *n* = 17; DC3: *n* = 14; DC4: *n* = 13; DC5: *n* = 12; DC6: *n* = 9) (**D**) layers during the degradation. Tp, permafrost temperature; PWC, permafrost water content; Eh, redox potential.

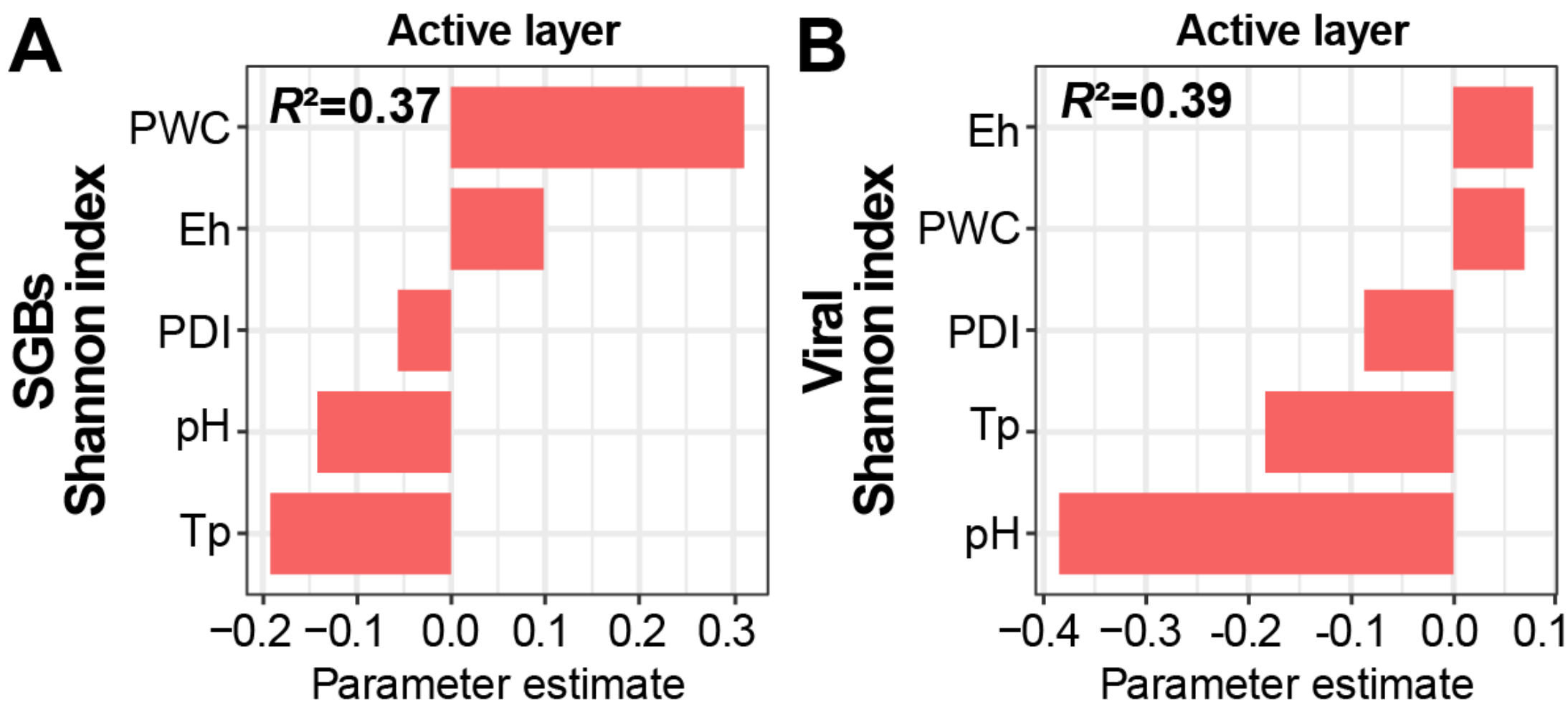


**Supplementary Fig. 10 | Different environmental variables affecting prokaryotic and viral community diversity in the active layer during permafrost degradation.** Relative importance of environmental variables and PDI on species-level genome bins (SGBs) Shannon index (**A**) and viral Shannon index (**B**) in the active layer during the degradation. The estimated relative importance values (in %) are standardized regression coefficients in generalized linear models. PDI, permafrost degradation index; Tp, permafrost temperature; PWC, permafrost water content; Eh, redox potential.

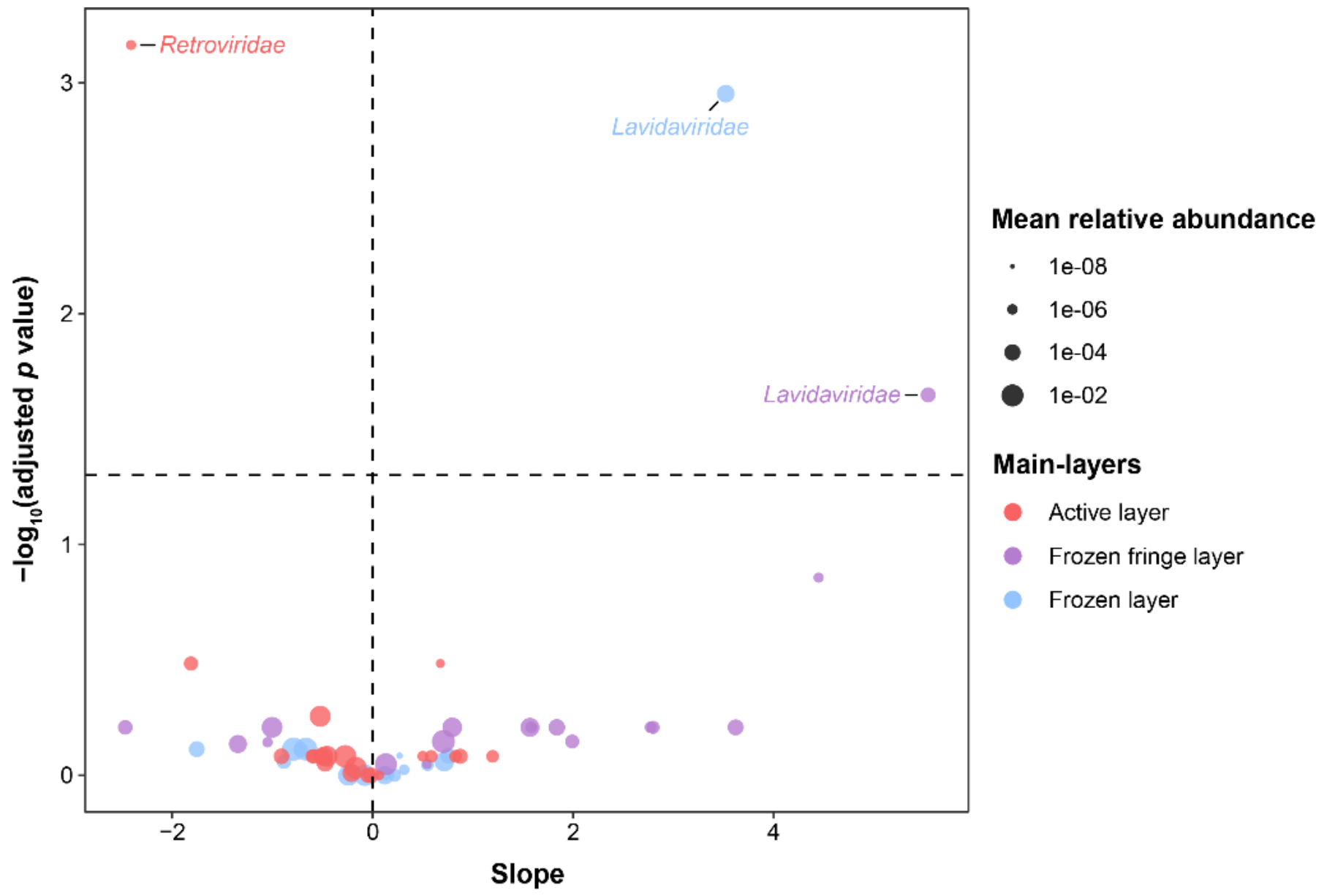


**Supplementary Fig. 11 | Variations in relative abundance of viral families along the degradation gradient.** Correlation between the relative abundance of viral families and PDI across vertical profile. The *p* values between the relative abundance of each viral family and PDI were generated from linear models and adjusted by the Benjamini-Hochberg method. Slopes are the coefficients of linear model. PDI, permafrost degradation index.

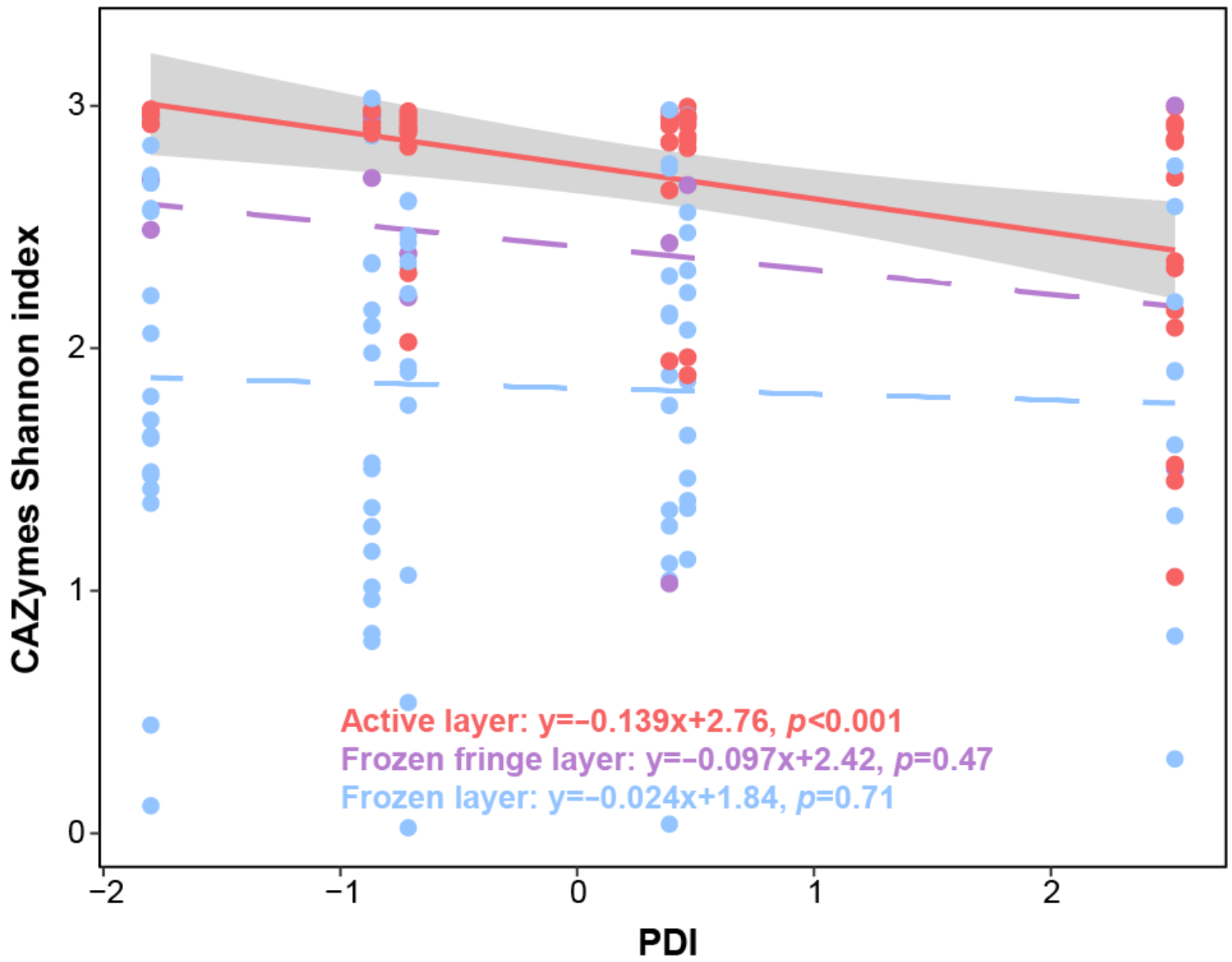


**Supplementary Fig. 12 | Relationships between permafrost degradation index (PDI) and carbohydrate-active enzymes (CAZymes) Shannon index across vertical profile.** Linear relationships between PDI and CAZymes Shannon index across the main-layers. Shaded area represents the 95% confidence interval.

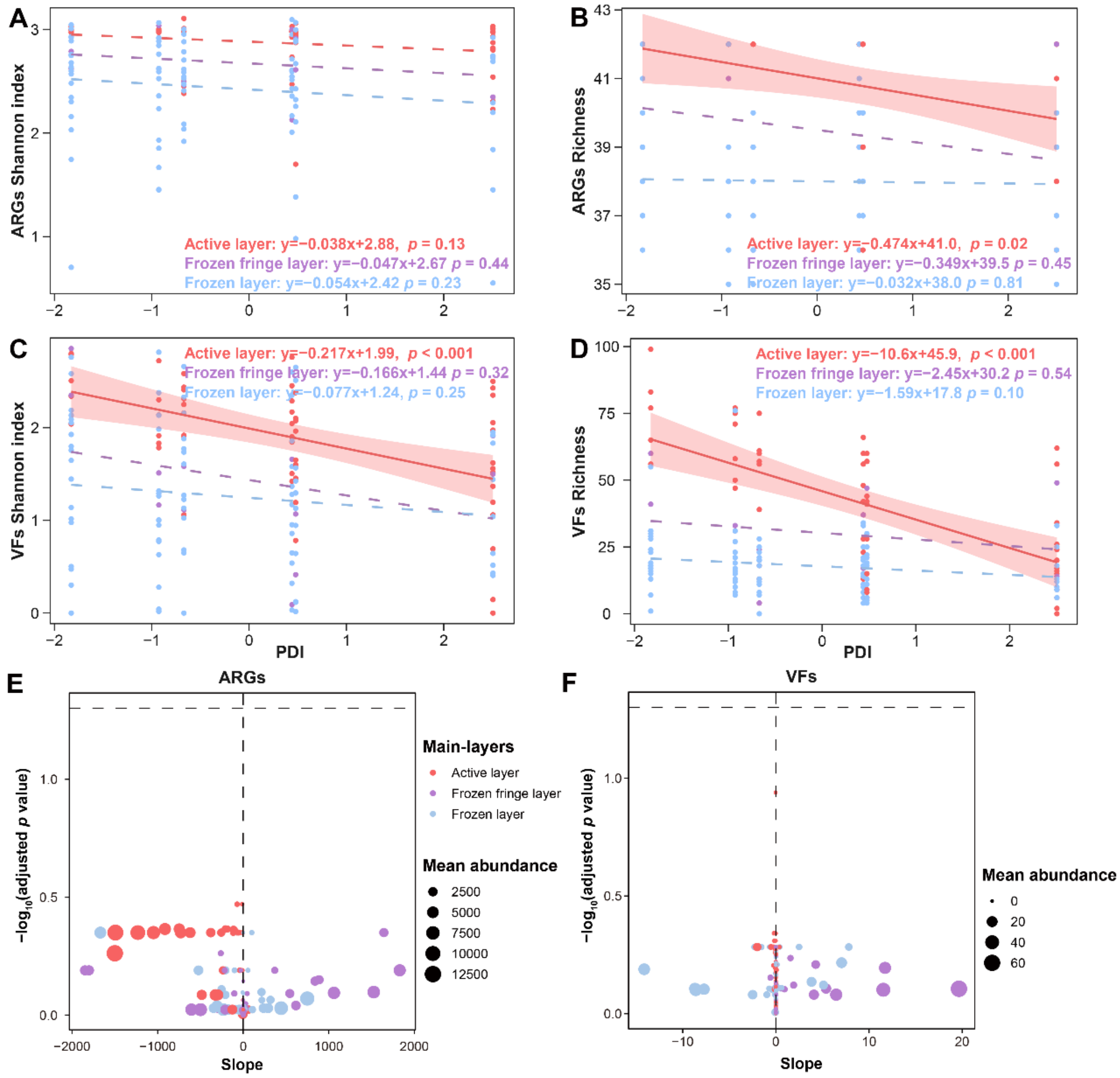


**Supplementary Fig. 13 | Variation of antibiotic resistance genes (ARGs) and virulence factors (VFs) across vertical profile along the degradation gradient.** (**A**) and (**B**) Relationships between PDI and ARGs diversity across vertical profile. (**C**) and (**D**) Relationships between PDI and VFs diversity across vertical profile. (**E**) Correlation between relative abundance of ARGs and PDI across vertical profile. (**F**) Correlation between relative abundance of VFs and PDI across vertical profile. The *p* values between PDI and the relative abundance of each ARG and each VF were generated from linear models and adjusted by the Benjamini-Hochberg method. Shaded area represents the 95% confidence interval. PDI, permafrost degradation index.

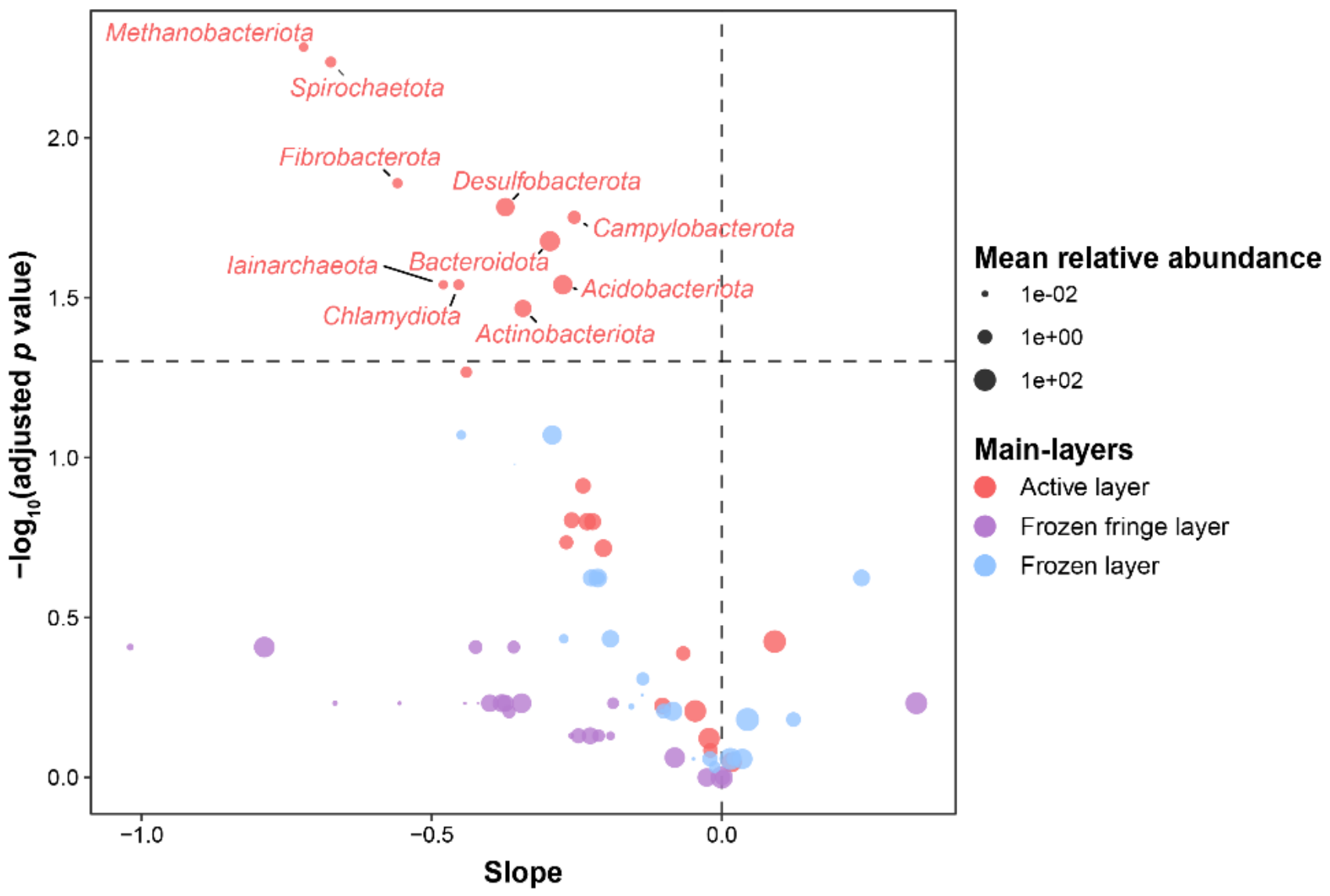


**Supplementary Fig. 14 | Variations in relative abundance of microbial community across vertical profile along the degradation gradient.** Correlation between the relative abundance of microbial phyla and PDI across vertical profile. The *p* value between relative abundance of each phylum and PDI was generated from a linear model and adjusted by the Benjamini-Hochberg method. Slopes are the coefficients of the linear model. PDI, permafrost degradation index.

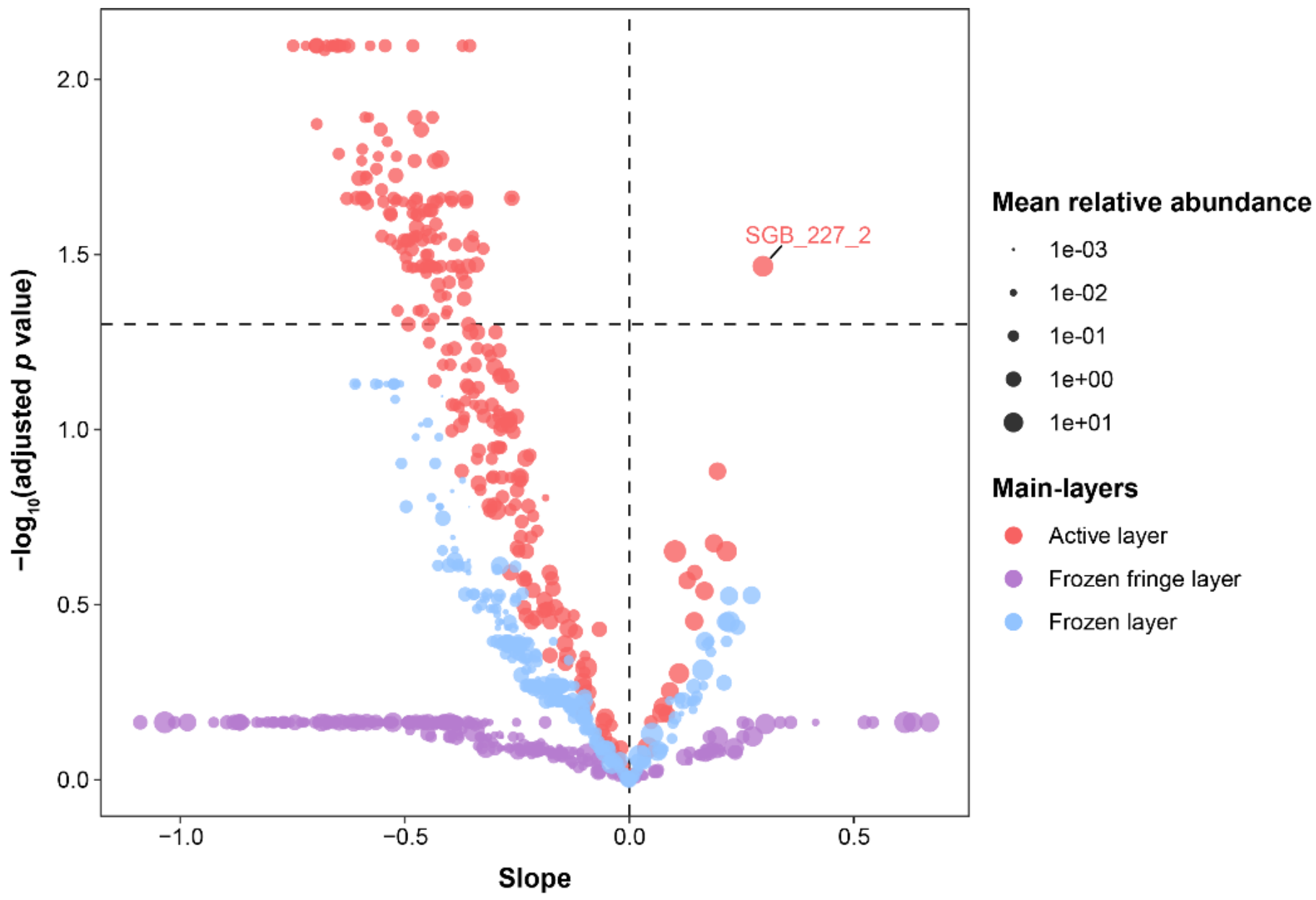


**Supplementary Fig. 15 | Variations in relative abundance of species-level genome bins (SGBs) across vertical profile along the degradation gradient.** Correlation between relative abundance of SGBs and PDI across vertical profile. The *p* value between the relative abundance of each SGB and PDI were generated from linear models and adjusted by the Benjamini-Hochberg method. Slopes are the coefficients of linear model. PDI, permafrost degradation index.

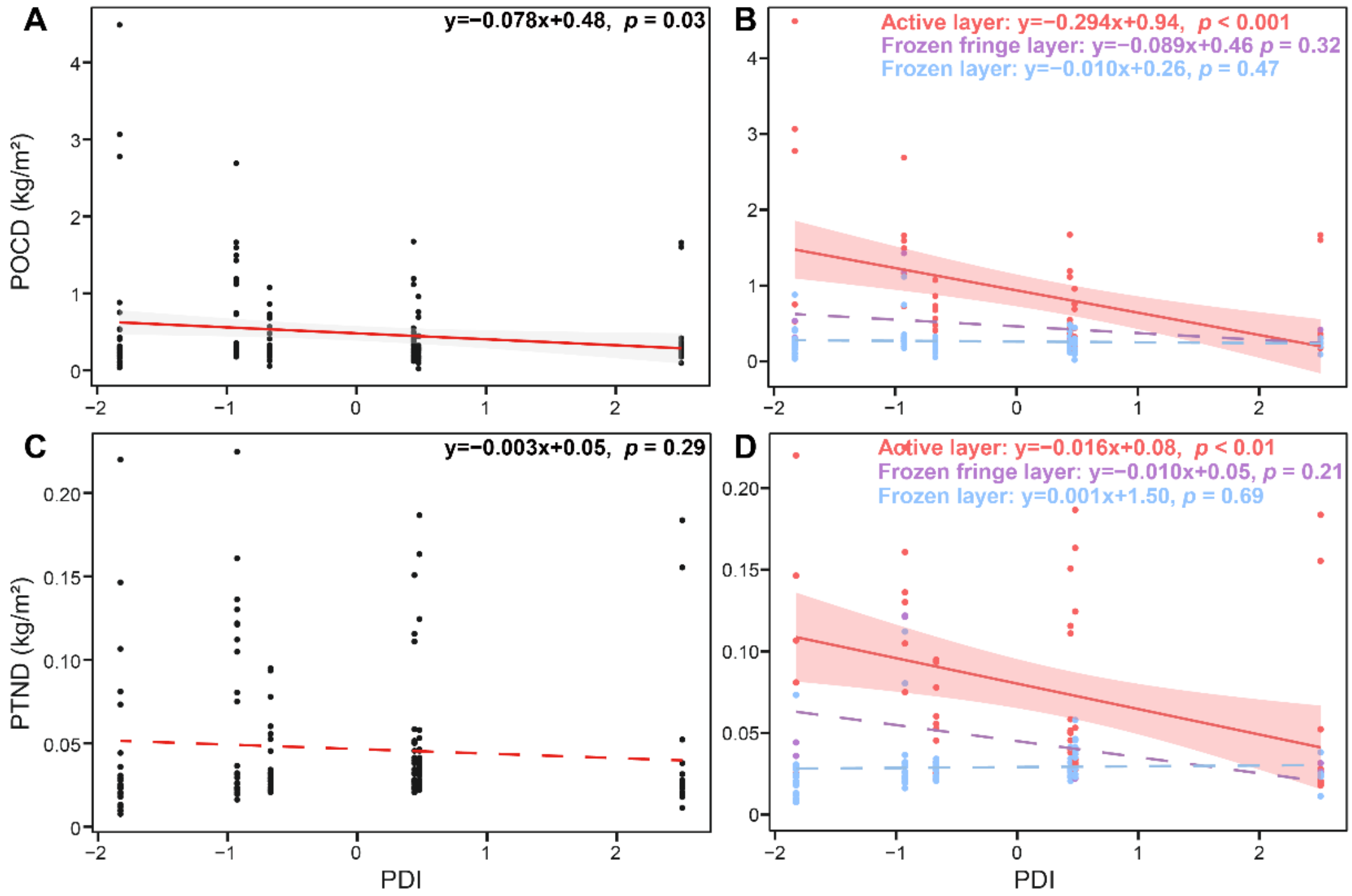


**Supplementary Fig. 16 | Relationships between permafrost organic carbon density (POCD), permafrost total nitrogen density (PTND) and permafrost degradation index (PDI).** (**A**) and (**B**) Relationships between POCD and PDI across vertical profile and at different main-layers, respectively. (**C**) and (**D**) Relationships between PTND and PDI across vertical profile and at different main-layers, respectively. Lines indicate linear fits and shaded area represents the 95% confidence interval.

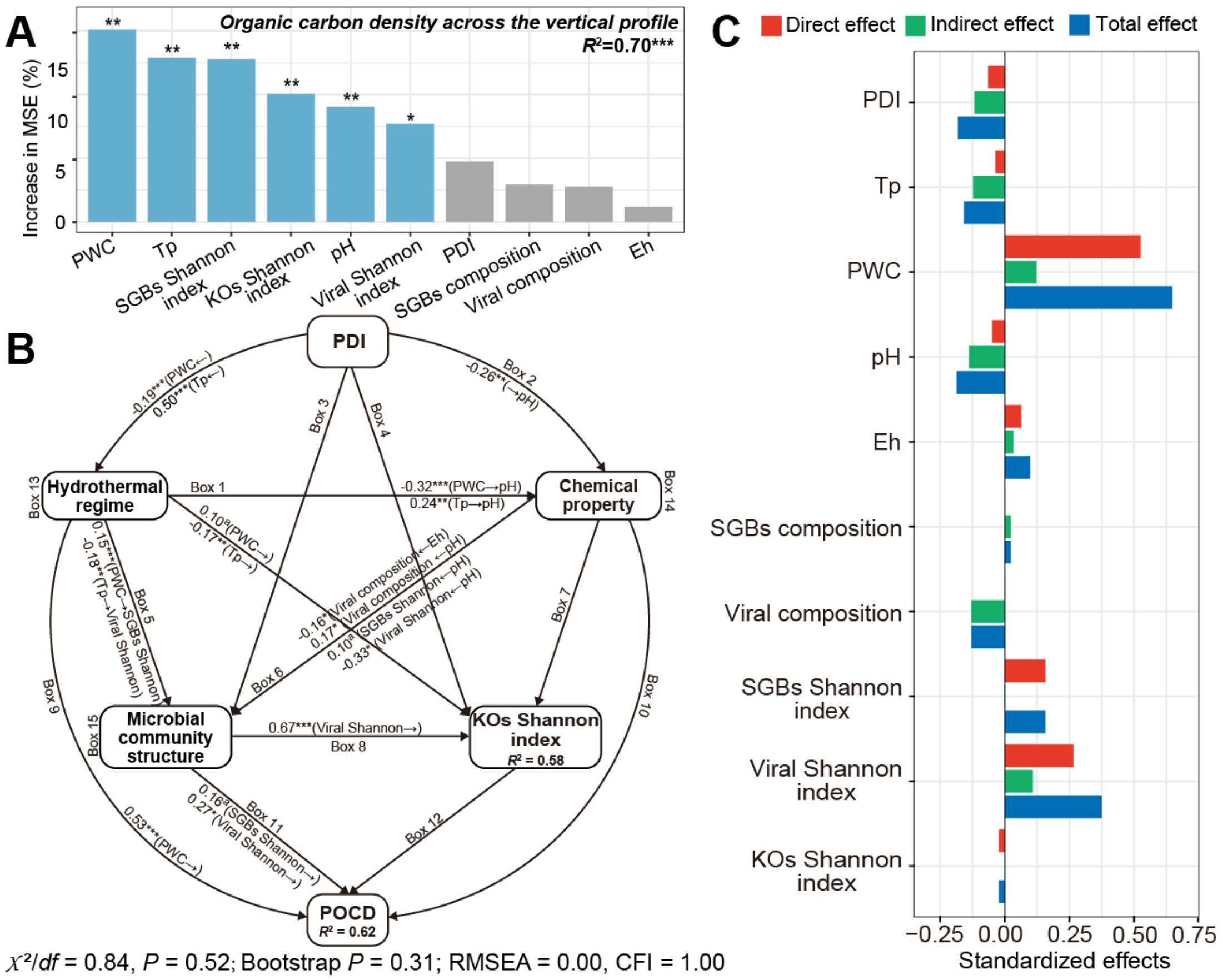


**Supplementary Fig. 17 | Effects of environmental variables and microbiomes on permafrost carbon stock across vertical profile.** (**A**) Importance of PDI, Tp, PWC, pH, Eh, composition and diversity of microbial community, and functional gene diversity on predicting POCD using random forest model across vertical profile. (**B**) Structural equation model showing the effects of PDI, hydrothermal regime, chemical property, microbial community structure, and functional gene diversity on POCD across vertical profile. (**C**) The standardized total, direct, and indirect effects on POCD derived from models, respectively. POCD, permafrost organic carbon density; PDI, permafrost degradation index; Tp, permafrost temperature; PWC, permafrost water content; Eh, redox potential; SGBs, species-level genome bins; KOs, Kyoto Encyclopedia of Genes and Genomes (KEGG) Orthologs; MSE, mean squared error; RMSEA, root mean square error of approximation; CFI, comparative fit index. Asterisks indicate statistical significance (****p* < 0.001, ***p* < 0.01, **p* < 0.05, and [a]*p* < 0.1).

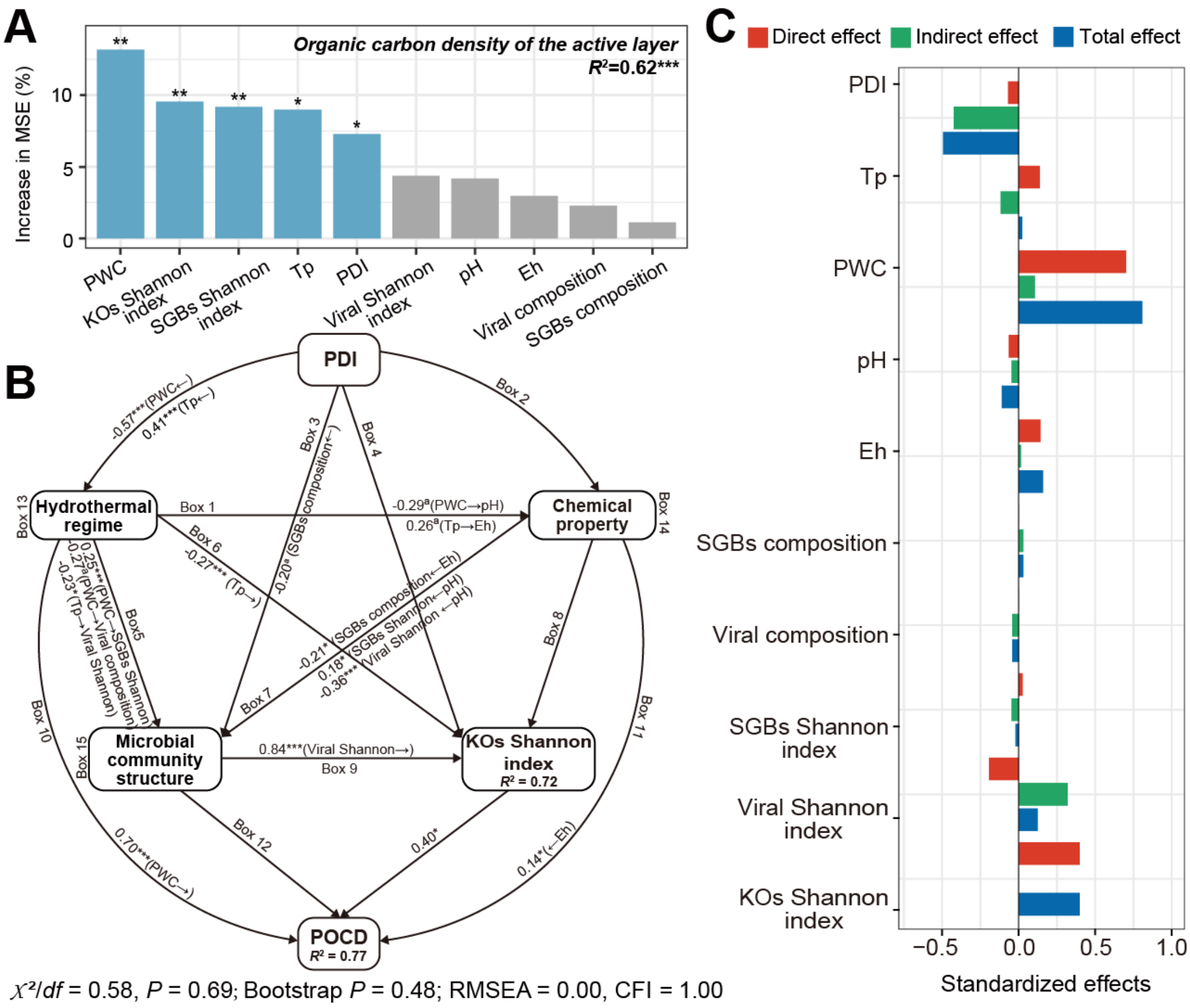


**Supplementary Fig. 18 | Effects of environmental variables and microbiomes on permafrost carbon stock in the active layer.** (**A**) Importance of PDI, Tp, PWC, pH, Eh, composition and diversity of microbial community, and functional gene diversity on predicting POCD using random forest model in the active layer. (**B**) Structural equation model showing the effects of PDI, hydrothermal regime, chemical property, microbial community structure, and functional gene diversity on POCD in the active layer. (**C**) The standardized total, direct, and indirect effects on POCD derived from models, respectively. POCD, permafrost organic carbon density; PDI, permafrost degradation index; Tp, permafrost temperature; PWC, permafrost water content; Eh, redox potential; SGBs, species-level genome bins; KOs, Kyoto Encyclopedia of Genes and Genomes (KEGG) Orthologs; MSE, mean squared error; RMSEA, root mean square error of approximation; CFI, comparative fit index. Asterisks indicate statistical significance (***$p < 0.001$, **$p < 0.01$, *$p < 0.05$, and $^{a}p < 0.1$).

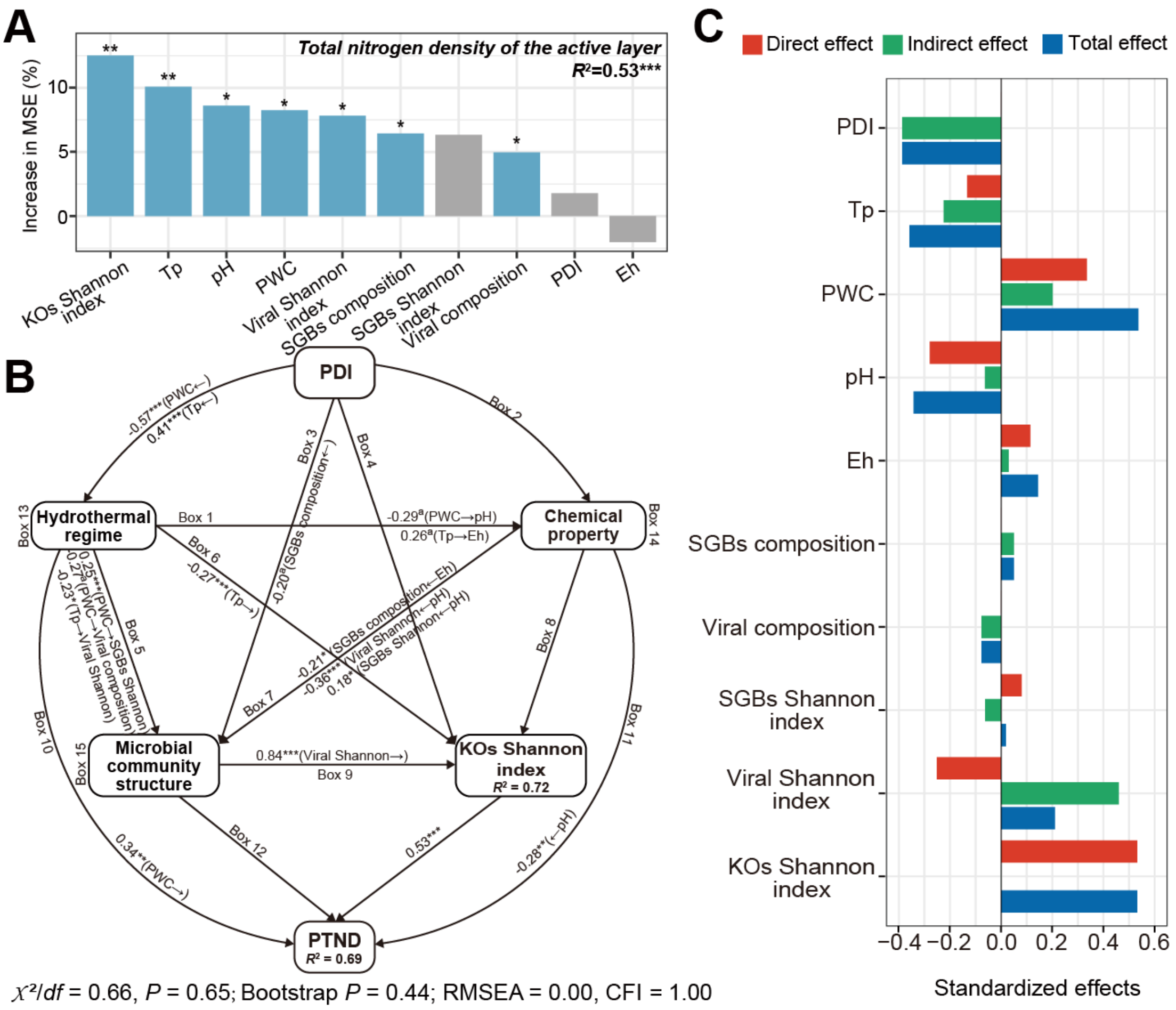


**Supplementary Fig. 19 | Effects of environmental variables and microbiomes on permafrost nitrogen stock in the active layer.** (**A**) Importance of PDI, Tp, PWC, pH, Eh, composition and diversity of microbial community, and functional gene diversity on predicting PTND using random forest model in the active layer. (**B**) Structural equation model showing the effects of PDI, hydrothermal regime, chemical property, microbial community structure, and functional gene diversity on PTND in the active layer. (**C**) The standardized total, direct, and indirect effects on PTND derived from models, respectively. PTND, permafrost total nitrogen density; PDI, permafrost degradation index; Tp, permafrost temperature; PWC, permafrost water content; Eh, redox potential; SGBs, species-level genome bins; KOs, Kyoto Encyclopedia of Genes and Genomes (KEGG) Orthologs; MSE, mean squared error; RMSEA, root mean square error of approximation; CFI, comparative fit index. Asterisks indicate statistical significance (****p* < 0.001, ***p* < 0.01, **p* < 0.05, and $^{a}p$ < 0.1).
