# Supplementary material for: Hydrothermal regimes regulate microbiome patterns across vertical profile and their responses to alpine permafrost degradation
Source: Nat Commun. 2026 Sep 9;17:10636. doi: 10.1038/s41467-026-77448-6 (PMC13650142; doi:10.1038/s41467-026-77448-6)
Supplement: Supplementary file 2 [file 41467_2026_77448_MOESM2_ESM.pdf]

Description of Additional Supplementary Files

File Name: Supplementary Data 1
Description: Statistics of sample sequencing and assembly.

File Name: Supplementary Data 2
Description: Information of permafrost metagenome assembly genomes (MAGs) and species-level genome bins (SGBs) clusters.

File Name: Supplementary Data 3
Description: GTDB taxonomic classification of permafrost SGBs.

File Name: Supplementary Data 4
Description: Wilcoxon test-based enrichment of permafrost SGBs among sub-layers.

File Name: Supplementary Data 5
Description: Variations in microbial dominant phyla/family among different layers.

File Name: Supplementary Data 6
Description: Wilcoxon test-based enrichment of virus families among sub-layers.

File Name: Supplementary Data 7
Description: Wilcoxon test-based enrichment of carbohydrate-active enzymes (CAZymes) genes grouped by CAZyme substrates among sub-layers.

File Name: Supplementary Data 8
Description: Wilcoxon test-based enrichment of antimicrobial resisitant genes (ARGs) grouped by drug classes among sub-layers.

File Name: Supplementary Data 9
Description: Wilcoxon test-based enrichment of virulence factor (VFs) families among sub-layers.

File Name: Supplementary Data 10
Description: Variations in microbial dominant phyla/family along the degradaed gradient.

File Name: Supplementary Data 11
Description: Permafrost properties in the main-layers, sub-layers, and degraded gradient (DC1-DC6).

File Name: Supplementary Data 12
Description: Structure equation modeling boxes showing environmental variables and microbiomes' effects on carbon/nitrogen stock.

File Name: Supplementary Data 13
Description: Sampling depth and grouped information of samples.
