# Supplementary material for: Hydrothermal regimes regulate microbiome patterns across vertical profile and their responses to alpine permafrost degradation
Source: Nat Commun. 2026 Sep 9;17:10636. doi: 10.1038/s41467-026-77448-6 (PMC13650142; doi:10.1038/s41467-026-77448-6)
Supplement: Supplementary file 3 [file 41467_2026_77448_MOESM4_ESM.pdf]

nature portfolio

Corresponding author(s): Shengyun Chen, Yuanqiang Zou, and Mo Han

Last updated by author(s): Jun 30, 2026



# Reporting Summary

Nature Portfolio wishes to improve the reproducibility of the work that we publish. This form provides structure for consistency and transparency in reporting. For further information on Nature Portfolio policies, see our Editorial Policies and the Editorial Policy Checklist.

## Statistics

For all statistical analyses, confirm that the following items are present in the figure legend, table legend, main text, or Methods section.

| n/a | Confirmed | |
|---|---|---|
| ☐ | ☒ | The exact sample size (*n*) for each experimental group/condition, given as a discrete number and unit of measurement |
| ☐ | ☒ | A statement on whether measurements were taken from distinct samples or whether the same sample was measured repeatedly |
| ☐ | ☒ | The statistical test(s) used AND whether they are one- or two-sided<br>*Only common tests should be described solely by name; describe more complex techniques in the Methods section.* |
| ☐ | ☒ | A description of all covariates tested |
| ☐ | ☒ | A description of any assumptions or corrections, such as tests of normality and adjustment for multiple comparisons |
| ☐ | ☒ | A full description of the statistical parameters including central tendency (e.g. means) or other basic estimates (e.g. regression coefficient) AND variation (e.g. standard deviation) or associated estimates of uncertainty (e.g. confidence intervals) |
| ☐ | ☒ | For null hypothesis testing, the test statistic (e.g. *F*, *t*, *r*) with confidence intervals, effect sizes, degrees of freedom and *P* value noted<br>*Give P values as exact values whenever suitable.* |
| ☒ | ☐ | For Bayesian analysis, information on the choice of priors and Markov chain Monte Carlo settings |
| ☐ | ☒ | For hierarchical and complex designs, identification of the appropriate level for tests and full reporting of outcomes |
| ☒ | ☐ | Estimates of effect sizes (e.g. Cohen's *d*, Pearson's *r*), indicating how they were calculated |

*Our web collection on statistics for biologists contains articles on many of the points above.*

## Software and code

Policy information about availability of computer code

| | |
|---|---|
| Data collection | We did not used software and code for data collection. |
| Data analysis | Low-quality reads were filtered using fastp (v0.23.2). Reads that passed the quality control assessment were assembled using a single-sample assembly strategy with MEGAHIT (v1.2.9), retaining contigs exceeding 500 bp in size for subsequent analysis. Metagenomic binning was performed independently for each sample using MetaWRAP (v1.3). The contamination and completeness levels of the MAGs were evaluated using CheckM (v1.2.2) in lineage_wf mode, and only those MAGs meeting the criteria for medium quality as defined by MiMAG47 were retained for further investigation. To enable genome-resolved analyses at SGB level, MAGs were clustered into SGBs using dRep (v3.4.2, parameters: -sa 0.95 -nc 0.30) at a 95% ANI threshold. The representative genome selected from each cluster was defined as an SGB. The SGBs were taxonomically classified using GTDB-Tk (v2.3.2, GTDB release 207). Phylogenetic trees of the SGBs were generated through multiple sequence alignment utilizing the GTDBtk infer and root module. Visualization of the phylogenetic trees was accomplished using the R packages "ggtree" and "ggtreeExtra". To gain insights into the metabolic potential of the permafrost microorganisms, the MAGs were annotated for metabolic functions using METABOLIC (v4.0, default options).<br>The contigs obtained from the metagenomic assembly were utilized as input for gene prediction using Prodigal (v2.6.3). The predicted genes were clustered using the mmseqs2 (v14.7e284) easy-cluster pipeline, employing a threshold of 80% coverage and 90% identity. To functionally annotate the nonredundant genes, eggNOG-mapper (v2.1.11) was employed, utilizing the eggNOG database version 5.0.2. The functional annotations derived from eggNOG-mapper included COG, KEGG, and KO annotations. Furthermore, the genes were searched against the VFDB SetB database using Double Index Alignment of Next-generation sequencing Data (DIAMOND; v2.1.8.162, in 'blastp' mode) with the parameters '-id 90 --query-cover 80 --max-target-seqs 1 --evalue 1e-6'. Additionally, the genes were queried against the Comprehensive Antibiotic Resistance Database (CARD) using RGI (https://github.com/arpcard/rgi, v6.0.2), and the CAZyme annotations were obtained using dbCAN3.<br>To conduct de novo virus prediction from the permafrost metagenome, all assembled contigs were processed using VirSorter2 (v2.2.3), and |

contigs achieving a score greater than 0.7 were retained for further analysis. The potential viral contigs were evaluated subsequently using CheckV (v1.0.1). We retained contigs encoding at least one virus-specific gene and longer than 2 kbp. These contigs were identified as viral genomes. The use of this length threshold can reduce noise from very short and highly fragmented contigs while still capturing a broad spectrum of viral sequences for dereplication and annotation. To facilitate genome dereplication, the viral genomes were clustered into vOTUs using CD-HIT (v4.8.1) with an identity threshold set at > 95%. The identification of prokaryotic hosts for the viral genomes was accomplished through VirHostMatcher-Net, which employs default parameters, and results with prediction scores exceeding 0.8 were considered reliable. Taxonomic assignment of the viral genomes at the family level was conducted using Demovir (https://github.com/feargalr/Demovir).
The clean metagenomic reads were aligned to the SGBs and vOTU using bwa-mem (v0.7.17) with default parameters. Reads that exhibited an alignment identity greater than 95% and an alignment length exceeding 45 nt were retained for subsequent abundance estimation. The relative abundance of the SGBs and vOTUs was calculated on the basis of the alignments obtained using the "rpkm" mode of CoverM (https://github.com/wwood/CoverM, v0.6.1, options: -m rpkm --min-read-aligned-length 45 --min-read-percent-identity 95 in genome mode). Furthermore, translation mapping to the gene catalog was performed using DIAMOND (v2.1.8.162) in "blastx" mode with the options '-id 90 --evalue 1e-6 -k 1 --max-hsps 1'.
All the statistical analyses were performed in R (v4.4.1), and the "ggplot2" package was used to visualize the data. The environmental variables considered in this study including hydrothermal regimes (Tp and PWC) and chemical properties (pH and Eh). We performed the Kruskal-Wallis test to evaluate differences in permafrost environmental variables and microbiomes. The prokaryotic and viral community compositions were represented by the first axis of the PCA, and envfit analysis in the "vegan" package was used to analyze the relationship between microbial community composition and environmental variables. The "rdacca.hp" and "glmm.hp" packages were used to construct linear mixed-effects models to quantify the impact of environmental variables on SGBs and viral Shannon index. Generalized linear models were used to assess the relative influence of environmental variables and PDI on diversity with the "h2o" package. The relative importance of PDI, environmental variables, and microbiomes in explaining carbon/nitrogen stocks was assessed by random forest models using "rfPermute" and "randomForest" packages. Considering that samples originated from multiple profile layers within cores from six drilling sites, sample nesting was subsistent in the construction of the SEM. To evaluate the nesting effect, we first calculated intraclass correlation coefficients (ICC) using SPSS 27.0. The nesting effect (also known as the design effect, Deff) was calculated using the formula [$Deff = 1 + (m - 1) \times ICC$], where m represents the average cluster size. For the sample data used in SEMs, all Deff values < 2 that indicate the ignorable nesting effect. Each SEM was constructed by AMOS 24.0 after a priori hypothesis regarding the relationships among permafrost degradation parameter, environmental variables, microbiomes, and carbon/nitrogen stock. Finally, the model fit was evaluated using multiple indices, including the ratio of chi-square to degrees of freedom ($\chi^2$/df), the root means square error of approximation (RMSEA), the comparative fit index (CFI), and the p-value of the Bollen-Stine bootstrap test. The model was considered to be acceptable with $0 \le \chi^2/df \le 2$, RMSEA < 0.05, CFI > 0.9, and p > 0.1.

For manuscripts utilizing custom algorithms or software that are central to the research but not yet described in published literature, software must be made available to editors and reviewers. We strongly encourage code deposition in a community repository (e.g. GitHub). See the Nature Portfolio guidelines for submitting code & software for further information.

## Data

Policy information about availability of data

All manuscripts must include a data availability statement. This statement should provide the following information, where applicable:

- Accession codes, unique identifiers, or web links for publicly available datasets
- A description of any restrictions on data availability
- For clinical datasets or third party data, please ensure that the statement adheres to our policy

The sequencing data generated in this study have been deposited in the Genome Sequence Archive in National Genomics Data Center, China National Center for Bioinformation/Beijing Institute of Genomics, Chinese Academy of Sciences (GSA: CRA045482) that are publicly accessible at https://ngdc.cncb.ac.cn/gsa. Source data are provided with this paper.

## Research involving human participants, their data, or biological material

Policy information about studies with human participants or human data. See also policy information about sex, gender (identity/presentation), and sexual orientation and race, ethnicity and racism.

| | |
|---|---|
| Reporting on sex and gender | Our study does not involve information on sex and gender. |
| Reporting on race, ethnicity, or other socially relevant groupings | Our study does not involve information on ethnicity, or other socially relevant groupings. |
| Population characteristics | Our study does not involve information on population characteristics. |
| Recruitment | Our study does not involve information on recruiting volunteers. |
| Ethics oversight | Not applicable. This study does not involve human participants, their data, or biological material. |

Note that full information on the approval of the study protocol must also be provided in the manuscript.

# Field-specific reporting

Please select the one below that is the best fit for your research. If you are not sure, read the appropriate sections before making your selection.

☐ Life sciences ☐ Behavioural & social sciences ☒ Ecological, evolutionary & environmental sciences

For a reference copy of the document with all sections, see [nature.com/documents/nr-reporting-summary-flat.pdf](nature.com/documents/nr-reporting-summary-flat.pdf)

# Ecological, evolutionary & environmental sciences study design

All studies must disclose on these points even when the disclosure is negative.

| | |
|---|---|
| Study description | This is an observational field study of alpine permafrost in the Shule River Headwaters on the western Qilian Mountains, northeastern margin of the Qinghai-Tibet Plateau, China. The study employs a stratified hierarchical design structured around two primary natural environmental factors: a horizontal permafrost degradation gradient and vertical depth profiles (spanning from the active layer to the frozen layer). The primary experimental samples were selected from six 15-meter drilling cores across six distinct sites. Different degradation levels and stratigraphic layers serve as the treatments. Robust replication is achieved spatially across the six sites and vertically across multiple key layers within each core. |
| Research sample | The research sample comprises 150 permafrost soil samples collected from six 15-m deep drilling cores (sampled at 10 cm intervals) in the Qinghai-Tibet Plateau. Cores were extracted aseptically without drilling fluids, and the outer 1 cm was removed to prevent contamination. |
| Sampling strategy | No statistical methods were used to predetermine sample size. From the total 900 field samples collected continuously along the six 15-meter cores, a representative subset of 150 samples was purposively selected for downstream metagenomic sequencing and comprehensive physicochemical analyses. This sampling strategy was specifically designed to systematically cover the distinct permafrost degradation gradients and to capture the key stratigraphic layers across the vertical profiles. This structured sample size ensures high spatial and vertical resolution, providing sufficient replication within each degradation stage and stratigraphic layer. It yields a highly robust dataset that statistically empowers multi-factor ecological modeling and non-parametric multivariate analyses to resolve vertical microbiome distribution without limiting statistical power. |
| Data collection | The Hydra Probe II soil sensors (Stevens) and the temperature chains equipped with thermistors developed by the State Key Laboratory of Cryospheric Science and Frozen Soil Engineering were connected to the CR1000X datalogger (Campbell Scientific), automatically monitoring Tp at the corresponding drilling site from 0.1 to 15 m depths in each borehole and recording data at 10 minutes intervals. The S.C., E.L. and P.W. periodically retrieved and downloaded the data using a laptop. |
| Timing and spatial scale | All field samples were collected in October 2020. Spatially, the study area is located in the Shule River Headwaters (38.0–39.0° N, 97.3–99.0° E, 3,402–5,814 m a.s.l.) |
| Data exclusions | No permafrost samples or laboratory measurements were excluded from the analyses. |
| Reproducibility | All field cores were collected using the same drilling equipment and procedures, and all physiochemical measurements followed standard, widely used analytical protocols. The study design includes multiple sites and multiple depth intervals, providing spatial and vertical replication of observed patterns. Data processing and statistical analyses were implemented using scripted workflows that can be rerun to reproduce the results. All attempts to repeat the experiments, including technical replications of DNA extraction and iterative reruns of the computational pipelines, were successful. |
| Randomization | This is an observational field study of permafrost, and sites were not randomly assigned to treatments. We purposively selected six sites along a permafrost degraded gradient. |
| Blinding | Blinding was not applicable during field sampling, as site locations and vertical depth profiles were predetermined based on geographical coordinates and drilling depths. Group allocation along the degradation gradient was defined using objective physical measurements rather than subjective assessment. For downstream laboratory processes, including microbial DNA extraction and sequencing, blinding was not performed. All subsequent data processing, metagenomic profiling, and statistical modeling were executed using standardized computational pipelines. |

Did the study involve field work? ☒ Yes ☐ No

## Field work, collection and transport

| | |
|---|---|
| Field conditions | Study area belongs to the continental arid desert climate, with mean annual air temperature of -0.96 to -5.62°C and precipitation of 201 to 532 mm based on the monitoring meteorological data. The dominant vegetation type is alpine grassland including alpine marsh meadow, alpine meadow, and alpine steppe. Alpine permafrost is distributed extensively and occupies about 97.99% of the area, with the active layer thickness ranging from 1.2 to 6.0 m and ground temperature at a depth of 15 m ranging from -0.05 to -0.75°C on the basis of the monitoring data. |
| Location | The study area is located in the Shule River Headwaters (38.0 to 39.0° N, 97.3 to 99.0° E, and 3,402 to 5,814 m a.s.l.) on the western part of the Qilian Mountains, northeast margin of the QTP, China. |

| | |
|---|---|
| Access & import/export | Field work and sampling were organized by the State Key Laboratory of Cryospheric Science and Frozen Soil Engineering and conducted in accordance with relevant local and national regulations for permafrost regions in China. Access to the field sites and the collection of permafrost cores were fully permitted and supported by the Northwest Institute of Eco-Environment and Resources, Chinese Academy of Sciences. No international import or export of whole-soil samples was involved; samples were transported in liquid nitrogen or on ice directly to the laboratory in China for further processing and analysis. |
| Disturbance | Disturbance to the ecosystem was minimized. At each site, only a single, narrow-diameter 15-m core was drilled using a vibratory coring machine with stainless-steel tubes, without the use of drilling fluids or water. The outer 1 cm of each core was removed in the laboratory for contamination control rather than additional field disturbance. Vegetation removal around the boreholes was kept to the minimum required for safe drilling, and boreholes were backfilled after coring. No long-term infrastructure or repeated mechanical disturbance was introduced at the sites. |

# Reporting for specific materials, systems and methods

We require information from authors about some types of materials, experimental systems and methods used in many studies. Here, indicate whether each material, system or method listed is relevant to your study. If you are not sure if a list item applies to your research, read the appropriate section before selecting a response.

## Materials & experimental systems

| n/a | Involved in the study |
|---|---|
| ☒ | ☐ Antibodies |
| ☒ | ☐ Eukaryotic cell lines |
| ☒ | ☐ Palaeontology and archaeology |
| ☒ | ☐ Animals and other organisms |
| ☒ | ☐ Clinical data |
| ☒ | ☐ Dual use research of concern |
| ☒ | ☐ Plants |

## Methods

| n/a | Involved in the study |
|---|---|
| ☒ | ☐ ChIP-seq |
| ☒ | ☐ Flow cytometry |
| ☒ | ☐ MRI-based neuroimaging |

## Plants

| | |
|---|---|
| Seed stocks | Not applicable. This study did not use any plant or seed material. |
| Novel plant genotypes | Not applicable. This study did not generate or use any novel plant genotypes. |
| Authentication | Not applicable. No plant seed stocks or novel plant genotypes requiring authentication were used in this study. |
