# Supplementary material for: Hydrothermal regimes regulate microbiome patterns across vertical profile and their responses to alpine permafrost degradation
Source: Nat Commun. 2026 Sep 9;17:10636. doi: 10.1038/s41467-026-77448-6 (PMC13650142; doi:10.1038/s41467-026-77448-6)
Supplement: Supplementary file 4 [file 41467_2026_77448_MOESM5_ESM.pdf]

nature portfolio

Peer Review File

Hydrothermal regimes regulate microbiome patterns across vertical profile and their responses to alpine permafrost degradation

Corresponding Author: Professor Shengyun Chen

This file contains all reviewer reports in order by version, followed by all author rebuttals in order by version.

Version 0:

Reviewer comments:

Reviewer #1

(Remarks to the Author)
This manuscript presents a large and carefully generated metagenomic dataset from alpine permafrost on the Qinghai-Tibet Plateau (QTP), including 501 MAGs, 28,008 viral genomes, and 43 million non-redundant genes derived from 150 samples collected from six 15-m-deep permafrost cores along a degradation gradient. Through systematic multi-layer sampling and explicit division of the active, frozen fringe, and frozen layers, this study substantially extends the vertical scope of permafrost microbiomes. The integrated analysis of microbiomes and environmental variables allows the authors to characterize depth- and degradation-related variations in the microbiome, and to link hydrothermal regimes, microbiome patterns, and carbon/nitrogen loss under permafrost degradation. Collectively, the work provides a valuable genomic resource and offers novel insights into the roles of biotic and abiotic factors in affecting biogeochemical processes and potential biosafety implications in degrading permafrost of QTP. However, I have some suggestions and comments about the current version of the manuscript, which I outline below.

My major concerns:
1. There is a strong bias in MAG recovery toward the active layer, with substantially fewer genomes reconstructed from deeper layers (frozen fringe and frozen layers). It should be explicitly discussed regarding its potential impact on conclusions about deeper layers. For example, some of the apparent contrasts between the active and frozen fringe/frozen layers may partly reflect differential assembly and binning efficiency rather than true biological absence or rarity. Clarifying this limitation will help readers interpret the depth-related patterns more cautiously.
2. The high proportion of unclassified viral genomes (74%) is striking and merits explicit commentary. It would be helpful to discuss whether this primarily reflects true viral novelty in alpine permafrost or current limitations of reference databases. A brief comparison with Arctic permafrost viromes would provide valuable context.
3. Several central claims (e.g., “hydrothermal regimes as primary drivers”, “microbiomes exert the largest total effect on POCD”) currently rest on correlative modeling and should be presented with appropriate caution. Besides, while the SEMs propose plausible pathways, the language should be tempered to reflect their correlative nature, favoring terms like "associations" over "regulatory pathways" unless causal mechanisms are experimentally validated.
4. The manuscript refers to “carbon/nitrogen loss”, “exacerbated carbon release”, and “increased N_2O emissions”, while the data are based on spatial comparisons among sites with different PDI values, measuring POCD/PTND and functional gene abundances/ratios ((nirK+nirS)/nosZ), without temporal data or direct flux measurements. Additionally, the increased (nirK+nirS)/nosZ ratio is presented as an indicator of N_2O emission potential. It is important to clarify in the text that this reflects a genetic capacity, not confirmed in situ activity, which would require complementary transcriptomic or isotopic data.
5. The Discussion would benefit from an expanded exploration of potential drivers unique to alpine permafrost, such as substrate quality, microbial thermal adaptation, or redox history. In particular, the reported decrease in CAZyme diversity in the active layer along the degradation gradient, opposite to some previous findings, is a very interesting result. You attribute this pattern to shifts in substrate availability and quality toward more labile carbon. While this mechanism is plausible, no direct measurements of substrate quality are presented. I suggest explicitly framing this explanation as a hypothesis generated from gene profiles rather than a fully demonstrated mechanism, and using cautious wording in the Discussion.
6. The enrichment of viruses and virulence factors in the frozen fringe is intriguing. However, regarding biosafety (L348-353), the discussion remains speculative. I suggest moderating the language unless direct links to known pathogens or evidence of mobility for the detected ARGs/VFs are established, to maintain a precise and evidence-based tone. In addition, there is

no direct evidence of viability or activity of these viruses or hosts, nor genomic context analyses showing ARGs/VFs on plasmids, phages, or integrative elements, nor comparisons with contemporary environmental communities.
7. The presentation of methods and supplementary materials should be improved to enhance reproducibility and transparency. The DNA extraction method cites a patent; a brief summary of key modifications would aid reproducibility. In addition, serval supplementary tables are currently unreadable due to formatting errors. These must be corrected and verified upon revision.
8. Language and data availability: The overall structure of the manuscript is clear and figures are well integrated, but the text contains numerous grammatical and stylistic issues (articles, prepositions, plural forms, awkward collocations, very long noun phrases). A thorough language edit by a fluent English speaker or professional service is recommended, especially for the complex sentences. Additionally, the manuscript currently lacks a dedicated Data Availability statement. Based on open data science policies and fair principles, the sequencing data should be deposited in public repositories, and accession numbers and links should be provided to ensure transparency and reusability.

Other minor issues:
1. L29: "in the boundary" should be "at the boundary".
2. L67: replace “Naturally” with “As expected”.
3. L73-74: "the diminished" should be "the diminishing", "the potential increases" should be "the potential increase".
4. L133 and L393: phrases like “It was worth noting that…” or “It was worth stressing that…” could be streamlined to “Notably, …” or “Importantly, …” for more natural English.
5. L175: "Candidates" is ambiguous in this context. "Detected" or "identified" is more precise for metagenomic data.
6. L200-201: "exhibited"? Should carefully check the tense throughout the manuscript.
7. L205: avoid using subjective words such as 'obviously'.
8. L266: "across the vertical profile"?
9. L340: "upon the degradation"?
10. L394 and L416-417: The manuscript states that 150 samples were collected from six 15 m cores, yet the Methods also mention “one sample was collected every 10 cm depth… yielding a total of 150 samples” and later “25 samples… were selected from 150 samples of each drilling core”. The latter phrasing appears inconsistent with the earlier total of 150 samples and creates confusion about how many samples per core and per layer were actually sequenced.
11. L413: “Furtherly” is not common use in standard academic English.
12. L491: “to test for differences”?
13. Please standardize terminology such as “main (-) layers” vs “main layers”, and consider using “degradation gradient” rather than “degraded gradient”.
14. Fig1: What is the sample size for each layer? For example, plots showing Shannon index comparisons and regressions, sample sizes should be provided.
15. The number of supplementary figures appear excessive; I suggest moving some of them to the extended data figure.
16. A few references show minor formatting inconsistencies (journal name abbreviation, hyphenation, etc.). Please harmonize them according to the journal’s reference style.

Overall, this study fully advances the field and will be of interest to researchers in microbial ecology, cryospheric science, and environmental genomics. The English is generally clear but contains numerous grammatical and stylistic issues, and some causal and risk-related statements appear overstated. Substantial revisions are still needed, particularly regarding data interpretation, statistical nuance, and the accessibility of the supplementary files. I recommend acceptance after major revisions, on the condition that the authors adequately address the points above.

Reviewer #2

(Remarks to the Author)
This manuscript demonstrated a deep metagenomic analysis of 150 samples across six 15-m permafrost cores in Qinghai-Tibet Plateau area, and found that degradation specifically reduces microbial diversity in the active layer while enriching viruses and virulence factors in the frozen fringe. This kind of change was primarily driven by hydrothermal regimes. Notably, these hydrothermal and microbiome attributes jointly regulate carbon/nitrogen loss in response to future warming permafrost systems. The manuscript was well-written and the results could well-support their findings, I only have several concerns for the clarification of key concepts and data interpretations.

Major comments:
1. The authors used active thickness and ground temperature to calculate a degradation index to classify the gradient of permafrost soils of six drilling cores. However, shallow active layer thickness does not necessarily indicate severe permafrost degradation. Because permafrost degradation is fundamentally defined by a persistent thickening trend of the active layer and rising ground temperatures, rather than absolute depth. Additional evidence to show the degradation tendency of these six drilling cores may strengthen the findings, such as monitory data for active layer thickness changes, ground plant community changes or ground temperature changes in continuous years.
2. The sequencing depth for each sample was relatively huge as 50 Gb of sequencing data could result in solid results. But according to the assembly results, the assembly quality and efficiency might be poor due to the highly fragmented reads of pair-end 100 bps sequencing technology. A basic assessment of sequencing depth for these samples is needed to evaluate the diversity profillings. And a co-assembly strategy of similar samples or multi-assemblers could further improve the assembly quality and the robustness of the findings.
3. Data and code availability. The raw sequencing data, fundamental information of samples, environmental factors for each sample, codes for processing data should be publicly available. Currently, there were no statements and it was difficult to

reproduce the results.

Minor suggestions:
Lines 114-115: The data volume for assembled contigs is relatively low compared to the raw sequencing data. Also the sequencing depth for the 150 samples is quietly different, the reads-rich one (DC1_1) reached 1.28 billion reads with 2.58 million contigs, while the reads-less one (DC3_8) only had 36.98 million reads with only 650 contigs. The assembly process of contigs was relatively poor with uneven distributions of contigs across samples, especially the low proportions of reads used for assembly.
Lines 131-133: Since most of the SGBs were recovered from active layer samples, the comparison of diversity based on this result between active layer and sub-active layer was biased.
Line 135. Explain “Tp”.
Lines 138-140. Same situation of viral genomes to the prokaryotic genomes, due to the uneven sequencing depths and poor assembly quality.
Line 198. Explain “Eh” here.
Lines 233-235. Lack of experimental flux of N_2O production.
Lines 326-327. This manuscript did not account the loss of carbon or nitrogen, and this discussion section needs more evidence.
Lines 393-394. Unclear description. If every 10 cm depth for one sample would get 150 samples for one drilling core of 15 m depth, and in total, 900 samples for six drilling cores, but only 150 samples got for six drilling cores in the manuscript.
Lines 470-471. The length of contigs for viral genomes was relatively lower. Please state the criterion selection.
Lines 478. The process of SGBs was not described.
Line 493. “analysed” to “analyze”.
Fig.1 The origin source of SGBs might be problematic, because the SGBs were derepeated across the six drilling cores, then the SGBs should come from multiple drilling cores rather than only one drilling core.

Reviewer #3

(Remarks to the Author)
The topic of this manuscript is very important and timely. Overall, the study design is scientifically sound at addressing the topic of alpine permafrost degradation. The dataset is interesting and I commend the authors for their dedication in obtaining the cores and their care in vertically stratifying them. However, the study limited to metagenomic and physicochemical soil analyses and is overall descriptive and provides limited mechanistic insights. Some additional, concerns below:

1) The strategy for MAG binning is unclear. Were co-assemblies used, if so, how were the samples grouped? This will potentially affect the interpretation of MAG recovery across layers.
2) The MAG analysis is a bit superficial. The MAGs could be compared in abundance across layers for example. This type of genome-resolved analysis is overall missing from the manuscript.
3) The viral portion of the manuscript is somewhat problematic and largely descriptive. Even correlating viral abundance vs. predicted host abundance across samples would be helpful. Do the detected viruses and their predicted hosts co-vary across layers or the permafrost degradation gradient?
4) The study only focuses on DNA viruses (since only metagenomics is performed). However, RNA viruses generally pose greater biosafety risks due to their higher mutation rates (e.g., influenza, HIV, SARS, COVID-19, Ebola, rabies, polio). It is troubling that RNA viruses, and this important caveat, are not addressed while framing the virus analysis in terms of biosafety hazards.
5) Proper negative controls are missing. As permafrost samples are generally extremely low in biomass, it is critical to have negative extraction controls (which are sequenced). This is especially true if whole genome amplification is used (as in this study).
6) Some lack of detail in how the SEM models were constructed and if sample nesting (i.e. samples from the same cores) was taken into consideration.
7) Line 399 “It was important to note that we also interpolated those missing or erroneous Tp data using machine learning algorithms.” – There is no methodology given for this.

Minor: Supplementary Fig 1 would be good to have in the main body.

Version 1:

Reviewer comments:

Reviewer #1

(Remarks to the Author)
The authors have made substantial revisions in response to the previous review comments. I find that all of my concerns and suggestions have been adequately addressed. The manuscript has been significantly improved in terms of clarity, rigor, and presentation. Therefore, I recommend that the manuscript be accepted for publication in its current form.

Reviewer #2

(Remarks to the Author)
The authors have well addressed my previous concerns.

Reviewer #3

(Remarks to the Author)
1) I would like to suggest that in future studies the authors do sequence negative controls despite insufficient material. It is still possible to make libraries with 'undetected' DNA in negative controls and sequence them (you treat it as if you have enough material), there are always some reads that come out of these negative (insufficient DNA) libraries, which can be used to verify the negative controls or to potentially remove some taxa from the downstream analysis.

2) I'm not sure its needed for this manuscript at this point (though would be nice for Nat Comms!), but since the authors did co-assemblies and individual assemblies for binning MAGs, a good (hybrid) strategy would be to use MAGs from both of these strategies and dereplicate them all together, then retain the dereplicated ones. Potentially some of the MAGs from the co-assemblies would be unique or higher quality compared to ones from individual assemblies (potentially from the lower abundance taxa). This would maximise the amount/quality of MAGs recovered without any sacrifice.

Open Access This Peer Review File is licensed under a Creative Commons Attribution 4.0 International License, which permits use, sharing, adaptation, distribution and reproduction in any medium or format, as long as you give appropriate credit to the original author(s) and the source, provide a link to the Creative Commons license, and indicate if changes were made.
In cases where reviewers are anonymous, credit should be given to 'Anonymous Referee' and the source.
The images or other third party material in this Peer Review File are included in the article’s Creative Commons license, unless indicated otherwise in a credit line to the material. If material is not included in the article’s Creative Commons license and your intended use is not permitted by statutory regulation or exceeds the permitted use, you will need to obtain permission directly from the copyright holder.
To view a copy of this license, visit https://creativecommons.org/licenses/by/4.0/

Response to the Reviewers' Comments

We sincerely appreciate the positive assessment of our work and insightful advice. We have made changes to the revised manuscript based on your constructive comments and suggestions. Our point-by-point responses to each comment and suggestion are as follows. Comments and suggestions are in black *italics*, our responses are in blue, and revisions to the manuscript are in dark red.

Response to Reviewer #1

This manuscript presents a large and carefully generated metagenomic dataset from alpine permafrost on the Qinghai-Tibet Plateau (QTP), including 501 MAGs, 28,008 viral genomes, and 43 million non-redundant genes derived from 150 samples collected from six 15-m-deep permafrost cores along a degradation gradient. Through systematic multi-layer sampling and explicit division of the active, frozen fringe, and frozen layers, this study substantially extends the vertical scope of permafrost microbiomes. The integrated analysis of microbiomes and environmental variables allows the authors to characterize depth- and degradation-related variations in the microbiome, and to link hydrothermal regimes, microbiome patterns, and carbon/nitrogen loss under permafrost degradation. Collectively, the work provides a valuable genomic resource and offers novel insights into the roles of biotic and abiotic factors in affecting biogeochemical processes and potential biosafety implications in degrading permafrost of QTP. However, I have some suggestions and comments about the current version of the manuscript, which I outline below.

[Response]: We sincerely thank you for your positive and precise summary of our study, and for recognizing the importance of our dataset and analyses. We are also grateful for your constructive suggestions and feedback, which have significantly improved the rigor and clarity of our manuscript. In light of your comments, we have carefully revised the text of the manuscript, and our point-by-point responses are detailed below.

My major concerns:

1. There is a strong bias in MAG recovery toward the active layer, with substantially fewer genomes reconstructed from deeper layers (frozen fringe and frozen layers). It should be explicitly discussed regarding its potential impact on conclusions about deeper layers. For example, some of the apparent contrasts between the active and frozen fringe/frozen layers may partly reflect differential assembly and binning efficiency rather than true biological absence or rarity. Clarifying this limitation will help readers interpret the depth-related patterns more cautiously.

[Response]: We sincerely thank the reviewer for this critical and insightful comment. Based on metagenomic analyses from six 15 m-depth drilling cores of alpine permafrost, we recovered a total of 501 metagenome-assembled genomes (MAGs), of which 441 were reconstructed from the active layer, whereas only 60 were recovered from the deeper layers (frozen fringe and frozen layers). This clearly shows a strong bias in MAG recovery toward the active layer, with substantially lower genome recovery from the deeper layers.

In our study, each sample was sequenced to at least 50 GB of raw data. The k-mer rarefaction curves at the sample level indicated that sequencing depth was sufficient to capture the sequence diversity (see **Fig. R1**). In addition, the data generated from all samples were used for the same assembly and binning procedures (see ***Metagenomic assembly and binning*** in the

Methods Section). Therefore, we are confident in confirming this bias to be majorly driven by intrinsic biological constraints of low-biomass samples in the deeper layers. These samples not only yield low amounts of recoverable DNA, but also limited non-redundant sequences, resulting in a lower number and shorter total length of contigs (Jansson & Taş, 2014[21]; Hammond *et al.*, 2015[83]). In addition, the potential DNA damage and fragmentation may have also constrained assembly continuity (Liang *et al.*, 2021[84]) resulting in the recovery of fewer MAGs in the deeper layers. This interpretation is further supported by the sequencing and assembly statistics provided in Supplementary Table 1.

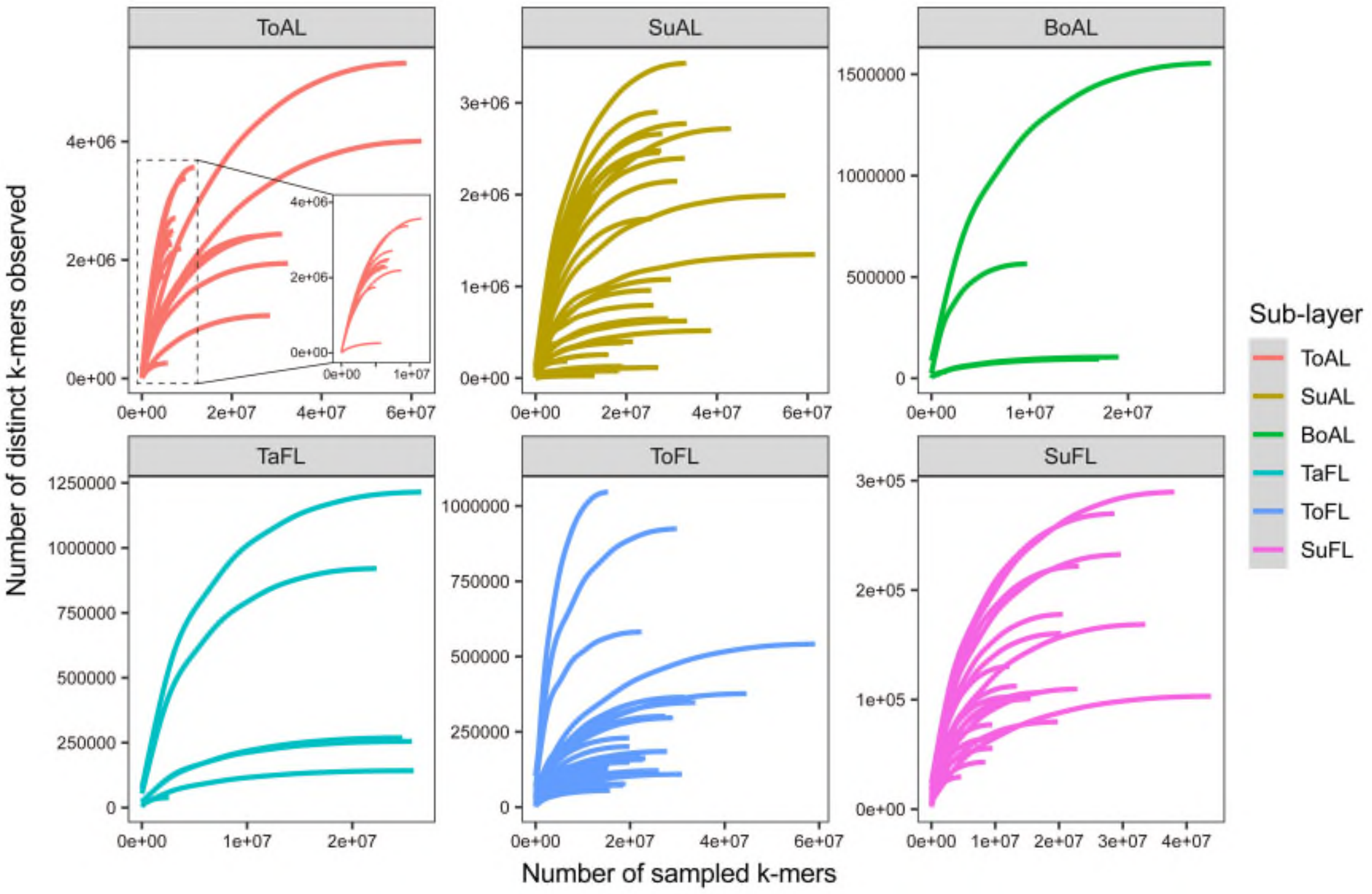

Fig. R1 | k-mer rarefaction curves for assessing metagenomic sequencing depth across sub-layers. Rarefaction curves were generated from quality-filtered metagenomic reads for each sample using 31-mers and a sampling step size of 1000. The x-axis represents the cumulative number of k-mers sampled, and the y-axis represents the cumulative number of unique k-mers observed. ToAL, top-active layer; SuAL, sub-active layer; BoAL, bottom-active layer; TaFL, table-frozen layer; ToFL, top-frozen layer; SuFL, sub-frozen layer.

Therefore, prokaryotic taxa uniquely adapted to the deeper layers, particularly those with unusual genomic features that hinder assembly and are present at extremely low abundance, may be missing from our catalogs or not represented because the recovered genomes do not meet our quality thresholds.

We have updated the ***Discussion Section*** to discuss this bias and its potential impact on our conclusions. Meanwhile, due to a similar concern regarding viral genomes raised by Reviewer #2, the recovery bias across layers is jointly discussed as follows:

Lines 371-386: "…… The majority of the recovered MAGs and viral genomes originated from

the active layer, with substantially fewer genomes reconstructed from the deeper layers. …… We believe these disparities are primarily driven by intrinsic biological constraints of some samples, especially low-biomass samples from the deeper layers. In these samples, low recoverable DNA and limited non-redundant sequences may reduce retention of clean reads and assembly efficiency, thereby resulting in fewer or shorter contigs[21, 83]. In addition, the potential DNA damage and fragmentation may have also constrained assembly continuity[84]. Consequently, both our SGB catalog and viral genome catalog, the reference databases used for diversity comparisons, are inherently biased toward the active layer. Prokaryotic taxa and viral lineages uniquely adapted to the deeper layers, particularly those possessing unusual genomic features that resist assembly and present at extremely low abundances, may be partially missing from our catalogs or not represented because the recovered genomes fail to meet our quality thresholds. ……"

References:

21. Jansson, J.K. & Taş, N. The microbial ecology of permafrost. *Nat. Rev. Microbiol.* **12**, 414-425 (2014).

83. Hammond, M., Homa, F., Andersson-Svahn, H., Ettema, T.J.G. & Joensson, H.N. Picodroplet partitioned whole genome amplification of low biomass samples preserves genomic diversity for metagenomic analysis. *Microbiome* **4**, 52 (2016).

84. Liang, R. et al. Genomic reconstruction of fossil and living microorganisms in ancient Siberian permafrost. *Microbiome* **9**, 110 (2021).

2. The high proportion of unclassified viral genomes (74%) is striking and merits explicit commentary. It would be helpful to discuss whether this primarily reflects true viral novelty in alpine permafrost or current limitations of reference databases. A brief comparison with Arctic permafrost viromes would provide valuable context.

[Response]: Thanks for your helpful suggestions. We recovered a total of 28,008 viral genomes, of which 7,250 could be taxonomically annotated. However, it is indeed striking that 74% of the viral genomes remained unclassified. We believe this pattern reflects both the true viral novelty in alpine permafrost and the limitations of current viral reference databases. To improve annotation coverage, we selected three common viral reference databases for testing, including Demovir, ProkaryoteViralRefSeq211-Merged, and the International Committee on Taxonomy of Viruses (ICTV). We ultimately used the annotation results generated by Demovir because it achieved the highest annotation rate (26%), whereas the results obtained from the other two databases showed extremely low annotation rates (both below 3%). Nevertheless, Demovir still has constrains, owing to the limited representation of viruses from permafrost samples in its reference database. Moreover, recent studies also reported that 83% (Emerson *et al.*, 2018[43]) and 85% (Trubl *et al.*, 2018[44]) of viral genomes in Arctic permafrost remained unclassified, respectively. These results indicate that a high unknown proportion of viruses is common in permafrost.

Accordingly, we have updated lines 337-345 in the ***Discussion Section*** to make it more explicit that the substantial unclassified viral genomes reflect both true novelty and current constraints of reference databases, and to add a brief comparison of our results with Arctic permafrost viruses.

Lines 337-345: "Notably, about 74% of the recovered viral genomes remained unclassified at the family level, indicating that a high proportion of viral lineages in alpine permafrost remains unresolved under current reference frameworks. This pattern likely reflects both true novelty of viral lineages in alpine permafrost and the limitations arising from the incompleteness of current reference databases. Viruses from permafrost remain underexplored, and currently available databases and classification tools often have limited ability to assign highly divergent viral sequences[72]. Consistent with this interpretation, previous studies of Arctic permafrost also reported similarly high proportions of unclassified viral genomes (83% and 85%), indicating that a large unknown fraction of viruses is common in permafrost[43, 44]."

References:

43. Emerson, J.B. et al. Host-linked soil viral ecology along a permafrost thaw gradient. *Nat. Microbiol.* **3**, 870-880 (2018).

44. Trubl, G. et al. Soil viruses are underexplored players in ecosystem carbon processing. *mSystems* **3**, 870-880 (2018).

72. Kieft, K. & Anantharaman, K. Virus genomics: what is being overlooked? *Curr. Opin. Virol.* **53** (2022).

3. Several central claims (e.g., "hydrothermal regimes as primary drivers", "microbiomes exert the largest total effect on POCD") currently rest on correlative modeling and should be presented with appropriate caution. Besides, while the SEMs propose plausible pathways, the language should be tempered to reflect their correlative nature, favoring terms like "associations" over "regulatory pathways" unless causal mechanisms are experimentally validated.

[Response]: Thank you for these important comments. The relevant statements have been revised throughout the ***Abstract***, ***Results***, and ***Discussion Sections*** as follows:

Lines 18-19: "Hydrothermal regimes emerged as the primary controls shaping microbiome distributions across the vertical profile and along the degradation gradient."

Lines 131-134: "Notably, the hydrothermal regime stood out as the predominant environmental variable modulating the SGBs Shannon index across the vertical profile, with permafrost water content (PWC) and Tp explaining 34.1% and 30.6% of the variance, respectively."

Lines 151-153: "Importantly, hydrothermal regime explained the largest proportion of variation in viral Shannon index (46.1%) throughout the vertical profile, followed by pH (45.8% of the variance explained)."

Lines 242-244: "The random forest model explained 70% of the variance in POCD and highlighted hydrothermal regime and the Shannon indices of microbiomes as the primary

controls on POCD variation across the vertical profile."

Lines 249-250: "…… uncovering clear pathways of biotic and abiotic factors regulating carbon/nitrogen stock."

Lines 254-256: "Interestingly, the degradation-affected microbiomes showed the strongest associations with POCD, emphasizing their important role in regulating carbon stock with the degradation."

Lines 399-400: "Hydrothermal regimes primarily regulated these variation patterns, leading to carbon/nitrogen loss during the degradation."

4. The manuscript refers to "carbon/nitrogen loss", "exacerbated carbon release", and "increased N_2O emissions", while the data are based on spatial comparisons among sites with different PDI values, measuring POCD/PTND and functional gene abundances/ratios ((nirK+nirS)/nosZ), without temporal data or direct flux measurements. Additionally, the increased (nirK+nirS)/nosZ ratio is presented as an indicator of N_2O emission potential. It is important to clarify in the text that this reflects a genetic capacity, not confirmed in situ activity, which would require complementary transcriptomic or isotopic data.

[Response]: Thanks for your insightful suggestions. In this study, we performed principal component analysis (PCA) on active layer thickness and mean annual ground temperature of each drilling site to yield a permafrost degradation index (PDI). These permafrost degradation indices for six drilling sites were ranked in ascending order to characterize a degradation sequence from S1 to S6 based on a methodology of space-for-time analysis, which is commonly used in permafrost studies (Patzner *et al.*, 2020; Wu *et al.*, 2021; Patzner *et al.*, 2022; Chen *et al.*, 2026). We then examined the relationships between PDI and permafrost organic carbon density (POCD), permafrost total nitrogen density (PTND), and the (*nirK* + *nirS*)/*nosZ* ratio, respectively. The observed declines in POCD and PTND with increasing PDI were used to infer carbon/nitrogen loss under permafrost degradation. Likewise, without direct flux measurements, the increased (*nirK* + *nirS*)/*nosZ* ratio was interpreted as an indicator of greater N_2O emission potential rather than confirmed *in situ* activity or direct N_2O emission.

The relevant statements have been revised in the ***Results*** and ***Discussion Sections***, and the ***Discussion Section*** also includes future research directions, as shown below.

Lines 231-233: "To assess the genetic potential related to N_2O emission, we investigated the ratio of nitrite reductase (*nirK* + *nirS*) to N_2O reductase (*nosZ*), a proxy widely used to indicate potential N_2O production[52, 53]."

Lines 329-336: "These findings indicated a heightened genetic capacity for nitrogen turnover, aligning with previous studies that associate thawing permafrost with increased N_2O emission potential[39, 52, 56]. …… However, while the increased (*nirK* + *nirS*)/*nosZ* ratio indicates an enhanced metabolic potential for N_2O emission, actual metabolic rate and *in situ* emission remain unconfirmed. Further studies should incorporate multiple methods including metatranscriptomic sequencing and flux measurement to further validate this pattern."

References:

Chen, S. et al. Divergent responses of bacterial communities to permafrost degradation and their associations with carbon across vertical profiles. *Adv. Sci.* **13**, e10516 (2026).

Patzner, M. et al. Iron mineral dissolution releases iron and associated organic carbon during permafrost thaw. *Nat. Commun.* **11**, 6329 (2020).

Patzner, M. et al. Seasonal fluctuations in iron cycling in thawing permafrost peatlands. *Environ. Sci. Technol.* **56**, 4620-4631 (2022).

Wu, M. et al. Reduced microbial stability in the active layer is associated with carbon loss under alpine permafrost degradation. *Proc. Natl. Acad. Sci. U.S.A.* **118**, e2025321118 (2021).

39. Marushchak, M.E. et al. Thawing Yedoma permafrost is a neglected nitrous oxide source. *Nat. Commun.* **12**, 7107 (2021).

52. Voigt, C. et al. Nitrous oxide emissions from permafrost-affected soils. *Nat. Rev. Earth Environ.* **1**, 420-434 (2020).

53. Yang, G. et al. Magnitude and pathways of increased nitrous oxide emissions from uplands following permafrost thaw. *Environ. Sci. Technol.* **52**, 9162-9169 (2018).

56. Waldrop, M.P. et al. Permafrost microbial communities and functional genes are structured by latitudinal and soil geochemical gradients. *ISME J.* **17**, 1224-1235 (2023).

5. The Discussion would benefit from an expanded exploration of potential drivers unique to alpine permafrost, such as substrate quality, microbial thermal adaptation, or redox history. In particular, the reported decrease in CAZyme diversity in the active layer along the degradation gradient, opposite to some previous findings, is a very interesting result. You attribute this pattern to shifts in substrate availability and quality toward more labile carbon. While this mechanism is plausible, no direct measurements of substrate quality are presented. I suggest explicitly framing this explanation as a hypothesis generated from gene profiles rather than a fully demonstrated mechanism, and using cautious wording in the Discussion.

[Response]: Thank you for highlighting this interesting result and providing constructive suggestions. While the substrate shift toward labile carbon is a possible explanation, it remains an inference derived from functional gene profiles rather than a mechanism supported by direct measurements. The ***Discussion Section*** has been revised accordingly to adopt more cautious language. In addition, we have expanded the ***Discussion Section*** to further discuss other potential drivers that may be particularly relevant to alpine permafrost. The corresponding revisions and additions are shown below.

Lines 309-317: "Interestingly, a significant decrease in CAZyme diversity in the active layer was observed with the degradation. This result was opposite to some previous findings[30, 67]. It may be because degradation-induced shifts in hydrothermal regimes not only release more readily available substrates but also impose stronger environmental filtering on microorganisms with genomic potential for complex carbon degradation[68]. These changes favor taxa that rely more on labile carbon and reduce the dominance of taxa carrying more diverse CAZyme

repertoires[69]. Together, these mechanisms may contribute to the observed decline in CAZyme diversity but warrant further validation through direct substrate characterization."

References:
30. Chen, Y. et al. Large-scale evidence for microbial response and associated carbon release after permafrost thaw. *Glob. Change Biol.* **27**, 3218-3229 (2021).
67. Pold, G. et al. Long-term warming alters carbohydrate degradation potential in temperate forest soils. *Appl. Environ. Microbiol.* **82**, 6518-6530 (2016).
68. Wu, L. et al. Permafrost thaw with warming reduces microbial metabolic capacities in subsurface soils. *Mol. Ecol.* **31**, 1403-1415 (2022).
69. Cuartero, J. et al. Increased carbon inputs alter soil microbial genetic potential for biogeochemical cycling in Arctic ecosystems. *Commun. Earth Environ.* **6** (2025).

6. The enrichment of viruses and virulence factors in the frozen fringe is intriguing. However, regarding biosafety (L348-353), the discussion remains speculative. I suggest moderating the language unless direct links to known pathogens or evidence of mobility for the detected ARGs/VFs are established, to maintain a precise and evidence-based tone. In addition, there is no direct evidence of viability or activity of these viruses or hosts, nor genomic context analyses showing ARGs/VFs on plasmids, phages, or integrative elements, nor comparisons with contemporary environmental communities.

[Response]: Thanks for your rigorous comments and suggestions. We apologize that the previous discussion of biosafety implications was not sufficiently cautious. Because our study was primarily intended to characterize microbial community structure and functional attributes using DNA metagenomic data, the current dataset supports only the detection of viral sequences and identification of ARGs and VFs at the genomic level. It is not sufficient to directly assess their mobility, viability, or biological activity, nor to establish direct links to known pathogens or resolve their genomic context. Given the limited amount and the long-term storage of the remaining samples, further validation by complementary analyses (e.g., metatranscriptomics and long-read metagenomics) was challenging at the current stage. Additionally, although comparison with contemporary environmental communities would indeed provide valuable context, it was difficult to perform in the present study. The primary reason lies in the scarcity of contemporary environmental virome datasets, particularly those from environments similar to our sampling sites.

In response to these concerns, the ***Discussion Section*** has been revised to adopt a more objective and evidence-based tone, and we acknowledging these constraints and incorporating them into the future directions. A related comment from Reviewer #3 regarding RNA viruses has also been taken into account, as reflected in the revised text below.

Lines 361-367: "……While our dataset characterizes the genomic reservoir of viruses, VFs, and ARGs, it does not provide direct evidence of mobility, viability, or activity, nor does it establish direct links to known pathogens or resolve their genomic context. …… Incorporating

metatranscriptomics and long-read metagenomic sequencing in future studies will also be important.”

7. The presentation of methods and supplementary materials should be improved to enhance reproducibility and transparency. The DNA extraction method cites a patent; a brief summary of key modifications would aid reproducibility. In addition, serval supplementary tables are currently unreadable due to formatting errors. These must be corrected and verified upon revision.

[Response]: Thank you for these constructive suggestions. The ***Methods*** and ***Supplementary Materials Sections*** have been improved to enhance reproducibility and transparency.

(1) DNA extraction: In the revised manuscript, we have summarized the key modifications of our DNA extraction protocol relative to a standard CTAB procedure. Relative to the conventional CTAB procedure, the main modifications were the use of SDS plus proteinase K instead of RIPA-type lysis and a longer incubation with preheated CTAB/NaCl. These modifications were introduced to improve cell disruption and DNA recovery from low-biomass samples.

We have clarified this procedure in the ***Methods Section*** as “DNA was extracted using a modified cetyltrimethylammonium bromide (CTAB) protocol for low-biomass permafrost samples (Chinese Patent No. ZL202210792689.0; Japanese Patent No. 7530612). Relative to a conventional CTAB procedure, the main modifications were the use of SDS plus proteinase K instead of RIPA-type lysis and a longer incubation with preheated CTAB/NaCl. Specifically, sample suspension after homogenization using a tissue homogenizer was centrifuged at 12,000 rpm for 5 minutes, followed by discarding the supernatant. The remaining substance was resuspended in Tris-EDTA (TE) buffer. For cell lysis, 0.5 mL of 10% SDS and 50 μL proteinase K (20 mg/mL) were added, and the mixture was incubated at 37 °C for 1 h. Subsequently, 1.5 mL of NaCl (5 mol/L) was added and mixed, followed by 1.5 mL of CTAB/NaCl solution preheated to 65 °C and incubation at 65 °C for 30 min. The lysate was extracted with phenol:chloroform:isopentanol (25:24:1) mixture, and the aqueous phase was used for DNA precipitation with isopropanol.” in lines 472-483.

(2) Supplementary tables: We apologize for the formatting issues. We have re-exported and reformatted all Supplementary Tables, verified completeness, readability (fonts, column widths, line breaks, and special characters), and double-checked that all tables open correctly in standard software. The corrected tables have been uploaded with the revised manuscript.

8. Language and data availability: The overall structure of the manuscript is clear and figures are well integrated, but the text contains numerous grammatical and stylistic issues (articles, prepositions, plural forms, awkward collocations, very long noun phrases). A thorough language edit by a fluent English speaker or professional service is recommended, especially for the complex sentences. Additionally, the manuscript currently lacks a dedicated Data

Availability statement. Based on open data science policies and fair principles, the sequencing data should be deposited in public repositories, and accession numbers and links should be provided to ensure transparency and reusability.

[Response]: Thanks for these helpful suggestions. We have thoroughly revised the manuscript, with particular attention to articles, prepositions, single/plural forms, complex sentence structures, tense consistency, precision and appropriateness of language, and the use of standardized terminology. It is worth noting that the manuscript was carefully revised by one of our co-authors, who is a fluent English speaker.

Regarding the ***Data*** A***vailability Section***, we apologize that this information was not sufficiently clear in the blinded manuscript. The sequencing data have been deposited in CNGBdb (https://db.cngb.org/). The ***Data Availability Section*** has been added into the revised manuscript. Because the current manuscript is under double-blind peer review, we have temporarily hidden the accession link for the sequencing data, as it contains information that could identify the authors. However, we have provided the statement of ***Data Availability*** again to the editor in the cover letter for availability review. If this manuscript is accepted, we will fully disclose the link to ensure transparency and reusability.

Data Availability

The sequencing data that support the findings of this study have been deposited into CNGB Sequence Archive (CNSA) of China National GeneBank DataBase (CNGBdb) with accession number CNP000XXXX (https://db.cngb.org/search/project/CNP000XXXX/). All the data have been deposited in China National GeneBank (CNGB), a non-profit, public-service-oriented organization in China. All relevant data are available from the authors. Source data are provided with this paper.

Other minor issues:

1. L29: "in the boundary" should be "at the boundary".

[Response]: Thanks! The phrase has been corrected as suggested.

2. L67: replace "Naturally" with "As expected".

[Response]: Done!

3. L73-74: "the diminished" should be "the diminishing", "the potential increases" should be "the potential increase".

[Response]: These issues have been corrected.

4. L133 and L393: phrases like "It was worth noting that…" or "It was worth stressing that…" could be streamlined to "Notably, …" or "Importantly, …" for more natural English.

[Response]: In accordance with your feedback, we have streamlined the phrase to "Notably" to ensure more natural English.

5. L175: "Candidates" is ambiguous in this context. "Detected" or "identified" is more precise for metagenomic data.

[Response]: Thank you! We have replaced "candidates" with "identified".

6. L200-201: "exhibited"? Should carefully check the tense throughout the manuscript.

[Response]: The tense has been corrected, and the use of tense has been carefully checked throughout the manuscript.

7. L205: avoid using subjective words such as 'obviously'.

[Response]: The subjective words (e.g., obviously and strikingly) have been removed throughout the manuscript.

8. L266: "across the vertical profile"?

[Response]: Thank you for this helpful suggestion. We have revised this phrase to "across the vertical profile" and carefully checked similar expressions throughout the manuscript to ensure the accurate and consistent use of articles.

9. L340: "upon the degradation"?

[Response]: We appreciate this suggestion. After careful review, we have retained the use of "during" to maintain consistency and accuracy.

10. L394 and L416-417: The manuscript states that 150 samples were collected from six 15 m cores, yet the Methods also mention "one sample was collected every 10 cm depth… yielding a total of 150 samples" and later "25 samples… were selected from 150 samples of each drilling core". The latter phrasing appears inconsistent with the earlier total of 150 samples and creates confusion about how many samples per core and per layer were actually sequenced.

[Response]: Thanks for pointing out this ambiguity. During field sampling, samples of each 15 m-depth drilling core were collected every 10 cm, yielding 150 samples (15 m / 0.1 m = 150). Consequently, a total of 900 samples were collected from the six drilling cores. We then selected 25 representative samples across the vertical profile (covering the three main-layers and six sub-layers) per drilling core, resulting in 150 samples (6 cores × 25 samples) which were used for metagenomic sequencing and physicochemical analyses. The relevant revisions in the ***Methods Section*** are provided below.

Lines 426-427: "During field sampling, samples from each drilling core were collected every 10 cm, yielding 150 samples per core and 900 samples in total."

Lines 461-463: "We then selected 25 representative samples from the aforementioned layers of each drilling core, resulting in 150 samples which were used for metagenomic sequencing and physicochemical analyses."

11. L413: "Furtherly" is not common use in standard academic English.

[Response]: "Furtherly" has been replaced with "Moreover" to adhere to standard academic English.

12. L491: "to test for differences"?

[Response]: We have replaced "calculate" with "evaluate" for greater accuracy.

13. Please standardize terminology such as "main (-) layers" vs "main layers", and consider using "degradation gradient" rather than "degraded gradient".

[Response]: Thanks! Terminology has been standardized throughout the manuscript.

14. Fig1: What is the sample size for each layer? For example, plots showing Shannon index comparisons and regressions, sample sizes should be provided.

[Response]: Very good suggestion! We have included the sample size (*n*) for each main-layer and sub-layer in the legends of all relevant figures (Figs. 2-6, S3, S6-S9, S12, S13, and S16).

15. The number of supplementary figures appear excessive; I suggest moving some of them to the extended data figure.

[Response]: Thank you for this helpful suggestion. We checked both the submission system and the Author Guidelines and found that the journal does not provide an "Extended Data Figure" category. Additionally, we carefully reviewed each supplementary figure and confirmed that all of them provide essential evidence and relevant details to support our results. Therefore, we respectfully request to retain all of the current supplementary figures in the Supplementary Information file.

16. A few references show minor formatting inconsistencies (journal name abbreviation, hyphenation, etc.). Please harmonize them according to the journal's reference style.

[Response]: Reference formatting has been checked and harmonized according to the journal's reference style.

Overall, this study fully advances the field and will be of interest to researchers in microbial ecology, cryospheric science, and environmental genomics. The English is generally clear but contains numerous grammatical and stylistic issues, and some causal and risk-related statements appear overstated. Substantial revisions are still needed, particularly regarding data interpretation, statistical nuance, and the accessibility of the supplementary files. I recommend acceptance after major revisions, on the condition that the authors adequately address the points above.

[Response]: We sincerely thank you for your encouraging assessment and the recommendation

for acceptance after major revision. Your advice has been taken seriously, and substantial revisions have been made to improve the manuscript. Specifically, we have thoroughly polished the English text to refine sentence structure, ensure tense consistency, standardize terminology, and address other language-related issues throughout the manuscript. Relevant interpretations were also revised to improve precision and statistical nuance, and the statements related to causal relationships and potential risks were carefully tempered. In addition, we have further reviewed the presentation of the supplementary materials to improve their accessibility and clarity. As detailed in our point-by-point responses above, we believe these revisions have substantially strengthened the robustness and readability of the study.

Response to Reviewer #2

This manuscript demonstrated a deep metagenomic analysis of 150 samples across six 15-m permafrost cores in QTP area, and found that degradation specifically reduces microbial diversity in the active layer while enriching viruses and virulence factors in the frozen fringe. This kind of change was primarily driven by hydrothermal regimes. Notably, these hydrothermal and microbiome attributes jointly regulate carbon/nitrogen loss in response to future warming permafrost systems. The manuscript was well-written and the results could well-support their findings, I only have several concerns for the clarification of key concepts and data interpretations.

[Response]: Thanks for your insightful evaluation and comments of our work! We are pleased that you found the manuscript was well-written and that the results supported our conclusions. Your professional comments have guided us in performing a more rigorous revision, which has fully enhanced the quality of our manuscript. We have provided detailed clarifications regarding the key concepts and data interpretations. Please find our point-by-point responses to your specific concerns below.

Major comments:

1. The authors used active thickness and ground temperature to calculate a degradation index to classify the gradient of permafrost soils of six drilling cores. However, shallow active layer thickness does not necessarily indicate severe permafrost degradation. Because permafrost degradation is fundamentally defined by a persistent thickening trend of the active layer and rising ground temperatures, rather than absolute depth. Additional evidence to show the degradation tendency of these six drilling cores may strengthen the findings, such as monitory data for active layer thickness changes, ground plant community changes or ground temperature changes in continuous years.

[Response]: Thanks for your constructive comments. We fully agree with you that permafrost degradation is primarily reflected by a combined trend of thickening active layer and rising ground temperature, which has been widely reported in previous studies (Hjort *et al*., 2022; Hu *et al*., 2024). Accordingly, permafrost degradation cannot be adequately reflected by changes in a single parameter, particularly ground temperature. For example, S2 (drilling site 2) exhibits higher mean annual ground temperature (MAGT) but shallower active layer thickness (ALT) compared to S3 (Fig. S1). However, this does not necessarily indicate S2 is more severely degraded than S3. To address this point, we obtained the permafrost degradation index (PDI) for each drilling site based on a principal component analysis combining ALT and MAGT so that the degradation was characterized objectively. These permafrost degradation indices for six drilling sites were ranked in ascending order to characterize a degradation sequence from S1 to S6 based on a methodology of space-for-time analysis, which is commonly used in permafrost studies (Patzner *et al.*, 2020; Wu *et al.*, 2021[14]; Patzner *et al.*, 2022; Chen *et al.*,

2026[46]).

Furthermore, we take your suggestion regarding the necessity of continuous monitoring seriously. To further validate our current findings in the future, we have been continuously monitoring ALT and MAGT at each drilling site in recent years to accurately track the temporal variation patterns of permafrost. Meanwhile, regular observations and sampling of plant community characteristics and permafrost microbiomes are also conducted.

To ensure the meaning and algorithm of the PDI are clearly communicated to readers, we have further stated explicitly in both the ***Introduction*** (lines 94-98) and the ***Methods Sections*** (lines 447-450).

Lines 94-98: "To characterize the extent of degradation, we obtained the permafrost degradation index (PDI) for each drilling site based on a principal component analysis (PCA) combining active layer thickness (ALT) and mean annual ground temperature (MAGT) at 15 m depth[46], and ranked this index in ascending order to represent a degradation sequence from drilling site 1 (S1) to S6."

Lines 447-450: "Subsequently, we observed ALT and MAGT at a depth of 15 m among six drilling sites, respectively. These two key parameters reflecting permafrost stability were analyzed using PCA, yielding the first principal component represented as the PDI[46]."

References:

Hjort, J. et al. Impacts of permafrost degradation on infrastructure. *Nat. Rev. Earth. Environ.* **3**, 24-38 (2022).

Hu, G. et al. Large variability in permafrost degradation over the Northern Hemisphere. *Catena.* **246**, 108440 (2024).

Patzner, M. et al. Iron mineral dissolution releases iron and associated organic carbon during permafrost thaw. *Nat. Commun.* **11**, 6329 (2020).

Patzner, M. et al. Seasonal fluctuations in iron cycling in thawing permafrost peatlands. *Environ. Sci. Technol.* **56**, 4620-4631 (2022).

14. Wu, M. et al. Reduced microbial stability in the active layer is associated with carbon loss under alpine permafrost degradation. *Proc. Natl. Acad. Sci. U.S.A.* **118**, e2025321118 (2021).

46. Chen, S. et al. Divergent responses of bacterial communities to permafrost degradation and their associations with carbon across vertical profiles. *Adv. Sci.* **13**, e10516 (2026).

2. The sequencing depth for each sample was relatively huge as 50 Gb of sequencing data could result in solid results. But according to the assembly results, the assembly quality and efficiency might be poor due to the highly fragmented reads of pair-end 100 bps sequencing technology. A basic assessment of sequencing depth for these samples is needed to evaluate the diversity profillings. And a co-assembly strategy of similar samples or multi-assemblers could further improve the assembly quality and the robustness of the findings.

[Response]: Thank you for these insightful comments and suggestions. To evaluate the potential influence of sequencing depth on downstream analyses, we supplemented our

assessment of sequencing depth by adding k-mer rarefaction curves at the sample level (see **Fig. R1**). Results indicated that the sequencing depth was sufficient to capture the existing sequence diversity within the samples. Hence, the relatively low assembly output in some samples is unlikely to be due to insufficient sequencing depth. In addition, while the relatively short read length of the PE100 strategy may have imposed some limitations on assembly continuity, all samples were sequenced using the same strategy, and we therefore do not consider read length alone to be the primary explanation for the pronounced differences in assembly performance across layers.

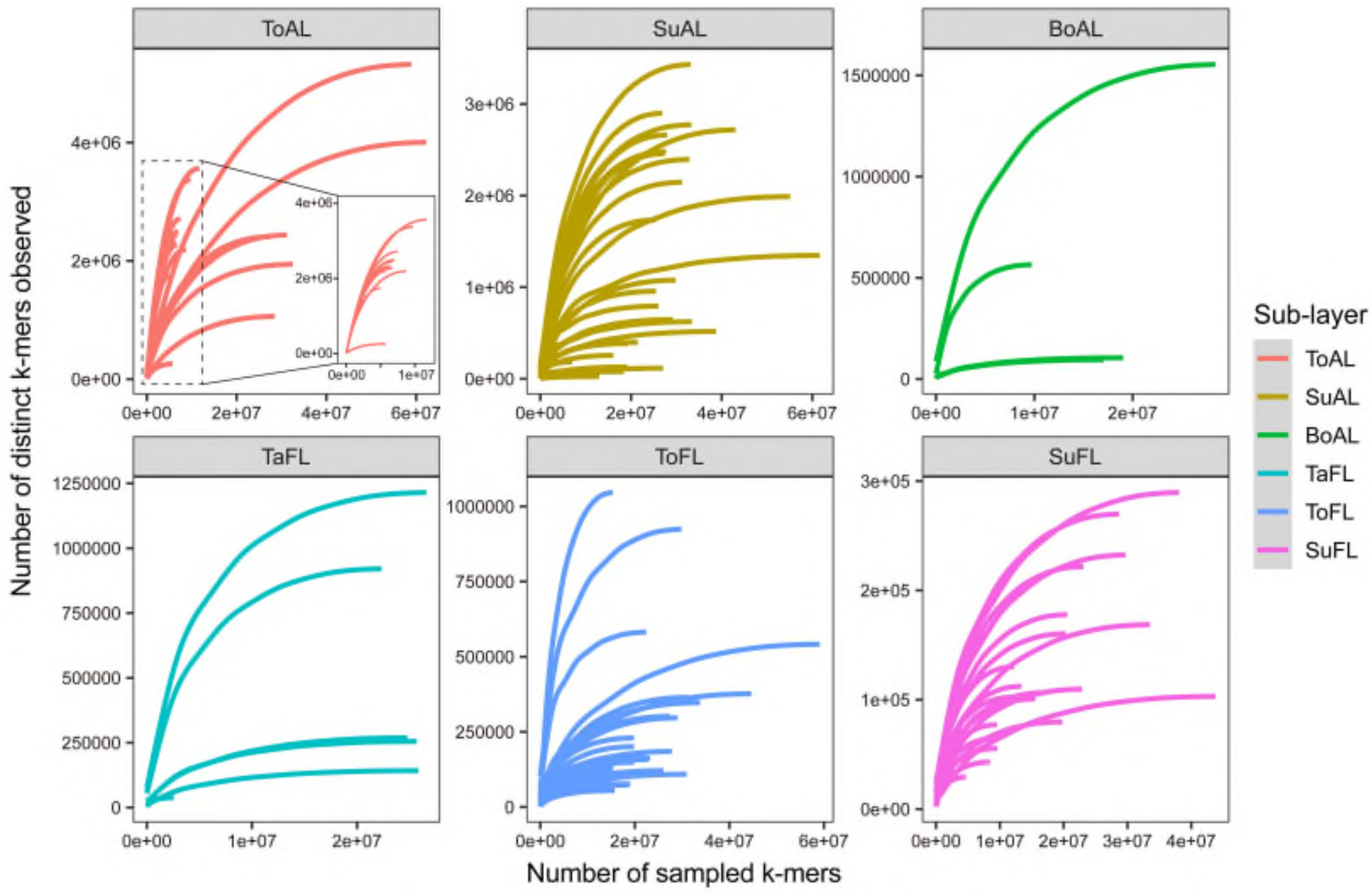

Fig. R1 | k-mer rarefaction curves for assessing metagenomic sequencing depth across sub-layers. Rarefaction curves were generated from quality-filtered metagenomic reads for each sample using 31-mers and a sampling step size of 1000. The x-axis represents the cumulative number of k-mers sampled, and the y-axis represents the cumulative number of unique k-mers observed. ToAL, top-active layer; SuAL, sub-active layer; BoAL, bottom-active layer; TaFL, table-frozen layer; ToFL, top-frozen layer; SuFL, sub-frozen layer.

Nevertheless, we believe the marked variation in contig number and assembly yield across layers reflects an inherent challenge in environmental metagenomic studies, where samples span a gradient from high-biomass to extremely low-biomass. In the low-biomass samples from the deeper layers, not only was there the limited amount of recoverable DNA and non-redundant sequences, but there were potential DNA damage and fragmentation that may constrain assembly continuity (Jansson & Taş, 2014; Hammond *et al.*, 2015; Liang *et al.*, 2021). Consequently, despite sufficient sequencing depth, these samples yielded fewer and shorter contigs, and a relatively low proportion of reads was incorporated into assembled contigs.

In addition, we employed single-sample assembly strategy using MEGAHIT (v1.2.9) in this

manuscript, which allowed us to maximize the retention of sample-specific genetic information. Nevertheless, in an attempt to improve genome completeness and assembly continuity, we rigorously tested a co-assembly strategy with MEGAHIT (v1.2.9) on the basis of your suggestion. Samples from the same sub-layer across the six drilling cores were initially grouped together for co-assembly. Because the data volume of some groups exceeded our available computational capacity, these groups were further partitioned into smaller sub-groups with comparable data volumes (see **Table R1**). In this way, we sought to minimize potential bias in the interpretation of metagenome-assembled genomes (MAGs) recovery across sub-layers. After the partitioning, a total of 25 sub-groups were used for co-assembly.

Although contig assembly quality under the co-assembly strategy indeed improved as expected (Co-assembly: Max. length 515 kb, N50 2.78±1.21 kb; Single-sample assembly: Max. length 472 kb, N50 1.26±0.77 kb; see **Table R2**), the performance of MAG binning decreased dramatically compared to the single-sample assembly. Specifically, the co-assembly yielded 172 MAGs representing 155 species-level genome bins (SGBs) while the single-sample assemblies recovered 501 MAGs representing 282 SGBs (see **Table R3**). We assume this is primarily due to chimeric contigs which probably interfere with the MAG binning procedure in some samples. Therefore, we did not retain the co-assembly results but still kept the results of single-sample assembly in the revised manuscript. It was mainly because the single-sample strategy provided a more reliable basis for interpreting MAG recovery across layers, particularly in the deeper layers. We have also revised the ***Methods Section*** to clarify this workflow in lines 496-499.

Lines 496-499: "Reads that passed the quality control assessment were assembled using a single-sample assembly strategy with MEGAHIT (v1.2.9)[91], retaining contigs exceeding 500 bp in size for subsequent analysis. Metagenomic binning was performed independently for each sample using MetaWRAP (v1.3)[92]."

References:

Hammond, M., Homa, F., Andersson-Svahn, H., Ettema, T.J.G. & Joensson, H.N. Picodroplet partitioned whole genome amplification of low biomass samples preserves genomic diversity for metagenomic analysis. *Microbiome* **4**, 52 (2016).

Jansson, J.K. & Taş, N. The microbial ecology of permafrost. *Nat. Rev. Microbiol.* **12**, 414-425 (2014).

Liang, R. et al. Genomic reconstruction of fossil and living microorganisms in ancient Siberian permafrost. *Microbiome* **9**, 110 (2021).

91. Uritskiy, G.V., DiRuggiero, J. & Taylor, J. MetaWRAP-a flexible pipeline for genome-resolved metagenomic data analysis. *Microbiome* **6**, 158 (2018).

92. Li, D., Liu, C.M., Luo, R., Sadakane, K. & Lam, T.W. MEGAHIT: an ultra-fast single-node solution for large and complex metagenomics assembly via succinct de Bruijn graph. *Bioinformatics* **31**, 1674-1676 (2015).

The results of the co-assembly experiments are provided below for your reference:

Table R1. Grouping strategy of co-assembling.

Sample ID	Sub-group	Sub-layer (Group)	Sample ID	Sub-group	Sub-layer (Group)	Sample ID	Sub-group	Sub-layer (Group)	Sample ID	Sub-group	Sub-layer (Group)	Sample ID	Sub-group	Sub-layer (Group)	Sample ID	Sub-group	Sub-layer (Group)
DC1_1	G_01	ToAL	DC3_4	G_05	SuAL	DC4_4	G_09	SuAL	DC2_15	G_13	ToFL	DC4_19	G_17	ToFL	DC5_19	G_21	ToFL
DC3_2			DC4_6			DC4_5			DC2_9			DC5_18			DC2_24	G_22	SuFL
DC4_1			DC5_10			DC4_7			DC1_12			DC5_20			DC3_21		
DC4_3			DC6_12			DC5_11			DC1_14			DC3_13	G_18		DC3_22		
DC5_3			DC6_14			DC5_4			DC2_8	G_14		DC3_20			DC4_24		
DC6_2			DC6_8			DC1_6	G_10	BoAL	DC3_18			DC4_18			DC2_21		
DC1_2	G_02		DC1_5	G_06		DC2_7			DC4_14			DC5_17			DC2_22		
DC2_2			DC2_6			DC3_10			DC5_15			DC6_19			DC3_24	G_23	
DC3_1			DC3_6			DC4_11			DC6_18			DC1_16			DC4_23		
DC4_2			DC3_9			DC5_12			DC1_13			DC1_8	G_19		DC5_21		
DC5_2			DC5_8			DC6_15			DC1_20	G_15		DC2_19			DC5_22		
DC6_3			DC6_10			DC1_6.1	G_11	TaFL	DC2_14			DC3_16			DC6_21		
DC1_3	G_03		DC3_5	G_07		DC2_7.1			DC3_14			DC4_16			DC6_22		
DC2_1			DC3_8			DC3_10.1			DC3_19			DC6_17			DC4_21	G_24	
DC2_3			DC4_9			DC4_11.1			DC5_13			DC1_10			DC4_22		
DC3_3			DC5_5			DC5_12.1			DC1_18			DC1_9	G_20		DC5_24		
DC5_1			DC6_4			DC6_15.1			DC2_10	G_16		DC2_13			DC6_24		
DC6_1			DC6_7			DC1_7	G_12	ToFL	DC4_13			DC3_17			DC1_21		
DC1_4	G_04	SuAL	DC3_7	G_08		DC2_18			DC4_17			DC5_14			DC1_24		
DC2_4			DC5_6			DC4_12			DC4_20			DC6_20			DC3_23	G_25	
DC4_10			DC6_11			DC4_15			DC1_15			DC1_11			DC5_23		
DC4_8			DC6_13			DC5_16			DC1_17			DC1_19	G_21		DC6_23		
DC5_7			DC6_5			DC6_16			DC2_16	G_17		DC2_17			DC1_22		
DC5_9			DC6_9			DC2_11	G_13		DC3_12			DC2_20			DC1_23		
DC6_6			DC2_5	G_09		DC2_12			DC3_15			DC3_11			DC2_23		

ToAL, top-active layer; SuAL, sub-active layer; BoAL, bottom-active layer; TaFL, table-frozen layer; ToFL, top-frozen layer; SuFL, sub-frozen layer.

Table R2. Basic metrics of co-assemblies.

Sub-group	Number of Contigs	Max. Length	N50	GC (%)
G_01	3,792,706	338,816	1,232	48.82
G_02	4,178,866	194,624	1,213	49.41
G_03	3,432,320	376,730	1,205	50.36
G_04	2,031,818	515,875	2,676	45.38
G_05	2,394,521	355,436	2,429	47.06
G_06	2,058,364	167,031	2,558	47.27
G_07	674,156	103,123	2,895	46.8
G_08	984,316	71,899	2,750	44.59
G_09	3,283,548	397,694	2,300	46.05
G_10	726,890	227,755	2,752	45.08
G_11	771,592	180,091	3,588	50.36
G_12	33,427	128,571	3,249	46.43
G_13	19,422	50,386	2,781	46.6
G_14	584,460	81,060	2,820	48.11
G_15	23,549	41,102	3,127	48.68
G_16	270,850	134,405	4,309	51.24
G_17	52,473	139,297	5,995	57.23
G_18	57,026	165,097	3,539	47.54
G_19	88,481	90,634	2,945	47.97
G_20	54,423	277,103	3,282	50.51
G_21	33,353	104,000	6,255	55.3
G_22	25,796	93,172	2,185	48.07
G_23	31,866	196,305	4,310	52.92
G_24	35,138	140,905	3,080	49.69
G_25	32,256	85,692	1,851	45.61

Table R3. MAG binning summary of co-assemblies.

Sub-group	Low Quality	Medium Quality	High Quality
G_01	367	39	4
G_02	353	28	5
G_03	282	47	8
G_04	419	5	1
G_05	546	3	0
G_06	444	9	0
G_07	193	0	0
G_08	216	1	0
G_09	922	13	1
G_10	192	2	0
G_11	227	4	0
G_12	6	0	0
G_13	0	0	0
G_14	111	1	0
G_15	0	0	0
G_16	45	1	0
G_17	23	0	0
G_18	23	0	0
G_19	11	0	0
G_20	9	0	0
G_21	25	0	0
G_22	2	0	0
G_23	9	0	0
G_24	17	0	0
G_25	9	0	0
**Total**	**4451**	**153**	**19**

3. Data and code availability. The raw sequencing data, fundamental information of samples, environmental factors for each sample, codes for processing data should be publicly available. Currently, there were no statements and it was difficult to reproduce the results.

[Response]: Thanks for pointing this out. We apologize that the availability of the underlying data and materials was not sufficiently clear in the blinded manuscript.

To ensure reproducibility, the sequencing data and fundamental information of samples have been deposited in the CNGBdb (https://db.cngb.org/). The data of environmental variables have been provided in the Supplementary Table 11, and the source data of corresponding figures have also been uploaded to the submission system.

The ***Data Availability*** and ***Code Availability Sections*** have been added to the revised manuscript. Since author information is contained in the accession link for sequencing data in the ***Data Availability Section*** of the current manuscript under double-blind peer review, the accession number has to be temporarily replaced with "CNP000XXXX". However, we have provided the link again to the editor in the cover letter for availability review. If this manuscript is accepted, we will fully disclose the link to ensure transparency and reusability. In addition, the statement of source data has also been added to the ***Data Availability Section***. These sections are provided below.

Data Availability

The sequencing data that support the findings of this study have been deposited into CNGB Sequence Archive (CNSA) of China National GeneBank DataBase (CNGBdb) with accession number CNP000XXXX (https://db.cngb.org/search/project/CNP000XXXX/). All the data have been deposited in China National GeneBank (CNGB), a non-profit, public-service-oriented organization in China. All relevant data are available from the authors. Source data are provided with this paper.

Code Availability

No new code was created in this study.

Minor suggestions:

Lines 114-115: The data volume for assembled contigs is relatively low compared to the raw sequencing data. Also the sequencing depth for the 150 samples is quietly different, the reads-rich one (DC1_1) reached 1.28 billion reads with 2.58 million contigs, while the reads-less one (DC3_8) only had 36.98 million reads with only 650 contigs. The assembly process of contigs was relatively poor with uneven distributions of contigs across samples, especially the low proportions of reads used for assembly.

[Response]: Thank you for pointing out these data issues. We agree that assembly output was relatively low compared with the raw sequencing data and that assembly performance varied substantially among samples. Although at least 50 GB of raw sequencing data was generated for each sample, the retained clean reads after quality control ranged from only 36.98 million to 1.28 billion, while the number of assembled contigs ranged from only 650 to 2.58 million.

This disparity likely reflects an inherent challenge in environmental metagenomic studies, where samples span a gradient from low-biomass to high-biomass. The low-biomass samples not only yield low amounts of recoverable DNA, but also limited non-redundant sequences, resulting in a lower number and shorter total length of contigs. In addition, the potential DNA damage and fragmentation may have also constrained assembly continuity. Consequently, despite generally sufficient sequencing depth (see **Fig. R1**), these samples may incorporate a relatively low proportion of reads into assembly and yield fewer contigs. We have further clarified these issues in the ***Discussion Section***, as follows:

Lines 371-386: "…… The majority of the recovered MAGs and viral genomes originated from

the active layer, with substantially fewer genomes reconstructed from the deeper layers. In addition, despite the large amount of raw data generated for each sample, only a relatively small fraction of reads was incorporated into assembled contigs, and assembly output was quite different among samples. We believe these disparities are primarily driven by intrinsic biological constraints of some samples, especially low-biomass samples from the deeper layers. In these samples, low recoverable DNA and limited non-redundant sequences may reduce retention of clean reads and assembly efficiency, thereby resulting in fewer or shorter contigs[21, 83]. In addition, the potential DNA damage and fragmentation may have also constrained assembly continuity[84]. Consequently, both our SGB catalog and viral genome catalog, the reference databases used for diversity comparisons, are inherently biased toward the active layer. Prokaryotic taxa and viral lineages uniquely adapted to the deeper layers, particularly those possessing unusual genomic features that resist assembly and present at extremely low abundances, may be partially missing from our catalogs or not represented because the recovered genomes fail to meet our quality thresholds. ……"

References:

21. Jansson, J.K. & Taş, N. The microbial ecology of permafrost. *Nat. Rev. Microbiol.* **12**, 414-425 (2014).

83. Hammond, M., Homa, F., Andersson-Svahn, H., Ettema, T.J.G. & Joensson, H.N. Picodroplet partitioned whole genome amplification of low biomass samples preserves genomic diversity for metagenomic analysis. *Microbiome* **4**, 52 (2016).

84. Liang, R. et al. Genomic reconstruction of fossil and living microorganisms in ancient Siberian permafrost. *Microbiome* **9**, 110 (2021).

Lines 131-133: Since most of the SGBs were recovered from active layer samples, the comparison of diversity based on this result between active layer and sub-active layer was biased.

[Response]: Thanks for this important point. In our results, 254 of the 282 recovered SGBs originated from samples in the active layer, where there is significant bias. It may influence comparisons of SGB diversity across layers.

We are confident in confirming this bias to be driven by intrinsic biological constraints of samples in the deeper layers, including low biomass and potential DNA damage or fragmentation. To minimize the potential influence of this recovery bias on comparisons of SGB diversity across layers, we performed abundance profiling by recruiting reads from all samples to the complete catalog of recovered SGBs. Thus, after an SGB was incorporated into the catalog, its relative abundance could be quantified throughout all samples, including those from the deeper layers in which genome recovery was more limited.

Accordingly, we have updated the ***Discussion Section*** to acknowledge this bias and to clarify that the abundance profiling based on read recruitment to the full SGB catalog partially reduces the impact of uneven genome recovery, as follows:

Lines 381-388: "…… Consequently, both our SGB catalog and viral genome catalog, the reference databases used for diversity comparisons, are inherently biased toward the active layer. ….... Nonetheless, our abundance profiling provides a more quantitative perspective for the recovered prokaryotes and viruses, based on read recruitment to the dereplicated SGBs and viral Operational Taxonomic Units (vOTUs). ……"

Line 135. Explain "Tp".

[Response]: We apologize for any lack of clarity. "Tp" refers to the abbreviation of permafrost temperature. We have given the full name at its first occurrence in line 128, and thereafter used this abbreviation consistently throughout the main text of the manuscript.

Lines 138-140. Same situation of viral genomes to the prokaryotic genomes, due to the uneven sequencing depths and poor assembly quality.

[Response]: Thank you for this important concern. In this study, recovery of 28,008 viral genomes was similarly biased toward the active layer, with 82.82% of these genomes originating from active layer samples. In fact, each sample was sequenced to at least 50 GB of raw data. The k-mer rarefaction curves at the sample level further indicated that sequencing depth was sufficient to capture the sequence diversity (see **Fig. R1**). Since both viral contig identification and MAG reconstruction were based on the same metagenomic assembly output, the bias in genome recovery is unlikely to be due to insufficient sequencing depth. This bias likely reflects the intrinsic biological constraints of low-biomass samples in the deeper layers. These samples not only yield low amounts of recoverable DNA, but also contain limited non-redundant sequences, resulting in a lower number and shorter total length of contigs. In addition, the potential DNA damage and fragmentation may have also constrained assembly continuity, thereby contributing to the recovery of fewer viral contigs in samples from the deeper layers.
We have updated the ***Discussion Section*** to discuss this bias and its potential impact on our conclusions. Meanwhile, due to a similar concern regarding MAGs raised by Reviewer #1, the recovery bias across layers is jointly discussed, as follows:
Lines 372-386: "…… The majority of the recovered MAGs and viral genomes originated from the active layer, with substantially fewer genomes reconstructed from the deeper layers. …… We believe these disparities are primarily driven by intrinsic biological constraints of some samples, especially low-biomass samples from the deeper layers. In these samples, low recoverable DNA and limited non-redundant sequences may reduce retention of clean reads and assembly efficiency, thereby resulting in fewer or shorter contigs[21, 83]. In addition, the potential DNA damage and fragmentation may have also constrained assembly continuity[84]. Consequently, both our SGB catalog and viral genome catalog, the reference databases used for diversity comparisons, are inherently biased toward the active layer. Prokaryotic taxa and viral lineages uniquely adapted to the deeper layers, particularly those possessing unusual genomic features that resist assembly and present at extremely low abundances, may be partially missing

from our catalogs or not represented because the recovered genomes fail to meet our quality thresholds. ……"

References:

21. Jansson, J.K. & Taş, N. The microbial ecology of permafrost. *Nat. Rev. Microbiol.* **12**, 414-425 (2014).

83. Hammond, M., Homa, F., Andersson-Svahn, H., Ettema, T.J.G. & Joensson, H.N. Picodroplet partitioned whole genome amplification of low biomass samples preserves genomic diversity for metagenomic analysis. *Microbiome* **4**, 52 (2016).

84. Liang, R. et al. Genomic reconstruction of fossil and living microorganisms in ancient Siberian permafrost. *Microbiome* **9**, 110 (2021).

Line 198. Explain "Eh" here.

[Response]: We apologize for the lack of statement about "Eh". It refers to the abbreviation of redox potential. We have given the full name at its first occurrence in line 197, and thereafter used this abbreviation consistently throughout the main text of the manuscript.

Lines 233-235. Lack of experimental flux of N2O production.

[Response]: Thanks for this comment. Our result about N_2O emission was potential inference based on the ratio of nitrite reductase genes (*nirK* + *nirS*) to N_2O reductase genes (*nosZ*), which has been widely used as a genomic indicator of the potential balance between N_2O production and reduction (Voigt *et al.*, 2020[52]; Yang *et al.*, 2018[53]). However, N_2O flux is not measured in situ and will be monitored in the future. We have revised the ***Results*** and ***Discussion Sections***, as follows:

Lines 231-233: "To assess the genetic potential related to N_2O emission, we investigated the ratio of nitrite reductase (*nirK* + *nirS*) to N_2O reductase (*nosZ*), a proxy widely used to indicate potential N_2O production[52, 53]."

Lines 327-336: "Besides, as a key genomic proxy of N_2O production potential[52, 53], (*nirK* + *nirS*)/*nosZ* in the active and frozen fringe layers increased distinctly with the degradation. These findings indicated a heightened genetic capacity for nitrogen turnover, aligning with previous studies that associate thawing permafrost with increased N_2O emission potential[39, 52, 56]. …… However, while the increased (*nirK* + *nirS*)/*nosZ* ratio indicates an enhanced metabolic potential for N_2O emission, actual metabolic rate and *in situ* emission remain unconfirmed. Further studies should incorporate multiple methods including metatranscriptomic sequencing and flux measurement to further validate this pattern."

References:

39. Marushchak, M.E. et al. Thawing Yedoma permafrost is a neglected nitrous oxide source. *Nat. Commun.* **12**, 7107 (2021).

52. Voigt, C. et al. Nitrous oxide emissions from permafrost-affected soils. *Nat. Rev. Earth Environ.* **1**, 420-434 (2020).

53. Yang, G. et al. Magnitude and pathways of increased nitrous oxide emissions from uplands following permafrost thaw. *Environ. Sci. Technol.* **52**, 9162-9169 (2018).
56. Waldrop, M.P. et al. Permafrost microbial communities and functional genes are structured by latitudinal and soil geochemical gradients. *ISME J.* **17**, 1224-1235 (2023).

Lines 326-327. This manuscript did not account the loss of carbon or nitrogen, and this discussion section needs more evidence.

[Response]: Thank you very much for pointing this out. In this study, we performed PCA on ALT and MAGT of each drilling site to obtain a PDI. These permafrost degradation indices for six drilling sites were ranked in ascending order to characterize a degradation sequence from S1 to S6 based on a methodology of space-for-time analysis. We then examined the linear relationships between PDI and permafrost organic carbon density (POCD) and permafrost total nitrogen density (PTND), respectively. The observed decreases in POCD and PTND with increasing PDI were used to infer carbon/nitrogen loss under permafrost degradation. In addition, it was estimated that each 10% increase in PDI relative to its observed range was associated with declines of 5.5% in POCD and 5.9% in PTND in the active layer.

To provide more evidence, we have cited other studies and added quantitative estimates in the ***Discussion Section***, as follows:

Lines 317-320: "Meanwhile, significant declines were observed in POCD and PTND in the active layer with the degradation, in accordance with other findings[14, 70]. Based on linear regression models, each 10% increase in PDI relative to its observed range was associated with estimated declines of 5.5% in POCD and 5.9% in PTND."

References:

14. Wu, M. et al. Reduced microbial stability in the active layer is associated with carbon loss under alpine permafrost degradation. *Proc. Natl. Acad. Sci. U.S.A.* **118**, e2025321118 (2021).
70. Tan, Q., Jia, Y. & Wang, G. Decoupling of soil nitrogen and phosphorus dynamics along a temperature gradient on the Qinghai-Tibetan Plateau. *Geoderma* **396**, 115084 (2021).

Lines 393-394. Unclear description. If every 10 cm depth for one sample would get 150 samples for one drilling core of 15 m depth, and in total, 900 samples for six drilling cores, but only 150 samples got for six drilling cores in the manuscript.

[Response]: Sorry for the unclear description. During field sampling, samples of each 15 m-depth drilling core were collected every 10 cm, yielding 150 samples (15 m / 0.1 m = 150). Consequently, a total of 900 samples were collected from the six drilling cores. We then selected 25 representative samples across the vertical profile (covering the three main-layers and six sub-layers) per drilling core, resulting in 150 samples (6 cores × 25 samples) which were used for metagenomic sequencing and physicochemical analyses. The relevant revisions in the ***Methods Section*** are provided below.

Lines 426-427: "During field sampling, samples from each drilling core were collected every

10 cm, yielding 150 samples per core and 900 samples in total."

Lines 461-463: "We then selected 25 representative samples from the aforementioned layers of each drilling core, resulting in 150 samples which were used for metagenomic sequencing and physicochemical analyses."

Lines 470-471. The length of contigs for viral genomes was relatively lower. Please state the criterion selection.

[Response]: Thanks for your concern. In our workflow, all assembled contigs were first screened with VirSorter2 (v2.2.3), and predicted viral contigs were then further evaluated with CheckV (v1.0.1). We retained contigs encoding at least one virus-specific gene and longer than 2 kbp, as described in CheckV for the minimum length of viral genomes (Nayfach *et al.*, 2021[109]). The use of this length threshold can reduce noise from very short and highly fragmented contigs while still capturing a broad spectrum of viral sequences for dereplication and annotation.

We have provided additional details to make this criterion more explicit in the ***Methods Section***, as follows:

Lines 527-531: "The potential viral contigs were evaluated subsequently using CheckV (v1.0.1)[109]. We retained contigs encoding at least one virus-specific gene and longer than 2 kbp. These contigs were identified as viral genomes. The use of this length threshold can reduce noise from very short and highly fragmented contigs while still capturing a broad spectrum of viral sequences for dereplication and annotation."

Reference:

109. Nayfach S. et al. A genomic catalog of Earth's microbiomes. *Nat. Biotechnol.* **39**, 499-509 (2021).

Lines 478. The process of SGBs was not described.

[Response]: We apologize for this issue. In our study, all medium- and high-quality MAGs were dereplicated using dRep (v3.4.2) with a species-level threshold of 95% average nucleotide identity (ANI) and a minimum alignment fraction of 30% (-sa 0.95, -nc 0.30). The representative genome selected by dRep (v3.4.2) from each cluster was defined as an SGB and included in the non-redundant genome catalog used for downstream taxonomic classification, functional annotation, phylogenetic analysis, and abundance mapping.

We have revised the ***Methods Section*** in lines 502-505: "To enable genome-resolved analyses at SGB level, MAGs were clustered into SGBs using dRep (v3.4.2, parameters: -sa 0.95 -nc 0.30)[94] at a 95% ANI threshold[95]. The representative genome selected from each cluster was defined as an SGB."

References:

94. Olm, M.R., Brown, C.T., Brooks, B. & Banfield, J.F. dRep: a tool for fast and accurate genomic comparisons that enables improved genome recovery from metagenomes through de-

replication. *ISME J.* **11**, 2864-2868 (2017).
95. Almeida, A. et al. A unified catalog of 204,938 reference genomes from the human gut microbiome. *Nat. Biotechnol.* **39**, 105-114 (2021).

Line 493. "analysed" to "analyze".

[Response]: Thanks! We have corrected "analysed" to "analyze".

Fig.1 The origin source of SGBs might be problematic, because the SGBs were derepeated across the six drilling cores, then the SGBs should come from multiple drilling cores rather than only one drilling core.

[Response]: Thanks for your careful attention. In the present study, SGBs were defined by dereplicating MAGs recovered across six drilling cores. Therefore, a given SGB may include MAGs derived from one or multiple cores. In the phylogenetic tree of Fig. 1A, each SGB is shown only once by its representative genome, which should not be interpreted as indicating that the corresponding SGBs originated from only one drilling core. Instead, the adjacent pie chart shows the proportion of MAGs from different drilling cores within that SGB. For example, if an SGB contains MAGs from DC1, DC2, and DC4, the pie chart uses different colors to indicate the proportion of MAGs from each drilling core.

We have modified the figure legend accordingly. In addition, the original Fig. 1 has been renumbered as Fig. 2 in the revised manuscript following the suggestion of Reviewer #3. The revised ***Fig. 2 Legend*** has been provided below.

Lines 868-871: "The colored ring indicates the phylum level taxonomic classification of each SGB. The adjacent pie charts show the relative proportions of MAGs from different drilling cores within each SGB. The outer bar graphs summarize the total number of MAGs associated with each main-layer."

Response to Reviewer #3

The topic of this manuscript is very important and timely. Overall, the study design is scientifically sound at addressing the topic of alpine permafrost degradation. The dataset is interesting and I commend the authors for their dedication in obtaining the cores and their care in vertically stratifying them. However, the study limited to metagenomic and physicochemical soil analyses and is overall descriptive and provides limited mechanistic insights. Some additional, concerns below:

[Response]: We sincerely thank you for the recognition of the importance and timeliness of our study, as well as the appreciation for dedication in obtaining the stratified permafrost cores down to 15 m-depth and affirmation of our dataset. We also appreciate your thoughtful comments regarding the limited mechanistic insights between environmental variables and microbiome patterns.

By integrating published literature, we have revised and expanded the ***Discussion Section*** to further strengthen the mechanistic interpretation of the microbiome patterns observed across the vertical profile and along the degradation gradient. We hope these revisions provide a more comprehensive framework for interpreting the observed microbiome patterns. For detailed modifications, please review our point-by-point responses listed below.

Lines 283-288: "Specifically, low temperatures reduce membrane fluidity and restrict the diffusion of metabolic substrates, thereby suppressing cell division and enzyme activity[57]. Meanwhile, the higher pH values typically found in deeper layers (frozen fringe and frozen layers) may alter the adsorption-desorption balance of key elements, aggravate nutrient limitation[56, 58] and ultimately contribute to the lower diversity observed in the deeper layers[29, 59]."

Lines 304-309: "Previous studies have shown rapid shifts in community composition with fast-growing taxa becoming more prevalent as permafrost transitions from the frozen to thawed states[33, 66]. In contrast, the rising temperature generally constrains the growth and metabolic ability of cold-adapted microorganisms by altering their physiological activity and impairing cellular functions[14, 40], helping to explain decrease in much of microbial diversity observed in the active layer under the degradation."

References:

14. Wu, M. et al. Reduced microbial stability in the active layer is associated with carbon loss under alpine permafrost degradation. *Proc. Natl. Acad. Sci. U.S.A.* **118**, e2025321118 (2021).

29. Kang, L. et al. Metagenomic insights into microbial community structure and metabolism in alpine permafrost on the Tibetan Plateau. *Nat. Commun.* **15**, 5920 (2024).

33. Mackelprang, R. et al. Metagenomic analysis of a permafrost microbial community reveals a rapid response to thaw. *Nature* **480**, 368-371 (2011).

40. Ernakovich, J.G. et al. Microbiome assembly in thawing permafrost and its feedbacks to climate. *Glob. Change Biol.* **28**, 5007-5026 (2022).

56. Waldrop, M.P. et al. Permafrost microbial communities and functional genes are structured by latitudinal and soil geochemical gradients. *ISME J.* **17**, 1224-1235 (2023).
57. D'Amico, S., Collins, T., Marx, J.C., Feller, G. & Gerday, C. Psychrophilic microorganisms: challenges for life. *Embo Reports* **7**, 385-389 (2006).
58. Gustafsson, J.P., Mwamila, L.B. & Kergoat, K. The pH dependence of phosphate sorption and desorption in Swedish agricultural soils. *Geoderma* **189**, 304-311 (2012).
59.Zhou, X. et al. Global analysis of soil bacterial genera and diversity in response to pH. *Soil Biol. Biochem.* **198**, 109552 (2024).
66. Scheel, M. et al. Abrupt permafrost thaw triggers activity of copiotrophs and microbiome predators. *FEMS Microbiol. Ecol.* **99** (2023).

1) The strategy for MAG binning is unclear. Were co-assemblies used, if so, how were the samples grouped? This will potentially affect the interpretation of MAG recovery across layers.

[Response]: We apologize for the unclear description. The metagenome-assembled genomes (MAGs) reported in this manuscript were reconstructed using single-sample assembly and binning, and no co-assembly was used.

Nevertheless, in an attempt to improve genome completeness and assembly continuity, we rigorously tested a co-assembly strategy with MEGAHIT (v1.2.9) following your suggestion. Samples from the same sub-layer across the six drilling cores were initially grouped together for co-assembly. Because the data volume of some groups exceeded our available computational capacity, these groups were further partitioned into smaller sub-groups with comparable data volumes (see **Table R1**). This design was intended to minimize potential bias in the interpretation of metagenome-assembled genomes (MAGs) recovery across sub-layers. After this partitioning, a total of 25 sub-groups were used for co-assembly.

Although the co-assembly strategy improved contig assembly quality as expected (Co-assembly: Max. length 515 kb, N50 2.78±1.21 kb; Single-sample assembly: Max. length 472 kb, N50 1.26±0.77 kb; see **Table R2**), MAG binning performance was substantially decreased compared with the single-sample assembly strategy. Specifically, the co-assembly yielded 172 MAGs while the single-sample assemblies recovered 501 MAGs (see **Table R3**). We infer that this is primarily due to chimeric contigs which probably interfere with the MAG binning procedure in some samples. Therefore, we did not retain the co-assembly results but still kept the results of single-sample assembly in the revised manuscript. This was mainly because the single-sample strategy provided a more reliable basis for interpreting MAG recovery across layers, particularly in the deeper layers. We have also revised the ***Methods Section*** to clarify this workflow in lines 496-499.

Lines 496-499: "Reads that passed the quality control assessment were assembled using a single-sample assembly strategy with MEGAHIT (v1.2.9)[91], retaining contigs exceeding 500 bp in size for subsequent analysis. Metagenomic binning was performed independently for each sample using MetaWRAP (v1.3)[92]."

References:

91. Uritskiy, G.V., DiRuggiero, J. & Taylor, J. MetaWRAP-a flexible pipeline for genome-resolved metagenomic data analysis. *Microbiome* **6**, 158 (2018).

92. Li, D., Liu, C.M., Luo, R., Sadakane, K. & Lam, T.W. MEGAHIT: an ultra-fast single-node solution for large and complex metagenomics assembly via succinct de Bruijn graph. *Bioinformatics* **31**, 1674-1676 (2015).

The results of the co-assembly experiments are provided below for your reference:

Table R1. Grouping strategy of co-assembling.

Sample ID	Sub-group	Sub-layer (Group)	Sample ID	Sub-group	Sub-layer (Group)	Sample ID	Sub-group	Sub-layer (Group)	Sample ID	Sub-group	Sub-layer (Group)	Sample ID	Sub-group	Sub-layer (Group)	Sample ID	Sub-group	Sub-layer (Group)
DC1_1	G_01	ToAL	DC3_4	G_05	SuAL	DC4_4	G_09	SuAL	DC2_15	G_13	ToFL	DC4_19	G_17	ToFL	DC5_19	G_21	ToFL
DC3_2			DC4_6			DC4_5			DC2_9			DC5_18			DC2_24	G_22	SuFL
DC4_1			DC5_10			DC4_7			DC1_12			DC5_20			DC3_21		
DC4_3			DC6_12			DC5_11			DC1_14			DC3_13	G_18		DC3_22		
DC5_3			DC6_14			DC5_4			DC2_8	G_14		DC3_20			DC4_24		
DC6_2			DC6_8			DC1_6	G_10	BoAL	DC3_18			DC4_18			DC2_21		
DC1_2	G_02		DC1_5	G_06		DC2_7			DC4_14			DC5_17			DC2_22		
DC2_2			DC2_6			DC3_10			DC5_15			DC6_19			DC3_24	G_23	
DC3_1			DC3_6			DC4_11			DC6_18			DC1_16			DC4_23		
DC4_2			DC3_9			DC5_12			DC1_13			DC1_8	G_19		DC5_21		
DC5_2			DC5_8			DC6_15			DC1_20	G_15		DC2_19			DC5_22		
DC6_3			DC6_10			DC1_6.1	G_11	TaFL	DC2_14			DC3_16			DC6_21		
DC1_3	G_03		DC3_5	G_07		DC2_7.1			DC3_14			DC4_16			DC6_22		
DC2_1			DC3_8			DC3_10.1			DC3_19			DC6_17			DC4_21	G_24	
DC2_3			DC4_9			DC4_11.1			DC5_13			DC1_10			DC4_22		
DC3_3			DC5_5			DC5_12.1			DC1_18			DC1_9	G_20		DC5_24		
DC5_1			DC6_4			DC6_15.1			DC2_10	G_16		DC2_13			DC6_24		
DC6_1			DC6_7			DC1_7	G_12	ToFL	DC4_13			DC3_17			DC1_21		
DC1_4	G_04	SuAL	DC3_7	G_08		DC2_18			DC4_17			DC5_14			DC1_24		
DC2_4			DC5_6			DC4_12			DC4_20			DC6_20			DC3_23	G_25	
DC4_10			DC6_11			DC4_15			DC1_15			DC1_11	G_21		DC5_23		
DC4_8			DC6_13			DC5_16			DC1_17			DC1_19			DC6_23		
DC5_7			DC6_5			DC6_16			DC2_16	G_17		DC2_17			DC1_22		
DC5_9			DC6_9			DC2_11	G_13		DC3_12			DC2_20			DC1_23		
DC6_6			DC2_5	G_09		DC2_12			DC3_15			DC3_11			DC2_23		

ToAL, top-active layer; SuAL, sub-active layer; BoAL, bottom-active layer; TaFL, table-frozen layer; ToFL, top-frozen layer; SuFL, sub-frozen layer.

Table R2. Basic metrics of co-assemblies.

Sub-group	Number of Contigs	Max. Length	N50	GC (%)
G_01	3,792,706	338,816	1,232	48.82
G_02	4,178,866	194,624	1,213	49.41
G_03	3,432,320	376,730	1,205	50.36
G_04	2,031,818	515,875	2,676	45.38
G_05	2,394,521	355,436	2,429	47.06
G_06	2,058,364	167,031	2,558	47.27
G_07	674,156	103,123	2,895	46.8
G_08	984,316	71,899	2,750	44.59
G_09	3,283,548	397,694	2,300	46.05
G_10	726,890	227,755	2,752	45.08
G_11	771,592	180,091	3,588	50.36
G_12	33,427	128,571	3,249	46.43
G_13	19,422	50,386	2,781	46.6
G_14	584,460	81,060	2,820	48.11
G_15	23,549	41,102	3,127	48.68
G_16	270,850	134,405	4,309	51.24
G_17	52,473	139,297	5,995	57.23
G_18	57,026	165,097	3,539	47.54
G_19	88,481	90,634	2,945	47.97
G_20	54,423	277,103	3,282	50.51
G_21	33,353	104,000	6,255	55.3
G_22	25,796	93,172	2,185	48.07
G_23	31,866	196,305	4,310	52.92
G_24	35,138	140,905	3,080	49.69
G_25	32,256	85,692	1,851	45.61

Table R3. MAG binning summary of co-assemblies.

Sub-group	Low Quality	Medium Quality	High Quality
G_01	367	39	4
G_02	353	28	5
G_03	282	47	8
G_04	419	5	1
G_05	546	3	0
G_06	444	9	0
G_07	193	0	0
G_08	216	1	0
G_09	922	13	1
G_10	192	2	0
G_11	227	4	0
G_12	6	0	0
G_13	0	0	0
G_14	111	1	0
G_15	0	0	0
G_16	45	1	0
G_17	23	0	0
G_18	23	0	0
G_19	11	0	0
G_20	9	0	0
G_21	25	0	0
G_22	2	0	0
G_23	9	0	0
G_24	17	0	0
G_25	9	0	0
**Total**	**4451**	**153**	**19**

2) The MAG analysis is a bit superficial. The MAGs could be compared in abundance across layers for example. This type of genome-resolved analysis is overall missing from the manuscript.

[Response]: Thanks for your constructive comment. Because SGBs were more representative, the MAG-level analyses were superficial. In particular, MAGs were dereplicated with a species-level threshold of 95% ANI and a minimum alignment fraction of 30%. The representative genome from each cluster was defined as an SGB, thereby avoiding repeated counting of the same or highly similar taxa. In addition, the unified SGB catalog provided a non-redundant reference framework, which prevented multi-mapping of reads to highly similar genomes and

ensured accurate abundance profiling across all samples. Moreover, we reduced redundancy among highly similar genomes by clustering MAGs into SGBs. This dereplication likely mitigated noise and reduced the burden of multiple testing, thereby helping improve the statistical power. We therefore performed comprehensive genome-resolved analyses at the SGB level, including taxonomic assignment, phylogenetic reconstruction, functional annotation, abundance profiling, and the patterns of SGBs across the vertical profile as well as their responses to permafrost degradation.

We would like to respectfully draw your attention to the following sections, where these SGB-based results are presented in detail:

1) Fig. 2A displays the phylogenetic tree of representative MAGs from all 282 SGBs. This provides a comprehensive overview of the distribution and abundance patterns of the recovered genomes across both the vertical profile and the degradation gradient.

2) Fig. 5C specifically analyzes the nine SGBs enriched in the frozen fringe and frozen layers, illustrating their specific carbon and nitrogen cycling genes. This provides direct genomic evidence for the metabolic potential of microbes in these layers. For example, SGB_86_1 (*Stenotrophomonas* sp.) presents comprehensive metabolic potential for carbohydrate degradation (cellulose and hemicellulose), while SGB_109_1 (*Moraxella_A cinereus* sp.) is involved in nitrogen cycling (nitrate reduction, nitric oxide reduction, and nitrite reduction to ammonia).

3) Fig. S2 presents the prevalence of SGBs across the main-layers, further supporting the depth-related distribution patterns.

4) In the ***Results Section***, we also discuss the abundance dynamics of specific SGBs across the degradation gradient. For example, we highlight the ammonia-oxidizing archaeon *Nitrososphaera* sp. (SGB_227_2), which shows a significant positive association with permafrost degradation index (PDI), directly linking a specific genome-resolved taxon to a key biogeochemical process (N_2O production) under permafrost degradation (Fig. S15).

3) The viral portion of the manuscript is somewhat problematic and largely descriptive. Even correlating viral abundance vs. predicted host abundance across samples would be helpful. Do the detected viruses and their predicted hosts co-vary across layers or the permafrost degradation gradient?

[Response]: Thanks for your thoughtful comments and valuable suggestions. We fully agree that exploring the co-variation between detected viruses and their predicted hosts would provide significant mechanistic insights. To address this point, we attempted an exploratory abundance-correlation analysis using the limited set of matched virus-host pairs available in our dataset, including 39 viral Operational Taxonomic Units (vOTUs) and 4 predicted bacterial host species. Unfortunately, none of these pairs showed a strong correlation (see **Fig. R2A**). Panel B further indicated that PDI explained only a limited interaction of the variation in virus-host co-variation, with extremely low R^2 values across the tested pairs (see **Fig. R2B**).

However, these analyses are currently limited in their interpretability. In the present study, host assignment relied primarily on VirHostMatcher-Net, which predicts putative hosts based on similarity to reference genomes in public databases rather than direct linkage to the specific microbial taxa recovered from the same samples. In addition, the number of matched virus-host pairs was small. As a result, the current dataset does not provide a sufficiently robust basis for firm conclusions about virus-host co-variation across layers or along the permafrost degradation gradient.

A more robust test of virus-host co-variation will require improved recovery of sample-specific host genomes and more direct host-linking approaches, such as CRISPR spacer matching, prophage-based linkage, or proximity-ligation methods. Genome-resolved viral analyses, such as viral life-history strategies, virus-host network structure, and auxiliary metabolic genes are valuable directions for future work. These aspects are currently being investigated and will be reported thoroughly in subsequent studies.

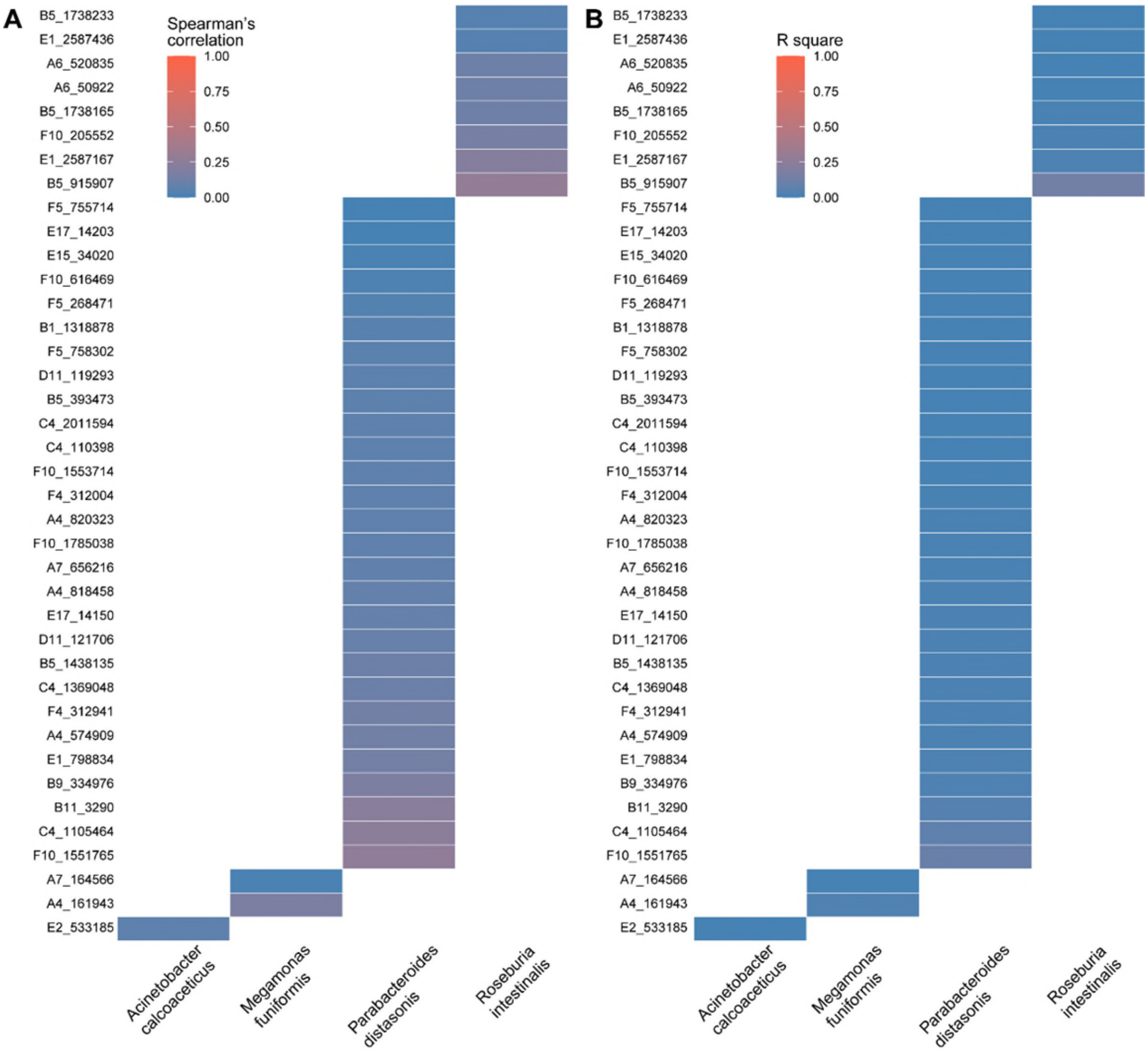

Fig. R2 | Viral–host abundance relationships. (A) Spearman's correlations between the abundances of 39 matched vOTUs and their 4 predicted bacterial host species across all samples. **(B)** Heatmap summarizing the R^2 values from multiple regression models evaluating whether PDI modulates the relationship between viral and host abundances. Warmer colors indicate stronger

correlations or greater explanatory power. PDI, permafrost degradation index.

4) The study only focuses on DNA viruses (since only metagenomics is performed). However, RNA viruses generally pose greater biosafety risks due to their higher mutation rates (e.g., influenza, HIV, SARS, COVID-19, Ebola, rabies, polio). It is troubling that RNA viruses, and this important caveat, are not addressed while framing the virus analysis in terms of biosafety hazards.

[Response]: Thank you for raising this important point. Our present study was primarily intended to characterize microbial community structure and functional attributes using metagenomic data. Therefore, this dataset supports only the detection of DNA viruses. In response to this comment, we have added statements to the ***Discussion Section*** to clarify that our virus-related interpretations are limited to the DNA viruses and there is still one-sidedness for a comprehensive assessment of potential biosafety risks in permafrost. In the future, it is essential to combine with RNA viruses due to higher mutation rates and greater potential risks. The added text reads as follows:

Lines 349-366: "However, it is worth emphasizing that our metagenomic data supports only the detection of DNA viruses. …… In the future, it is essential to combine with RNA viruses due to higher mutation rates and greater potential risks for comprehensive assessments of potential biosafety risks in permafrost."

5) Proper negative controls are missing. As permafrost samples are generally extremely low in biomass, it is critical to have negative extraction controls (which are sequenced). This is especially true if whole genome amplification is used (as in this study).

[Response]: We sincerely thank you for this critical point. Due to the potential trace amounts of contaminant DNA, we fully acknowledge that it is essential to consider negative controls when performing whole-genome amplification (WGA) for low-biomass permafrost samples.

To examine potential contamination, we set negative controls (blanks without sample material) that were conducted parallelly with samples in the processes of DNA extraction and WGA, respectively. Following the amplification, we performed polymerase chain reaction of 16S rRNA gene on all negative controls. All of them tested negative (representative results are shown in **Fig. R3**), indicating no detectable prokaryotic DNA contamination. Therefore, metagenomic library preparation and sequencing of these controls were not involved subsequently.

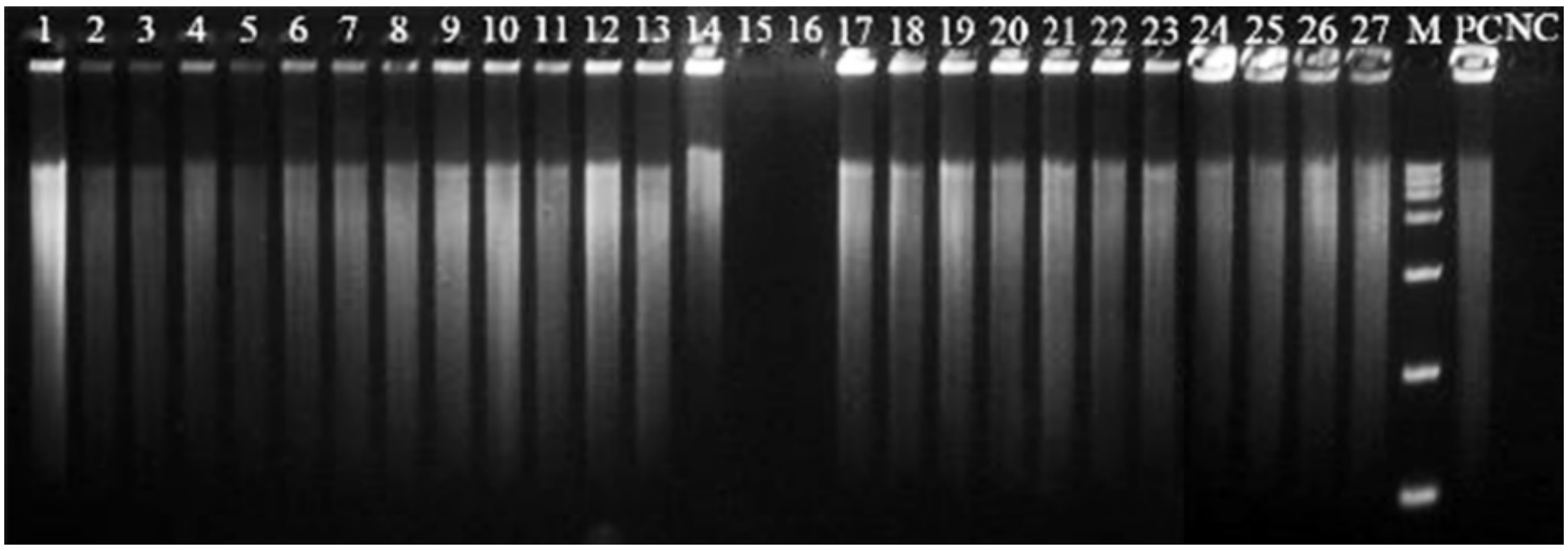

Fig. R3 | Agarose gel electrophoresis of PCR results. Lanes 1-27, DNAs from representative sample; lane M, DNA ladder (250 bp, 1,000 bp, 2,500 bp, 5,000 bp, 5,500 bp, 6,000 bp, and 15,000 bp); lane PC, positive control; lane NC, negative control.

Accordingly, we have added detailed descriptions in the ***Methods Section***, as follows:

Lines 486-490: "To monitor potential contamination during DNA extraction and WGA, negative controls were included and processed parallelly with the real samples. Following the amplification, all negative controls were tested by 16S rRNA gene PCR. All of them tested negative, indicating no detectable prokaryotic DNA contamination. Therefore, these negative controls were not subjected to metagenomic library preparation and sequencing."

6) Some lack of detail in how the SEM models were constructed and if sample nesting (i.e., samples from the same cores) was taken into consideration.

[Response]: Thanks for these important comments. In this study, the samples originated from multiple profile layers within cores from six drilling sites, thus sample nesting was inherent in the construction of the structural equation model (SEM). To evaluate the nesting effect, we first calculated intraclass correlation coefficients (ICC) using SPSS 27.0. The nesting effect (also known as the design effect, Deff) was calculated using the formula [Deff = 1 + (m − 1) × ICC] (m represents the average cluster size). For the sample data used in SEM1 construction (Fig. S17B), m was 25, ICC was 0.005, and the yielding Deff was 1.120. For SEM2 (Fig. S18B) and SEM3 (Fig. S19B), m was 9.167 and ICC was negative (-0.113), and the corresponding Deff was 1.000. Obviously, all Deff values < 2 that indicate the ignorable nesting effect (Muthén & Satorra, 1995[119]).

Each SEM was constructed by AMOS 24.0 after a priori hypothesis regarding the relationships among permafrost degradation parameter, environmental variables, microbiomes, and carbon/nitrogen stock. Finally, the model fit was evaluated using multiple indices, including the ratio of chi-square to degrees of freedom (χ^2/df), the root mean square error of approximation (RMSEA), the comparative fit index (CFI), and the p-value of the Bollen-Stine bootstrap test. The model was considered to be acceptable with $0 \leqslant \chi^2/df \leqslant 2$, RMSEA < 0.05, CFI > 0.9, and $p > 0.1$.

We have added these details of the model construction into the "***Statistical analysis***" in the

Methods Section, as follows:

Lines 559-573: "Considering that samples originated from multiple profile layers within cores from six drilling sites, sample nesting was subsistent in the construction of the SEM. To evaluate the nesting effect, we first calculated intraclass correlation coefficients (ICC) using SPSS 27.0. The nesting effect (also known as the design effect, Deff) was calculated using the formula [Deff = 1 + (m − 1) × ICC], where m represents the average cluster size. For the sample data used in SEM1 construction (Fig. S17B), m was 25, ICC was 0.005, and the yielding Deff was 1.120. For SEM2 (Fig. S18B) and SEM3 (Fig. S19B), m was 9.167 and ICC was negative (-0.113), and the corresponding Deff was 1.000. Obviously, all Deff values < 2 that indicate the ignorable nesting effect[119]. Each SEM was constructed by AMOS 24.0 after a priori hypothesis regarding the relationships among permafrost degradation parameter, environmental variables, microbiomes, and carbon/nitrogen stock. Finally, the model fit was evaluated using multiple indices, including the ratio of chi-square to degrees of freedom (χ^2/df), the root means square error of approximation (RMSEA), the comparative fit index (CFI), and the *p*-value of the Bollen-Stine bootstrap test. The model was considered to be acceptable with $0 \leqslant \chi^2/\text{df} \leqslant 2$, RMSEA < 0.05, CFI > 0.9, and $p > 0.1$."

Reference:

119. Muthén, B. & Satorra, A. Complex sample data in structural equation modeling. *Sociol. Methodol*. **25**, 267 (1995).

7) Line 399 "It was important to note that we also interpolated those missing or erroneous Tp data using machine learning algorithms." – There is no methodology given for this.

[Response]: We apologize for the insufficient detail on the interpolation methods. To address those missing or erroneous data of Tp, we applied machine learning algorithms using the scikit-learn library in Python (v3.12). First, a Savitzky-Golay filter (with a window length of 5 days) was applied to remove erroneous data caused by instrument noise and sensor errors. Second, we used Random Forest and Bayesian ridge regression models to interpolate missing data based on the complete data from adjacent depths or observational sites, and selected the optimal model based on its performance (Wang *et al.*, 2023[87]). Mean R^2 and root mean square error of the models used for final data reconstruction were 0.97 and 0.15, respectively.

The corresponding details have been added to the ***Methods Section***, as follows:

Lines 439-446: "To address missing or erroneous data of Tp, we implemented machine learning algorithms using the scikit-learn library in Python (v3.12). First, a Savitzky-Golay filter (with a window length of 5 days) was used to remove erroneous data caused by instrument noise and sensor errors. Second, we employed Random Forest and Bayesian ridge regression models to interpolate missing data based on the complete data from adjacent depths or observational sites, and selected the optimal model based on its performance[87]. Mean R^2 and root mean square error of the models used for final data reconstruction were 0.97 and 0.15, respectively."

Reference:

87. Wang, Y. et al. Persistent and enhanced carbon sequestration capacity of alpine grasslands on Earth's Third Pole. *Sci. Adv.* **9**, eade6875 (2023).

Minor: Supplementary Fig 1 would be good to have in the main body.

[Response]: Thanks! We have moved Supplementary Fig. 1 to the main body as Fig. 1 in the revised manuscript.

Response to the Reviewers' Comments

We sincerely appreciate the positive assessment of our work and insightful advice. We have made changes to the revised manuscript based on your constructive comments and suggestions. Our point-by-point responses to each comment and suggestion are as follows. Comments and suggestions are in black *italics*, our responses are in blue.

Response to Reviewer #1

The authors have made substantial revisions in response to the previous review comments. I find that all of my concerns and suggestions have been adequately addressed. The manuscript has been significantly improved in terms of clarity, rigor, and presentation. Therefore, I recommend that the manuscript be accepted for publication in its current form.

[Response]: We sincerely thank you for your positive evaluation and recommendation for publication. We are deeply grateful for the time and effort you have devoted to reviewing our manuscript throughout the revision process. Your constructive concerns and insightful suggestions have been invaluable in helping us improve the clarity, rigor, and overall presentation of the manuscript.

Response to Reviewer #2

The authors have well addressed my previous concerns.

[Response]: We greatly appreciate your positive assessment of our revised manuscript. Thank you again for your valuable comments and suggestions during the previous round of review, which helped us refine the manuscript and strengthen its clarity and quality.

Response to Reviewer #3

I would like to suggest that in future studies the authors do sequence negative controls despite insufficient material. It is still possible to make libraries with 'undetected' DNA in negative controls and sequence them (you treat it as if you have enough material), there are always some reads that come out of these negative (insufficient DNA) libraries, which can be used to verify the negative controls or to potentially remove some taxa from the downstream analysis.

[Response]: Thank you for this insightful suggestion. We agree that sequencing negative controls with DNA below the detection limit to assess low-level contamination and to identify potential contaminant taxa for exclusion from downstream analyses. In the present study, the negative controls did not yield detectable PCR amplification. Therefore, sequencing was not performed for these controls. We will adopt this practice in our future metagenomic studies to further strengthen contamination assessment and minimize the potential impact of contamination on downstream analyses.

2) I'm not sure its needed for this manuscript at this point (though would be nice for Nat Comms!), but since the authors did co-assemblies and individual assemblies for binning MAGs, a good (hybrid) strategy would be to use MAGs from both of these strategies and dereplicate them all together, then retain the dereplicated ones. Potentially some of the MAGs from the co-assemblies would be unique or higher quality compared to ones from individual assemblies (potentially from the lower abundance taxa). This would maximise the amount/quality of MAGs recovered without any sacrifice.

[Response]: We sincerely thank the reviewer for this constructive suggestion. In response, we combined the MAGs obtained from single-sample assembly (501 MAGs) with those from co-assembly (172 MAGs), resulting in a total of 673 medium- and high-quality MAGs for dereplication at the species-level (95% ANI) using dRep (v3.4.2). The single-sample assembly dataset yielded 282 species-level genome bins (SGBs), the co-assembly dataset yielded 155 SGBs, whereas the merged non-redundant catalog contained 336 SGBs. Relative to the single-sample assembly dataset, the hybrid strategy recovered 54 additional unique SGBs, expanding the non-redundant genome catalog. Additionally, the hybrid catalog showed improved quality,

as indicated by a lower average contamination rate (4.19% vs. 4.39%) and a higher average N50 (15.32 kb vs. 14.33 kb) compared with the single-sample assembly catalog. In line with the FAIR principles (Findable, Accessible, Interoperable, and Reusable) for data sharing, we have deposited the merged set of 336 SGBs in the Figshare as an additional openly accessible resource (https://doi.org/10.6084/m9.figshare.32769480).

In addition, we also evaluated whether this increased SGB count would affect the core diversity patterns in our study. The median Shannon index across all samples changed from 2.046 to 2.152 while Spearman $\rho = 0.95$, and the median Simpson index changed from 0.7078 to 0.7142 while Spearman $\rho = 0.92$. These differences were negligible and would be very unlikely to alter any of our statistical conclusions or ecological interpretations. Given these marginal differences, we have decided to retain our original SGB catalog (derived from single-sample assemblies) for all analyses presented in the manuscript.

Thank you again for your forward-looking suggestion and we will give fully consideration to hybrid strategy in the future.
